# Supplementary material for: Adding pattern and process to eco-evo theory and applications
Source: PLoS One. 2023 Mar 9;18(3):e0282535. doi: 10.1371/journal.pone.0282535 (PMC9997922; doi:10.1371/journal.pone.0282535)
Supplement: Attachment — Submitted filename: Response to Reviewers.pdf [file pone.0282535.s002.pdf]

Dear Dr. Bhardwaj,

Accompanying this letter, we are submitting a revised version of our manuscript: PONE-S-22-26399, titled *Adding Pattern and Process to Eco-Evo Theory and Applications*. You sent us your own comments, and forwarded two reviews of our manuscript, all of which we respond to below.

Response to Academic Editor

Your editorial comments were limited to passing along two URLs directing us towards PLoS style guides. Thank you for sending these links -- the revised manuscript now corrects multiple instances in which the original draft failed to adhere to the PLoS guidelines. We apologize for not getting those details correct in our original submission. Specifically, we have:

- Added line numbers.
- Corrected the formatting of the title page.
- Corrected the formatting of the headings and subheadings.
- Removed figures themselves, and relocated and reformatted figure captions.
- Relocated the tables and reformatted their captions.

We have also made several minor edits to the manuscript to improve clarity:

1. Abstract [] Lines 18-19

   <u>Changed</u>: “this simplification” <u>To</u>: “such simplifications”

2. Introduction / Population Genetics [] Lines 90-91

   <u>Changed</u>: “local adaptation), and gene-flow is governed by”

   <u>To</u>: “local adaptation), or when gene-flow is governed by”

3. Methods [] Line 135, 137, 138

   <u>Changed</u>: “simple simulation” <u>To</u>: “simple heuristic simulation”

   <u>Changed</u>: “simulated species” <u>To</u>: “single species”

   <u>Changed</u>: “an interaction” <u>To</u>: “the interactions”

4. Methods / Individual Ecological Characteristics [] Lines 166-169

   <u>Changed</u>: “corresponding to a year, with a sequence of life history events and species-landscape interactions performed at each step. In brief, the life cycle”

To: “corresponding to one year, with a sequence of life history events and species-landscape interactions performed at each step. Note that we subsequently use the terms "time step" and "year" synonymously. We favor "time step" when describing model mechanics, and "year" when interpreting simulation output. In brief, the life cycle”

5. Methods / Landscape Genetics [] Line 255

   Changed: “function with” To: “function with the”

6. Methods / Conservation Biology [] Line 292 & 293

   Changed: “of n alleles” To: “allele”

   Changed: “number extant alleles” To: “number of extant alleles”

7. Results / Landscape Genetics [] Lines 339-345

   Changed: “years 1-1000 (H1), years 1-2000 (H2), years 1-3000 (H3), and years 1-4000 (H4). Genetic distance matrices observed at the conclusion of each epoch illustrate the effectiveness of gene-flow at mixing subpopulations, given landscape history and dispersal behavior (Fig 4). Additionally, barrier gap permeability affects gene-flow in H3 & H4, and selection strength affects gene-flow in H4.”

   To: “years 1-1000, years 1-2000, years 1-3000, and years 1-4000. Genetic distance matrices observed at the conclusion of each epoch illustrate the effectiveness of gene-flow at mixing subpopulations, given landscape history and dispersal behavior (Fig 4). Additionally, barrier gap permeability affects gene-flow in the *Semi-Connected* and *Semi-Connected with Local Selection* epochs, and selection strength affects gene-flow in the *Semi-Connected with Local Selection* epoch.”

8. Results / Fig. 4 Caption [] Line 347

   Changed: “of” To: “incorporating”.

9. Results / Evolutionary Ecology [] Line 417

   Changed: “non-zero” To: “nonzero”.

   Changed: “time step 3000-5000” To: “time steps 3000-5000”.

10. Discussion [] Lines 444 & 446

   Changed: “responses such as” To: “responses including”.

   Changed: “including generation time” To: “for example generation time”.

11. Discussion & Conclusions [] Line 510 & 538

    Changed: “species ranges” To: “species’ ranges”.

We hope that you find that the revised version of our manuscript better adheres to PLOS’ standards. Naturally, we are happy to make further stylistic modifications, as necessary. But the revised MS certainly represents a huge improvement in this regard.

Please note that these changes were not requested by the manuscript reviewers. For that reason, we are not submitting a copy of our manuscript that utilizes the Microsoft Track Changes feature.

Response to Reviewer-2

We’ve decided to begin with Reviewer-2, for simplicity. This review was comprised of the following text:

> Eco-evolutionary and the lesser understood, landscape dynamics have a complex relationship that influence population responses. The authors have attempted to provide an improved simulation modeling approach to understand these phenomena. They further tested and altered fundamental assumptions and patterns of evolutionary forces and thereafter, observed responses to these manipulations. In my opinion, this manuscript and the resulting simulation has the potential to provide further insights in the existing eco-evolutionary theory and henceforth, its applications.

These comments constitute more a recapitulation of the manuscript’s goals rather than an evaluation of its merit. Nonetheless, the tone is positive, and this reviewer’s responses to the four standardized questions were all supportive. Our interpretation (in the absence of any evidence to the contrary) is that Reviewer-2 did not have any recommendations for manuscript changes, and is in favor of publication. In many regards, we (the authors) hold ourselves to a higher standard when providing manuscript reviews for journals. At the same time, we appreciate that reviewers are volunteers, and have many demands on their time. Our thanks go to Reviewer-2 for taking the time to provide the critique you forwarded.

Response to Reviewer-1

Regarding whether our manuscript was technically sound, and statistically rigorous, Reviewer-1 appeared dubious. In addition, Reviewer-1 offered the following comments:

1. The research paper entitled “Adding Pattern and Process to Eco-Evo Theory and Applications. Short Title: Eco-Evo Theory and Applications” by Schumaker et al. presented a novel simulation modeling approach for investigating eco-evolutionary dynamics, centered on the driving role of landscape pattern. The manuscript is hard to catch what is the exact subject of the study described.

2. The introduction is also vague and somewhat confusing, and is not organized in a scientific fashion. What is the background of the research, why does it need to be investigated?

3. Introduction and conclusion are not at par with the hypothesis.

4. The methods are not clear and are very difficult to understand. It could be a little simple and illustrative.

5. Discussion can be improved. There is no crosstalk in the discussion. You can discuss with some of the cross-references then it will make more sense of the study.

6. The overall text is not written clearly. There is too much of wordiness in the lines. It should be removed to make it simple and crisp.

7. The HexSim approach that you used, has a global significance in conservation management for in particular or for any ecosystem in general?

We discuss each of these items in detail below. But let us begin by asserting that we have concluded that Reviewer-1 (who provided his name, and who we looked up via Google searches) apparently has no background in this type of research. Dr. Singh is a chemist and biochemist involved in pharmacology and drug discovery. He hails from disciplines where research findings are disseminated in a comparatively rigid and standardized format. Dr. Singh’s comments make it clear he did not understand or appreciate our study, nor was he familiar or comfortable with the more creative and extemporaneous style in which ecological papers are often written. Below we critique Reviewer-1’s feedback in a series of nine points.

Point 1. In response to PLOS question 1 -- *Is the manuscript technically sound, and do the data support the conclusions?*, Reviewer-1 responded “*Partly*”. In reality, there are no elements of our study, nor are there or any passage in our manuscript, where our methods become vague or suspect, or where we fail to spell out clearly why we draw the

conclusions we do from the evidence we provide. We believe Reviewer-1 simply did not understand our study goals, design, methods, or conclusions.

Point 2. In response to the PLOS question 2 -- *Has the statistical analysis been performed appropriately and rigorously?*, Reviewer-1 replied "*I don't know*". We believe this response must be taken literally (as opposed to inferring the reviewer feels it is not *possible* to know). We assert that Reviewer-1 responded "I don't know" because he simply did not understand our manuscript.

Point 3. Reviewer-1, in comment #1, states: "*The manuscript is hard to catch what is the exact subject of the study described*". This comment is at odds with the manuscript itself. In point of fact, we summarize the entire study quite nicely in our Abstract. And we provide exceeding clear motivation for the study in the Introduction, including, for example, the following passage. In this passage, we introduce the reader to our overarching motivation and goals, laying a foundation for more detailed material that comes later in this section:

> Here, we illustrate how spatial structure drives eco-evo dynamics within four focal disciplines: Landscape Genetics, Population Genetics, Conservation Biology, and Evolutionary Ecology. We employ computer simulations to illustrate these dynamics using a novel modeling environment called HexSim [14] in which both biological forces and observable demographic and genetic responses emerge mechanistically from changes to landscape structure. HexSim life history processes are directly linked to static or dynamic landscape maps, and multiple spatial drivers can simultaneously influence different parts of the same simulation. Further, movement responses to landscape structure are not constrained by a reliance on resistance surfaces, patch-mosaic structures, stepping stones, or the use of graph-theoretic networks – all simplifications that are frequently employed to speed model development, but at a cost to biological realism. Lastly, HexSim eco-evo processes are mechanistically connected using a highly flexible system of demographic and genetic (hereafter "demo-genetic") life history traits. While more than 60 publications have resulted from HexSim-based studies (www.hexsim.net), adoption and application of the model's genetics toolkit has lagged. Below we describe how increasing attention to spatial and demographic details, and the further integration of ecological and evolutionary processes, will likely contribute to the focal disciplines mentioned above.

And, in standard form, we conclude the Introduction with an overview of what's to come:

> Below, we describe a relatively simple theoretical model designed to illustrate how the explicit inclusion of detailed spatial patterns and biologically realistic processes might facilitate the development of new eco-evo theory and applications. We

> demonstrate model relevance by highlighting results that (a) illustrate *a priori* expectations of a core eco-evo dynamic fundamental to a discipline, and (b) illustrate how additional biological and spatial realism might contribute to new theory and improve our confidence in modeling applications (Table 1). Our primary goals include both evaluating and articulating the benefits derived from adding spatial structure to genetic models, and illustrating the mechanics involved in doing this well. While the incorporation of space can substantially enhance realism and defensibility, it also complicates models, especially those which are already mechanistically-rich (e.g., that integrate demography, movement, and gene flow). In pursuit of the former goal, we trade away elements of realism, in the form of landscape structure and movement behavior, for mechanistic clarity. Readers evaluating our assertions will benefit from the simplicity of our simulations, as the number of drivers and responses they must track is limited. In regards to the latter goal, our work constitutes an important extension to the existing descriptions and applications of HexSim [14]. Readers interested in adopting this software will benefit from the case studies discussed here, as they provide detail on HexSim genetics that is unavailable in previous publications.

We reiterate our study goals in the first paragraph of the Discussion, and throughout the Conclusions. In fact, we have gone to great lengths to help readers understand exactly what motivated our study, its design and execution, and how we arrived at our conclusions. Our manuscript is well written, our figures and tables are thoughtfully designed, and nowhere is our meaning obfuscated by poor writing, vague statements, excessive jargon, or cognitive gaps.

Point-4. Reviewer-1, in comment #2, states: "*The introduction is also vague and somewhat confusing, and is not organized in a scientific fashion. What is the background of the research, why does it need to be investigated?*". We find these comments unfounded and somewhat bizarre; see above.

Point-5. Reviewer-1, in comment #3, states: "*Introduction and conclusion are not at par with the hypothesis*". Here, Reviewer-1's descent into subjectivity appears to accelerate. It is impossible to know what Reviewer-1 means by this statement, and there is nothing actionable therein, to unpack and address. We have no idea how to respond to this odd commentary.

Point-6. Reviewer-1, in comment #4, states: "*The methods are not clear and are very difficult to understand. It could be a little simple and illustrative*". We believe this statement strongly reinforces our earlier conjecture that Reviewer-1 is unfamiliar with scientific writing outside the fields of pharmacy, and possibly biochemistry. In fact, we have edited and re-edited this manuscript at least a dozen times, doing everything we can to make it as

informative and transparent as possible. The same is true for our figures and tables, which we have worked hard to make visually and conceptually clear. We have gone to these great lengths specifically because one of our principal goals in this work is to inspire young researchers (graduate students and postdocs) to adopt and utilize our approach. We feel strongly that the models, methods, and techniques we introduce here have the potential to help initiate a large number of exciting, timely, and important new studies, and we wanted to ensure that we did everything in our part to appeal to this audience. Well-written papers, such as ours, generate higher rates of uptake than do poorly written ones.

Further, we believe our Methods section constitutes a welcome departure from the norm, in that we have expunged all model-specific jargon, and we have worked hard to strike a balance between information density and readability. To the extent possible, we have also written our methods in a conversational format, and tried to ensure they are approachable by readers having a range of backgrounds.

Point-7. Reviewer-1, in comment #5, states: "*Discussion can be improved.There is no crosstalk in the discussion. You can discuss with some of the cross-references then it will make more sense of the study*". We have no idea what the reviewer is attempting to communicate here. But we counter that our Discussion section is very nicely written, and is quite clear. Our Discussion builds upon the motivation developed in the Introduction, and it thoroughly reexamines observations made in our Results. Assuming the standard interpretation of the word "crosstalk", we would suggest that there is extensive use of that concept reflected in our Discussion. Though we remain unclear regarding how to interpret his words, we nevertheless struggle to understand how Reviewer-1 could come away from our manuscript with such an impression.

Point-8. Reviewer-1, in comment #6, states: "*The overall text is not written clearly. There is too much of wordiness in the lines. It should be removed to make it simple and crisp*". I (NHS) was an editor at Landscape Ecology for 8 years. I review a lot of papers. I train graduate students and postdocs in scientific writing all of the time. I have written or (mostly) co-authored many peer-reviewed scientific publications. This manuscript is -- without question -- the most elegant and thoughtfully written of all the scientific works I have had the privilege of contributing to. In this light, we find the reviewer's comments trite, tedious, and ill-informed. The irony stemming from the reviewer's inability to clearly express these thoughts in words is humorous; though, we would never fault a nonnative English speaker for failing to master the nuance and idiosyncrasies of our stunningly illogical language.

Point 9. Reviewer-1, in comment #7, states: "*The HexSim approach that you used, has a global significance in conservation management for in particular or for any ecosystem in general?*". Prior to penning this statement, Reviewer-1 might have considered visiting the

web page for HexSim, which we provide in the manuscript (lines 66 & 314). Had he done this, Reviewer-1 may have noticed the 67 downloadable, peer-reviewed, published research articles that illustrate the wide variety of existing HexSim model applications. As this comment doesn't actually contain a question, we will leave it there.

Concluding Thoughts

My co-authors and I worked diligently to ensure the manuscript we submitted to PLoS One would not waste your time, or that of any reviewers you selected. We did this by paying careful attention to every sentence, paragraph, and section. We devoted extensive effort to the clarity of our figures and tables. We worked hard to tell a consistent and compelling story, and ensure there were consistent throughlines connecting the manuscript's sections. We developed a manuscript that avoids jargon, and thoughtfully balances information density with readability. And we believe our revised manuscript will enjoy wide readership, and will inspire large numbers of both citations and spin-off research studies. In short, we feel our revised manuscript constitutes an exciting, timely, and relevant contribution to PLoS One, and that it will nicely complement the diverse array of content you have been publishing recently.

We very much appreciate your assistance getting our manuscript reviewed, and in correcting our formatting errors. But four months passed between the time we originally submitted our manuscript to PLoS One, and when we received these reviews. And regrettably, the feedback we obtained via the review process was notably lacking in substance (or in the case of Reviewer-1, in credibility). Thus, we very much hope the next step of the publication process can proceed as rapidly as possible.

We believe strongly that our revised manuscript is improved, that it meets the PLoS formatting guidelines, and that it constitutes a worthy contribution to PLoS One, <u>in its current state</u>. Please let us know, at your earliest convenience, how you plan to move forward with our submission.

Again, we thank you very much for your contribution to the publishing process generally, and our manuscript in particular.

Best wishes,

Nathan Schumaker, and co-authors
