# Supplementary material for: Adding pattern and process to eco-evo theory and applications
Source: PLoS One. 2023 Mar 9;18(3):e0282535. doi: 10.1371/journal.pone.0282535 (PMC9997922; doi:10.1371/journal.pone.0282535)
Supplement: Attachment — Submitted filename: PONE-D-22-20501_R2 REVISION.pdf [file pone.0282535.s005.pdf]

PLOS ONE

Adding Pattern and Process to Eco-Evo Theory and Applications

--Manuscript Draft--

**Manuscript Number:**	PONE-D-22-20501R2
**Article Type:**	Research Article
**Full Title:**	Adding Pattern and Process to Eco-Evo Theory and Applications
**Short Title:**	Eco-Evo Theory and Applications
**Corresponding Author:**	Nathan Schumaker<br>US Environmental Protection Agency<br>Corvallis, Oregon UNITED STATES
**Keywords:**	individual-based modeling; spatially-explicit modeling; movement behavior; conservation genetics; population genetics; landscape genetics; evolutionary ecology; HexSim
**Abstract:**	Eco-evolutionary dynamics result when interacting biological forces simultaneously produce demographic and genetic population responses. Eco-evolutionary simulators traditionally manage complexity by minimizing the influence of spatial pattern on process. However, such simplifications can limit their utility in real-world applications. We present a novel simulation modeling approach for investigating eco-evolutionary dynamics, centered on the driving role of landscape pattern. Our spatially-explicit, individual-based mechanistic simulation approach overcomes existing methodological challenges, generates new insights, and paves the way for future investigations in four focal disciplines: Landscape Genetics, Population Genetics, Conservation Biology, and Evolutionary Ecology. We developed a simple individual-based model to illustrate how spatial structure drives eco-evo dynamics. By making minor changes to our landscape’s structure, we simulated continuous, isolated, and semi-connected landscapes, and simultaneously tested several classical assumptions of the focal disciplines. Our results exhibit expected patterns of isolation, drift, and extinction. By imposing landscape change on otherwise functionally-static eco-evolutionary models, we altered key emergent properties such as gene-flow and adaptive selection. We observed demo-genetic responses to these landscape manipulations, including changes in population size, probability of extinction, and allele frequencies. Our model also demonstrated how demo-genetic traits, including generation time and migration rate, can arise from a mechanistic model, rather than being specified a priori. We identify simplifying assumptions common to four focal disciplines, and illustrate how new insights might be developed in eco-evolutionary theory and applications by better linking biological processes to landscape patterns that we know influence them, but that have understandably been left out of many past modeling studies.
**Order of Authors:**	Jennifer M. White
	Nathan Schumaker
	Rachel Y. Chock
	Sydney Watkins
**Response to Reviewers:**	Please see attached file "Response to Reviewers".
**Additional Information:**	
**Question**	**Response**
**Financial Disclosure**<br><br>Enter a financial disclosure statement that describes the sources of funding for the work included in this submission. Review the submission guidelines for detailed requirements. View published research	The authors received no specific funding for this work.

Powered by Editorial Manager® and ProduXion Manager® from Aries Systems Corporation

articles from *PLOS ONE* for specific examples.<br><br>This statement is required for submission and **will appear in the published article** if the submission is accepted. Please make sure it is accurate.<br><br>**Unfunded studies**<br>Enter: *The author(s) received no specific funding for this work.*<br><br>**Funded studies**<br>Enter a statement with the following details:<br>• Initials of the authors who received each award<br>• Grant numbers awarded to each author<br>• The full name of each funder<br>• URL of each funder website<br>• Did the sponsors or funders play any role in the study design, data collection and analysis, decision to publish, or preparation of the manuscript?<br>• **NO** - Include this sentence at the end of your statement: *The funders had no role in study design, data collection and analysis, decision to publish, or preparation of the manuscript.*<br>• **YES** - Specify the role(s) played.<br><br>* typeset	
**Competing Interests**<br><br>Use the instructions below to enter a competing interest statement for this submission. On behalf of all authors, disclose any competing interests that could be perceived to bias this work—acknowledging all financial support and any other relevant financial or non-financial competing interests.<br><br>This statement is required for submission and **will appear in the published article** if the submission is accepted. Please make sure it is accurate and that any funding sources listed in your Funding Information later in the submission form are also declared in your Financial Disclosure statement.	The authors have declared that no competing interests exist.

View published research articles from *PLOS ONE* for specific examples.<br><br>**NO authors have competing interests**<br><br>Enter: *The authors have declared that no competing interests exist.*<br><br>**Authors with competing interests**<br><br>Enter competing interest details beginning with this statement:<br><br>*I have read the journal's policy and the authors of this manuscript have the following competing interests: [insert competing interests here]*<br><br>* typeset	
**Ethics Statement**<br><br>Enter an ethics statement for this submission. This statement is required if the study involved:<br><br>• Human participants<br>• Human specimens or tissue<br>• Vertebrate animals or cephalopods<br>• Vertebrate embryos or tissues<br>• Field research<br><br>Write "N/A" if the submission does not require an ethics statement.<br><br>General guidance is provided below. Consult the submission guidelines for detailed instructions. **Make sure that all information entered here is included in the Methods section of the manuscript.**	N/A

**Format for specific study types**<br><br>**Human Subject Research (involving human participants and/or tissue)**<br>• Give the name of the institutional review board or ethics committee that approved the study<br>• Include the approval number and/or a statement indicating approval of this research<br>• Indicate the form of consent obtained (written/oral) or the reason that consent was not obtained (e.g. the data were analyzed anonymously)<br><br>**Animal Research (involving vertebrate animals, embryos or tissues)**<br>• Provide the name of the Institutional Animal Care and Use Committee (IACUC) or other relevant ethics board that reviewed the study protocol, and indicate whether they approved this research or granted a formal waiver of ethical approval<br>• Include an approval number if one was obtained<br>• If the study involved *non-human primates*, add *additional details* about animal welfare and steps taken to ameliorate suffering<br>• If anesthesia, euthanasia, or any kind of animal sacrifice is part of the study, include briefly which substances and/or methods were applied<br><br>**Field Research**<br><br>Include the following details if this study involves the collection of plant, animal, or other materials from a natural setting:<br>• Field permit number<br>• Name of the institution or relevant body that granted permission	
**Data Availability**<br><br>Authors are required to make all data underlying the findings described fully available, without restriction, and from the time of publication. PLOS allows rare exceptions to address legal and ethical concerns. See the PLOS Data Policy and FAQ for detailed information.	Yes - all data are fully available without restriction

A Data Availability Statement describing where the data can be found is required at submission. Your answers to this question constitute the Data Availability Statement and **will be published in the article**, if accepted.<br><br>**Important:** Stating 'data available on request from the author' is not sufficient. If your data are only available upon request, select 'No' for the first question and explain your exceptional situation in the text box.<br><br>Do the authors confirm that all data underlying the findings described in their manuscript are fully available without restriction?	
**Describe where the data may be found in full sentences. If you are copying our sample text, replace any instances of XXX with the appropriate details.**<br><br>• If the data are **held or will be held in a public repository**, include URLs, accession numbers or DOIs. If this information will only be available after acceptance, indicate this by ticking the box below. For example: *All XXX files are available from the XXX database (accession number(s) XXX, XXX.*).<br>• If the data are all contained **within the manuscript and/or Supporting Information files**, enter the following: *All relevant data are within the manuscript and its Supporting Information files.*<br>• If neither of these applies but you are able to provide **details of access elsewhere**, with or without limitations, please do so. For example:<br><br>*Data cannot be shared publicly because of [XXX]. Data are available from the XXX Institutional Data Access / Ethics Committee (contact via XXX) for researchers who meet the criteria for access to confidential data.*<br><br>*The data underlying the results presented in the study are available from (include the name of the third party*	Our study is heuristic, and does not make use of any actual empirical data. However, our entire model, including all requisite inputs, will be made freely available at www.hexsim.net. We have a collection of models available at that website.

and contact information or URL).<br>• This text is appropriate if the data are owned by a third party and authors do not have permission to share the data.<br><br>* typeset	
Additional data availability information:	

Manuscript Click here to access/download;Manuscript;Manuscript.docx

Adding pattern and process to eco-evo theory and applications

Jennifer M. White [1]
Nathan H. Schumaker [2]*
Rachel Y. Chock [3]
Sydney M. Watkins [4]

[1] Center for Conservation Biology, Department of Biology, University of Washington, Seattle, Washington, United States of America

[2] U.S. Environmental Protection Agency, Pacific Ecological Systems Division, Corvallis, Oregon, United States of America

[3] San Diego Zoo Wildlife Alliance, Conservation Science, Escondido, California, United States of America

[4] Oak Ridge Institute for Science and Education, Oak Ridge, Tennessee, United States of America

*Corresponding author:
E-mail: nathan.schumaker@gmail.com (NHS)

Abstract

Eco-evolutionary dynamics result when interacting biological forces simultaneously produce demographic and genetic population responses. Eco-evolutionary simulators traditionally manage complexity by minimizing the influence of spatial pattern on process. However, such simplifications can limit their utility in real-world applications. We present a novel simulation modeling approach for investigating eco-evolutionary dynamics, centered on the driving role of landscape pattern. Our spatially-explicit, individual-based mechanistic simulation approach overcomes existing methodological challenges, generates new insights, and paves the way for future investigations in four focal disciplines: Landscape Genetics, Population Genetics, Conservation Biology, and Evolutionary Ecology. We developed a simple individual-based model to illustrate how spatial structure drives eco-evo dynamics. By making minor changes to our landscape's structure, we simulated continuous, isolated, and semi-connected landscapes, and simultaneously tested several classical assumptions of the focal disciplines. Our results exhibit expected patterns of isolation, drift, and extinction. By imposing landscape change on otherwise functionally-static eco-evolutionary models, we altered key emergent properties such as gene-flow and adaptive selection. We observed demo-genetic responses to these landscape manipulations, including changes in population size, probability of extinction, and allele frequencies. Our model also demonstrated how demo-genetic traits, including generation time and migration rate, can arise from a mechanistic model, rather than being specified *a priori*. We identify simplifying assumptions common to four focal disciplines, and illustrate how new insights might be developed in eco-evolutionary theory and applications by better linking biological processes to landscape patterns that we know influence them, but that have understandably been left out of many past modeling studies.

Introduction

Eco-evolutionary dynamics are shaped by spatial patterns and mediated by movement. The influence of spatial pattern on eco-evolutionary (hereafter "eco-evo") dynamics spans spatio-temporal scales and scientific disciplines, from cancer development within populations of somatic cells [1] to ecosystem responses to climate change (e.g., [2, 3]). With advances in molecular genetics, the time scales between evolutionary and ecological forces have been shown to be sufficiently coincident [4] and feedbacks from rapid-evolution impinging on ecological processes have been directly observed in an increasing number of natural systems [5-11]. For example, evolutionary geneticists follow the evolution of heritable traits responding to spatially heterogeneous selection pressures, population geneticists examine changes in allele frequencies influenced by spatially-restricted gene-flow, phylogeographers investigate coalescent timing across glaciations, and conservation biologists explore links between genetic diversity and resilience in spatially isolated populations. Despite the inherently spatial nature of these processes, the dynamics of landscape structure are rarely considered. But landscape dynamics may be critical for understanding reciprocal feedbacks between ecology and evolution [12], and for informing management applications [13].

Here, we illustrate how spatial structure drives eco-evo dynamics within four focal disciplines: Landscape Genetics, Population Genetics, Conservation Biology, and Evolutionary Ecology. We employ computer simulations to illustrate these dynamics using a novel modeling environment called HexSim [14] in which both biological forces and observable demographic and genetic responses emerge mechanistically from changes to landscape structure. HexSim life history processes are directly linked to static or dynamic landscape maps, and multiple spatial drivers can simultaneously influence different parts of the same simulation. Further, movement responses to landscape structure are not constrained by a reliance on resistance surfaces, patch-mosaic structures, stepping stones, or the use of graph-theoretic networks – all simplifications that are frequently employed to speed model development, but at a cost to biological realism. Lastly, HexSim eco-evo processes are mechanistically connected using a highly flexible system of demographic and genetic (hereafter "demo-genetic") life history traits.

While more than 60 publications have resulted from HexSim-based studies (www.hexsim.net), adoption and application of the model's genetics toolkit has lagged. Below we describe how increasing attention to spatial and demographic details, and the further integration of ecological and evolutionary processes, will likely contribute to the focal disciplines mentioned above. Both the HexSim application and the simulation models described here are available at www.hexsim.net.

Landscape Genetics

This relatively new field endeavors to describe how landscape pattern influences gene-flow. A standard approach is to compare inter-individual genetic distance to metrics of landscape structure, often quantified as a cost-distance across a resistance surface. Available landscape genetics modeling platforms have had limited spatial, demographic, or behavioral sophistication [15], though some such constraints are being actively addressed by leaders in the field (e.g., [16]). Models that are unable to incorporate mechanisms underlying species-landscape interactions have a limited ability to simulate complex movements and resulting gene-flow. Additionally, most platforms cannot simultaneously simulate multiple interacting eco-evo drivers of gene-flow, such as local adaptation along with source-sink demographics [17].

Population Genetics

Investigators study the causes and consequences of population genetic structure. For simplicity, complex biological forces such as selection and mutation rates are often assumed to be spatially constant. Additionally, in classic population genetic models, spatial complexity is reduced to discrete populations, and migration processes are characterized by a single univariate input parameter, '*m*'. These simplifications have facilitated the generation of a wealth of theory; but, a lack of biological realism and spatial complexity have precluded the application of this theory to systems in which space is heterogeneous and dynamic (e.g., where landscape structure influences population size, density, dispersal flux, or local adaptation), or when gene-flow is governed by complex movement and mating behavior (e.g., when dispersal rates and distances are unequal, or mate-choice is non-random; see [18] for review).

Conservation Biology

This broadly defined discipline frequently involves the exploration of forces affecting population viability. Landscape pattern drives population viability through its influence on ecological and evolutionary processes. The relative importance of demography versus genetics has been actively debated in the conservation literature [19, 20]. Some forecasting tools can simulate either inbreeding rates or landscape use, and incorporate them into probabilities of extinction (e.g., *RAMAS GIS* [21] or *Vortex* [22]), but existing models simplify the linkages between ecological and evolutionary processes, and these shortcomings limit the utility of their forecasts. For example, a platform capable of simulating complex interacting demographic and genetic traits is necessary to forecast how landscape changes will alter a population's size and distribution (eco), while simultaneously impacting its mate-finding or natural selection (evo).

Evolutionary Ecology

Researchers explore interactions and feedback between ecological and evolutionary drivers that affect demographic and genetic traits. Landscape patterns shape myriad mechanisms through which biological forces influence population and community demo-genetic traits. Renewed interest in these processes (e.g., [23, 24]) has underscored the need for biologically sophisticated, mechanistic simulation platforms capable of explicitly modeling dynamic eco-evo feedback in a spatially-realistic setting. Simply modeling how environmental change impacts heritable traits is only half of the story; our long-term goals must also include asking how these emergent genetic traits connect back to ecological dynamics [25, 26].

Below, we describe a relatively simple theoretical model designed to illustrate how the explicit inclusion of detailed spatial patterns and biologically realistic processes might facilitate the development of new eco-evo theory and applications. We demonstrate model relevance by highlighting results that (a) illustrate *a priori* expectations of a core eco-evo dynamic fundamental to a discipline, and (b) illustrate how additional biological and spatial realism might contribute to new theory and improve our confidence in modeling applications (Table 1). Our primary goals include both evaluating and articulating the benefits derived from adding spatial structure to genetic models, and illustrating the mechanics involved in doing this well.

While the incorporation of space can substantially enhance realism and defensibility, it also complicates models, especially those which are already mechanistically-rich (e.g., that integrate demography, movement, and gene flow). In pursuit of the former goal, we trade away elements of realism, in the form of landscape structure and movement behavior, for mechanistic clarity. Readers evaluating our assertions will benefit from the simplicity of our simulations, as the number of drivers and responses they must track is limited. In regards to the latter goal, our work constitutes an important extension to the existing descriptions and applications of HexSim [14]. Readers interested in adopting this software will benefit from the case studies discussed here, as they provide detail on HexSim genetics that is unavailable in previous publications.

Table 1. How spatially structured eco-evolutionary models might contribute to four focal disciplines.

Discipline	Research Questions	Expected Outcomes	Anticipated Contributions
**Landscape Genetics**	How is gene-flow controlled by the landscape?	Isolation by Distance is a function of dispersal behavior.	Replacing resistance surfaces and cost-distance matrices with measures of genetic distance arising from eco-evo processes and species-landscape interactions.
**Population Genetics**	How is genetic structure controlled by the landscape?	Genetic structure is influenced by isolation by distance and demography.	Allowing migration rates between populations to emerge mechanistically from the interplay between dispersal behavior and landscape structure.
**Conservation Biology**	How are inbreeding and population viability controlled by the landscape?	Homozygosity and stochastic extinction are consequences of demographic processes.	Ensuring that forecasts of genetic degradation are driven by spatially-realistic movement models and incorporate pre-existing genetic structure and diversity.
**Evolutionary Ecology**	How are feedbacks between ecological and heritable traits controlled by the landscape?	Population demo-genetic traits are an ecological response to the evolutionary force of selection.	Creating eco-evo feedback loops between local selection for specific alleles and counteracting asymmetric migration stemming from source-sink dynamics in heterogeneous landscapes.

Methods

Our study was built around a simple heuristic simulation constructed within the HexSim modeling environment [14]. HexSim is a user-friendly, spatially-explicit, individual-based, demo-genetic modeling environment. We modeled a single species, and parameterized its life history, demographics, genetic traits, and the interactions between habitat type and genetics (described below). We created a minimally-complex landscape structure that varied episodically throughout our simulations. Each epoch (1,000 simulation time steps) included

unique barriers, barrier gaps, or habitat types. We varied behavior (dispersal distance), strength of selection, and barrier gap permeability for a total of eight treatment combinations, and tracked individual genotypes, per-capita homozygosity, per-patch population size, and the number of dispersers moving between the patches in each treatment. Through the use of this single model, we were able to probe expected outcomes of the four focal disciplines, and test the anticipated behaviors of this eco-evo modeling approach.

Landscape Structure

Our landscape was composed of six adjacent habitat patches (two small, two intermediate, two large) built up from multiple hexagonal cells of uniform quality (Fig 1). Two large adjoining patches (1326 hexagons each) lie at the landscape center. Two medium-sized patches (200 hexagons each) and two small patches (50 hexagons each) abut the large patches, but not each other. Patch dimensions expressed as columns × rows, were 26 × 51, 10 × 20, and 5 × 10. Collectively, the six patches were assembled from 3152 individual hexagons. Our simulated individuals were never allowed to enter the surrounding non-habitat matrix. Movement barriers were used, at times, to isolate the patches from each other. At other times, small semi-permeable openings in the barriers allowed limited dispersal between neighboring patches. The total size of the barrier gaps between adjacent patches was identical, ensuring individuals had a uniform potential for crossing all patch interfaces.

Fig 1. The progression of landscape epochs over 4000 simulation time steps. Movement barriers (shown as dark blue lines) and landscape edges are reflective. The six patches are distinguished by color within the *Continuous* epoch. Yellow barrier gaps, introduced at time step 2001, are of the same total length for each barrier. At time step 3001, the landscape is assigned two distinct habitat types (light blue vs. orange) that confer genotype-specific adaptive advantage to juvenile survival.

Individual Ecological Characteristics

Our simulations ran for a series of time steps, each corresponding to one year, with a sequence of life history events and species-landscape interactions performed at each step. Note that we

subsequently use the terms "time step" and "year" synonymously. We favor "time step" when describing model mechanics, and "year" when interpreting simulation output. In brief, the life cycle consisted of (a) resource acquisition, (b) pair formation, (c) reproduction, (d) juvenile dispersal, and (e) survival. The individuals making up our population included both sexes and two age classes (juvenile and adult), and our simulations began with the landscape being saturated with adults (3152 individuals spread uniformly across all patch hexagons). Adult females reproduced only if they could pair exclusively with an adult male located nearby (neither females or males had multiple mates). Individuals were assigned normally distributed reproductive rates, with mean values based on their resource allocation, which introduced a density-dependent feedback that was further modified by the requirement for pair-formation. Individuals acquired resources from a roughly-circular neighborhood of 37 hexagonal cells, and resources were shared equally by all individuals attempting to utilize them (scramble competition). Juveniles dispersed from their natal site in the year of their birth, and transitioned to adults at the start of the subsequent year. Adults did not move. Dispersal path lengths were drawn from one of two uniform distributions, either “short” (1-5 hexagon steps) or “long” (5-25 hexagon steps). Dispersal autocorrelation was set at 75, on a scale of 0 (completely random) to 100 (perfectly linear). Individual dispersers took a series of steps from hexagon to adjacent hexagon, and stopped when their path length had been reached. Yearly survival probability was based on stage class (juvenile = 0.500, adult = 0.885), but individuals with less than 20% of their resource goal were assigned an additional 10% probability of mortality. Our simulations included a period during which survival decisions were also based on genetic adaptation. In these cases, emergent mortality rates became jointly determined by stage class, location, and genotype (see below).

Individual Evolutionary Characteristics

Our simulated individuals were diploid with ten loci and zero linkage between loci. Each locus was assigned five alleles labeled A1-A5. The starting population’s allele assignment was governed by locus-specific initial allele frequencies (Table 2). Initial allele frequencies were not spatially stratified, and we did not simulate mutation. Offspring genotypes were assembled by

drawing a single allele from each parent at each locus. Individuals possessed five purely neutral loci (L1-L5), and five loci (L6-L10) containing at least one allele capable of conferring a fitness advantage. These locally adaptive alleles imparted a survival benefit to juveniles when they were in the habitat type to which they were genetically pre-adapted. The survival benefit (*S*) per adaptive allele was either strong (*S* = 0.10) or weak (*S* = 0.01). Modeled this way, selection was a predictable process because genotype dictated the mean juvenile survival probability. Selection was initiated at time step 3000. All ten loci were utilized for all genetic analyses described below, with the exception of adaptation, for which only L6-L10 were employed.

Table 2. Initial allele frequencies for 10-locus genotypes.

Locus	Allele	Initial Allele Frequency	Local Adaptation
1	1-5	0.20, 0.20, 0.20, 0.20, 0.20	Neutral
2	1-5	0.30, 0.25, 0.20, 0.15, 0.10	Neutral
3	1-5	0.10, 0.15, 0.20, 0.25, 0.30	Neutral
4	1-5	0.01, 0.04, 0.15, 0.30, 0.50	Neutral
5	1-5	0.50, 0.30, 0.15, 0.04, 0.01	Neutral
6	1	0.20	Neutral
	2	0.20	Locally Adapted - Habitat Type A
	3	0.20	Neutral
	4	0.20	Locally Adapted - Habitat Type B
	5	0.20	Neutral
7	1	0.30	Neutral
	2	0.25	Neutral
	3	0.20	Neutral
	4	0.15	Neutral
	5	0.10	Locally Adapted - Habitat Type A
8	1	0.10	Neutral
	2	0.15	Neutral
	3	0.20	Neutral

	4	0.25	Neutral
	5	0.30	Locally Adapted - Habitat Type B
9	1	0.01	Locally Adapted - Habitat Type A
	2	0.04	Neutral
	3	0.15	Neutral
	4	0.30	Neutral
	5	0.50	Neutral
10	1	0.50	Locally Adapted - Habitat Type B
	2	0.30	Neutral
	3	0.15	Neutral
	4	0.04	Neutral
	5	0.01	Neutral

Progression of Landscape Change

All of our landscapes were binary, consisting of habitat patches embedded within a non-habitat matrix. Our initial landscape was continuous and free from movement barriers. This landscape, hereafter referred to as *Continuous*, persisted for the first epoch of 1000 simulation time steps (Fig 1). We anticipated that Isolation by Distance (IBD) would be the predominant evolutionary force in this landscape. To simulate the effect of drift alone, we then imposed absolute movement barriers that isolated subpopulations into six separate landscape patches (two each, of three different sizes) for the subsequent 1000 simulation time steps. We refer to this epoch as *Isolated Patches*.

Following patch isolation, we created small gaps in the movement barriers, allowing infrequent migration between the previously isolated sub-populations. Barrier gaps varied in permeability (high = 0.70 transmission probability per encounter, low = 0.02 transmission probability per encounter), affecting the likelihood that individuals would cross the barrier when they encountered a gap during dispersal. We refer to this third epoch of 1000 time steps as *Semi-Connected*. During the final 1000 time steps, the patches were each assigned one of two

"habitat types" that conferred increased fitness to juveniles with specific genotypes. We refer to this landscape as *Semi-Connected with Local Selection*.

Treatments and Observable Responses

Individual dispersal behavior (long vs. short), strength of selection (strong vs. weak), and barrier gap permeability (low vs. high) together formed eight treatment combinations. Each treatment was repeated in ten replicates. For each treatment, we tracked individual genotypes, per-capita homozygosity, per-patch population size, and the number of dispersers moving between the patches. We did not track individual pedigree information. Upon completion of the simulations, we used HexSim's report generator to create files suitable for input to the genetic software package STRUCTURE [27], which we subsequently used for some of our analyses.

Adult females in our model could reproduce each time step until their death, thereby producing overlapping generations. We measured generation time as the observed average age of reproducing females [28]. Migration rates between patches were measured during the *Semi-Connected* epoch. To qualify as a migrant, individuals had to cross through a barrier gap and subsequently reproduce somewhere other than their natal patch. Our migration data thus intentionally excluded non-breeders and individuals that reproduced in their natal patch after making a temporary excursion elsewhere.

Landscape Genetics

Isolation by distance (IBD) produced by dispersal limitations [29] is a core concept in landscape genetics. We used results from the *Continuous* epoch to illustrate the degree of IBD for our short and long-distance dispersal treatment groups. We constructed dispersal kernels by plotting the frequency of observed dispersal distances for all individuals in all replicates, from time step 1 to 1000. We visually assessed the degree of IBD using correlograms generated by the application *Alleles in Space* [30], where the average genetic distance between individuals is plotted for varying distance classes. We also plotted the frequency of observations for each distance class, to ensure that the inter-individual genetic distances were not due to underlying distribution of individuals on the landscape.

We calculated inter-individual genetic-distances resulting cumulatively from each landscape history. For ease of illustration, we subset our results as follows: for each treatment group, we randomly selected a single replicate simulation. From the selected replicate, we extracted genotype reports from the last time step of each of the four landscape epochs. From those reports, we randomly selected 25 individuals from each of the six patch locations. For those 150 individuals, we calculated a simple metric of pairwise genetic distance (number loci for which alleles differ between individuals / total number loci) using the "*ape"* R (v3.2.2) package "*dist.gene"* function with the "*percentage"* method [31, 32]. We visualized the 150 x 150 pairwise genetic distances as triangular matrices.

Population Genetics

We used a Bayesian clustering method, implemented by the program STRUCTURE (v 2.3.4), to analyze population genetic structure [27]. At the end of each landscape epoch, we collected genotype information stratified by putative population (patch), and imported these data into STRUCTURE. Unequal sample sizes (small patches ≈ 25 individuals, large patches ≈ 1000) interfered with the assessment of the number of subpopulations and assignment probabilities. This known limitation of the software [33] was easily overcome by randomly drawing a fixed number of individuals (n=25) from each patch for analysis. In cases where there were fewer than 25 individuals extant in a given patch, genotypes were randomly selected for inclusion more than once until each patch had a sample of 25, for 150 total individuals.

We expected the number of unique genetic clusters (commonly referred to as "K") to vary from 1 to 6 based on landscape structure. Therefore, following convention, we tested possible values of K ranging from 1 to 20. All STRUCTURE analyses were run with a burn-in period of 10,000 iterations, with an additional 10,000 analysis iterations. We performed 20 replicate trials for each possible K value using the default settings of the admixture model and correlated allele frequencies. The best supported *K* values were identified using two methods: 1) plotting the replicate average *Ln P(D|K)*, and visually determining the minimum *K* of the curve's asymptote [33], and 2) using Evanno's Δ*K* method [34]. When there were close ties between supported *K*

values from the competing methods, we considered them all for individual assignment analyses.

Conservation Biology

We calculated *Per-capita Homozygosity* as the percent of homozygous genotypes across all alleles and individuals within a given landscape patch. Large values of *Per-capita Homozygosity* could result from a few highly inbred individuals or from many slightly inbred individuals. We also examined the effect of patch isolation on allele frequencies from data collected at the ends of the *Continuous* and *Isolated Patches* epochs. We calculated three metrics of genetic degradation for each locus in each subpopulation using output from the "'*diveRsity"* R package "*divBasic"* function [35], as described below:

1) *Allelic richness* = the number of unique alleles per locus. All ten loci began with 5 alleles each.

2) *Allelic evenness* = $\frac{\sum p_i \ln(p_i)}{\ln(5)}$, where p_i is the frequency of the ith allele within each locus, and $ln\ (5)$ is the maximum evenness in each locus, given that all were initiated with 5 alleles. The starting population had different initial allele frequencies corresponding to allelic evenness values of 1.0 = "Equal", 0.96 = "Unequal", and 0.72 = "Rare" (Table 2).

3) *Heterozygosity deficit* = $H_{obs} - H_{exp}$, where H_{obs} is the number of observed heterozygous genotypes and expected heterozygosity is calculated as: $H_{exp} = 1 - \sum_1^n (q_i)^2$, where n represents the number of alleles, and q_i is the frequency of the ith allele at a locus. Note that H_{exp} is calculated based on the number of extant alleles at a locus, and will fluctuate dramatically as alleles are lost from a population. Therefore, unlike *Allelic richness* or *Allelic evenness*, this metric has a shifting baseline.

For each metric, we calculated the mean and standard deviation across all replicates within the same patch size (small, medium, or large) and of the same initial allele frequency (equal, unequal, or rare) for each of the two time points of interest (at the end of the *Continuous* and *Isolated Patches* epochs).

Evolutionary Ecology

Population size was tracked for each patch across all time steps to facilitate measuring the response to adaptation. Additionally, we computed the change in frequency of adaptive alleles within each patch during the *Semi-Connected with Local Selection* epoch. L6 contained two adaptive alleles and 3 neutral, while L7-through L10 each contained one adaptive allele and 4 neutral. L6-A2, L7-A5 and L9-A1 were adaptive in half of the patches, while L6-A4, L8-A5 and L10-A1 were adaptive in the other half. Tracking allele frequencies in L6 allowed us to examine the effect of local adaptation on allele frequency in combination with asymmetric migration from adjacent patches, where the opposite allele was advantageous.

Results

The age-class structure that arose from our model resulted in an average age of reproducing females of 8.7 years. Therefore, each landscape epoch of 1000 simulation time steps (Fig 1) spanned approximately 115 generations. During the *Semi-Connected* epoch, migration rate between adjacent patches varied with dispersal distance, barrier gap permeability, and patch size (Fig 2); but regardless of these values, the majority of individuals remained in their natal patch. As anticipated, we observed higher migration rates when individuals were assigned the long-distance dispersal behavior and the barrier gaps were highly permeable. Unequal perimeter-area ratios ensured that migration was asymmetric, with smaller patches generating proportionately greater numbers of emigrants than larger patches, and larger patches receiving proportionately higher numbers of immigrants than smaller patches.

Fig 2. Observed migration rates of long or short-dispersing individuals across barriers of varying permeability during the *Semi-Connected* epoch. Left: Percent of the population that is born and reproduces in the same patch. Middle: Emigration rates, sorted by size of sending patch. Right: Immigration rates, sorted by size of receiving patch.

Landscape Genetics

The short and long-distance dispersal behaviors measured during the *Continuous* landscape epoch produced notably different dispersal kernels (Fig 3 *left*). IBD was evident in the

relationship between geographic distance and genetic distance when dispersal was short, but not when dispersal distances were long (Fig 3 *right*).

Fig 3. In a spatially-continuous landscape, the observed dispersal kernels reflected juvenile dispersal ability (left). Genetic IBD was evident when dispersal paths were short, but not when they became longer (right). These data were gathered at the end of the *Continuous* landscape epoch, after 115 generations of unobstructed movement.

We also interpret our series of landscape epochs as four nested hypothetical landscape histories: years 1-1000, years 1-2000, years 1-3000, and years 1-4000. Genetic distance matrices observed at the conclusion of each epoch illustrate the effectiveness of gene-flow at mixing subpopulations, given landscape history and dispersal behavior (Fig 4). Additionally, barrier gap permeability affects gene-flow in the *Semi-Connected* and *Semi-Connected with Local Selection* epochs, and selection strength affects gene-flow in the *Semi-Connected with Local Selection epoch*.

Fig 4. Modeling gene-flow for four landscape histories, beginning with IBD only, and building to a complex history of sequential epochs incorporating IBD, drift, migration, and finally, local selection. The resulting pairwise genetic distance matrices (colored triangles) illustrate the strength of gene-flow within a given landscape history, where increasing contrast in the geometric patterns visible within the matrices results from reduced gene-flow between landscape patches. Data collected at time step 4000 illustrate the interacting effects of gene-flow and local adaptation on genetic-distance. See Methods section for additional details.

Population Genetics

In the *Continuous* epoch, we know a single biological population was present on the landscape. But without the benefit, inherent in modeling, of knowing the true population dynamics, genetic analysis could easily lead to a conclusion that several distinct populations were present (Table 3). For example, neither Evanno's method or L(*K*) produced consistent results in this case. Short distance dispersal and the resulting IBD led to an inference of multiple genetic clusters under L(*K*), but a single genetic cluster under *E*. When dispersers moved longer

distances, and IBD was therefore absent, the L(*K*) method produced the expected result, but Evanno's method did not (Table 3).

Table 3. Inferred number of genetic subpopulations *K* resulting from either the visual interpretation of likelihood probabilities *L(K)* or from Evanno's ΔK method *E*, as a function of dispersal distance and epoch. When a single method produced multiple equally-supported estimates of K, then all values were included.

	K	Continuous Epoch	Isolated Patches Epoch
**Long Distance Dispersal**	1	L(K)	
	2	E	
	5		L(K)
	6		L(K)
	8		E
**Short Distance Dispersal**	2	E	
	4	L(K)	
	5	L(K)	L(K)
	6		L(K)
	7		E

In the *Isolated Patches* epoch, there are 6 distinct populations isolated by absolute barriers. By the end of the epoch, 5-8 unique genetic clusters were identified by our genetic analyses, regardless of dispersal behavior (Table 3). Evanno's method (*E*) tended to produce inflated estimates of *K* that exceeded the true number of 6 genetic subpopulations. The STRUCTURE plots (not shown) indicated clearly that the 4 smaller patches were each home to a single genetic cluster, made unique by drift. The two large patches housed either one or two genetic clusters, due to a weak effect of drift in these larger populations.

The population genetic structure produced by drift during the *Isolated Patches* epoch was mitigated by the limited inter-patch migration that characterized the subsequent *Semi-Connected* epoch (Table 4). The differing numbers of migrants per generation produced varying

degrees of genetic mixing over the course of these 115 generations. The observed >15 migrants per generation resulting from long distance dispersal and high barrier gap permeability produced sufficient genetic mixing to return to panmixia, while the other treatments failed to do so. Long-distance dispersal with low barrier permeability resulted in <1 migrant per generation, and this migration rate was insufficient to return the population to panmixia within the 115 generations of the epoch. A return to panmixia by the end of the *Semi-Connected* epoch was not expected in simulations employing a short dispersal distance, and no such outcome was observed.

Table 4. The replicated average number of observed migrants per generation, presented as a function of dispersal distance and barrier gap permeability, at the end of the *Semi-Connected* epoch. In most cases, population genetic structure (change in possible *K*, evaluated from either the visual interpretation of likelihood probabilities *L(K)* (top) or from Evanno's ΔK method *E* (bottom)) declined from where it began in the prior *Isolated Patches* epoch.

	**Low Barrier Gap Permeability**		**High Barrier Gap Permeability**	
	**Migrants per Generation**	**Change in Possible K**	**Migrants per Generation**	**Change in Possible K**
**Long Distance Dispersal**	0.757	5 or 6 → 4	15.456	5 or 6 → 1
		8 → 6		8 → 2
**Short Distance Dispersal**	0.086	5 or 6 → 6	2.598	5 or 6 → 6
		7 → 2		7 → 2

Conservation Biology

In this analysis, which included only the *Continuous*, *Isolated Patches*, and *Semi-Connected* epochs, per-capita homozygosity varied dramatically with landscape epoch, and fluctuated more in the small patches than the large ones (Fig 5). While we observed little accumulation of homozygosity during the *Continuous* epoch, this changed when migration between patches was

limited by dispersal barriers. During the *Isolated Patches* epoch, homozygosity increased rapidly, but this general trend was strongly influenced by patch size. Both patch size and barrier gap permeability affected the amount of "genetic rescue" resulting from migration during the *Semi-Connected* epoch.

Fig 5. Per-capita homozygosity across simulation time steps spanning the *Continuous*, *Isolated Patches*, and *Semi-Connected* landscape epochs, displayed by patch size. Our simulated low and high barrier crossing probabilities corresponded to P(transmission) values of 0.02 and 0.70, respectively. Six lines per plot result from two small, two medium, and two large patches.

Changes in allele frequencies observed during the *Continuous* and *Isolated Patches* epochs were affected by patch size, locus-specific initial allele frequencies, and dispersal distance. Even during the *Continuous* epoch, prior to patch isolation, rare alleles were lost due to drift. As anticipated, this process became more pronounced when dispersal was limited by impermeable barriers (the *Isolated Patches* epoch). The loss of allelic richness and evenness during patch isolation was most pronounced in the small patches, and least severe in the large patches. Short-distance dispersal also influenced loss of allelic richness and evenness in both epochs, but only in medium and small patches. A heterozygosity deficit was observed when dispersal distances were short, but only in large and medium patches.

Evolutionary Ecology

Selection pressure was initiated at the beginning of the final landscape epoch, at time step 3000. Effective carrying capacity and population sizes emerged mechanistically from our model. Population size was most stochastic in the smaller patches, and most stable in the large patches (Fig 6). A nonzero probability of extinction was observed only for the small patches, and was greatest when the dispersal distances were short and movement barriers were present. The simulated adaptation to local conditions (time steps 3000-5000) effectively increased patch carrying capacity. These effects of adaptation were more pronounced when selection pressure was strong.

Fig 6. Observed trends in population size stratified by patch size. Results from all ten simulation replicates are shown. Data are displayed for an additional 1000 time steps during the final landscape epoch in order to better visualize the long-term population response to selection pressure. Carrying capacity was effectively increased by local adaptation, but the magnitude of this effect depended on the strength of selection. Extinction events can be inferred from occasional very low population sizes observed for the small patches (note the logarithmic vertical axis).

The frequency of the adaptive alleles at loci L7 and L10 increased globally across all subpopulations as selection acted locally and migration moved the adaptive alleles across the landscape. However, the effect of local selection at Locus L6 was counteracted by asymmetric migration (see Fig 2) between adjacent patches of different habitat types. In small patches, local selection was almost always swamped by migration from the neighboring large patches, except when selection was strong, dispersal distance was short, and gap permeability was low. Otherwise, for the small and medium patches, the extent to which selection was swamped by migration varied more continuously based on the strength of selection, barrier gap permeability, and dispersal distance. In large patches, selection was often able to act effectively regardless of selection strength or migration rate, except when selection was weak and dispersal distance was long.

Discussion

We attempted to develop the simplest individual-based model capable of clearly illustrating how spatial structure drives eco-evo dynamics. By imposing landscape change on otherwise functionally-static eco-evolutionary models, we were able to alter key emergent forces such as gene-flow, genetic drift, and adaptive selection, as well as demo-genetic responses including population size, probability of extinction, and allele frequencies. Our model also demonstrated how critical demo-genetic traits, for example generation time and migration rate, can arise from a parsimonious mechanistic model, rather than being specified *a priori*.

Landscape Genetics

A common approach in landscape genetics is to assume that the current genetic pattern is a result of landscape pattern (with some lag-time) represented by resistance surfaces. Hypothetical "resistance distances" between individuals are compared to actual genetic distances between sampled individuals (or groups), and tests are performed to infer which elements in the landscape most strongly influence gene-flow. But complex movement behavior and dynamic landscape histories are not easily captured by resistance surfaces, and this in turn limits our ability to identify causal processes and detect past gene-flow [36, 37].

We demonstrated how the cumulative effects of gene-flow, genetic drift, and selection can be simulated over several hypothetical landscape histories. The resulting inter-individual genetic distance matrices simultaneously captured the influences of dynamic landscape structure, dispersal behavior, and demography. Long-distance dispersal behavior tended to swamp any effect of landscape history when landscape connectivity was high. This suggests it may be difficult to identify past landscape discontinuities when dispersal distances are long relative to the spatial extent of the study area.

Population Genetics

An ongoing challenge for empirical population geneticists and phylogeneticists has been determining the number of populations via analysis of genetic clustering patterns. Our model demonstrates that small amounts of IBD inflate such inferences, and that popular statistical solutions for choosing between possible numbers of genetic clusters are prone to overestimation [38].

Our approach offers population geneticists a methodology that allows migration rates to emerge from species-landscape interactions. Our model demonstrates how a simple alteration of barrier gap permeability can produce departures from the "one-migrant-per-generation" theoretically-derived guideline for re-establishing genetic panmixia [39]. We observed that, with short-distance dispersal behavior, the inference of population structure was similar when that structure resulted from either IBD or from gene-flow following a period of isolation,

highlighting the difficulty in disentangling past genetic history from contemporary genetic structure.

Conservation Biology

Forecasting genetic degradation in small populations facing extinction is a challenging task that should ideally acknowledge the influences of both demographic and genetic processes. Our methodology addresses this challenge, as illustrated by two examples from our results. First, rare alleles were lost from the small patches, even when the landscape was fully connected; and the severity of this loss was more extreme when individuals dispersed short distances. Second, the loss of allelic evenness we observed exhibited different patterns depending on patch size. In the small patches, the allelic evenness eventually approached zero, due to single allele fixation, regardless of initial allele frequencies or dispersal distance. Conversely, in the large patches, allelic evenness varied depending upon initial conditions, but did not vary with dispersal distance. We observed a combined effect of dispersal distance and initial conditions in the medium patches, where limiting dispersal distance increased the loss of allelic evenness, but the steady-state depended on initial conditions. Traditional simulation approaches would associate a single trend with any given landscape.

These findings highlight how eco-evo simulators can add realism to the forecasts of genetic degradation used in conservation, and demonstrate how future studies might improve our understanding of inbreeding and outbreeding depression, better explore the relative importance of genetic versus demographic components of viability, and more.

Evolutionary Ecology

The HexSim modeling platform allows users to develop dynamic feedback loops linking evolutionary and ecological forces. Our model captured the counteracting forces of local selection and asymmetric migration from spatially proximal sub-populations. Adaptive alleles increased in frequency in spatially discontinuous landscape patches due to local selection, while migration rates between patches varied by patch size, dispersal behavior, and barrier gap permeability. The increase in adaptive alleles was most pronounced when selection was strong,

yet this effect was not observed in the small and medium-sized patches when dispersal distance was short and barrier gap permeability was low, presumably because stochastic drift was then the driving force. When selection was weak, asymmetric migration between the large and proximate smaller patches produced highly variable changes in the frequency of the locally adaptive allele in the smaller patches. Even in the large patches, where in the absence of drift, selection might be expected to act efficiently, the anticipated changes in adaptive alleles were only observed when dispersal ability was limited and barrier gap permeability was low. The interplay between local selection and asymmetric migration that we found in our smaller patches demonstrates what we might expect to see as species' ranges shift due to climate change.

Conclusions

Contemporary ecological theory reflects a scientific world-view heavily influenced by mathematics and simulation models. But these useful abstractions only have practical value when equations can be formulated and solved, or source code can be designed and executed; and as is true with science in general, ecologists have made use of simplifying assumptions in order to keep model development tractable. The cost of these simplifications has been that our theory can lack rigorous grounding in the very biological detail that we know governs species' interactions with their environments, and with each other. Simplifying assumptions are a practical necessity, but we should resist becoming too comfortable with them.

Our study used a two-pronged approach to examine these issues. First, we illustrated how advanced software tools (HexSim, in our case) allow researchers to explore complex biological mechanisms while retaining species-landscape and intraspecific interactions. Second, we examined specific simplifying assumptions that have shaped the development of theories in landscape genetics, population genetics, conservation biology, and evolutionary ecology. For each discipline, we used our hypothetical model system to illustrate how renewed attention to pattern and process can highlight the limits of existing theory. Our objective is not to find fault in past work, but rather to improve our models by challenging the assumptions upon which they have been constructed. To be sure, eco-evo theories of the future will extend existing

models, not discard them. And the next generation of eco-evo theory will better explain patterns and processes when landscapes are spatially and temporally dynamic, species' life histories and genetics are complex and interacting, and when the details matter.

The simulations described here are, by design, simple and straightforward. But they illustrate fundamental biological forces, track familiar observable responses, and address pressing challenges in the fields of landscape genetics, population genetics, conservation biology, and evolutionary ecology. We also expect our methods to have applications in the fields of phylogenetics, phylogeography, medical science, and evolutionary theory. For example, phylogeneticists might simulate past species' range shifts, model incomplete lineage sorting, or ask how linkage affects coalescent metrics. Phylogeographers may explore the stability of admixture zones over time, or improve the demarcation of evolutionary significant units. More generally, future applications of our methods will benefit from the HexSim modeling platform's ability to incorporate dynamic geographically realistic landscapes, and to simulate real species, interactions, and disturbance regimes.

While our simulations included only simple microsatellite-like genotypes, we anticipate future projects will investigate how mode of inheritance, linkage between alleles, initial geographic distributions of allele frequencies, and multiple mutation regimes (e.g., stepwise, infinite alleles, or two-phase models) interact with landscape spatial pattern and movement. Conservation geneticists may simulate mutation rates based on exposure to spatially-distributed chemical mutagens encountered during dispersal and mitigated by selection against deleterious alleles. Similarly, rates and patterns of gene-flow may be investigated within the context of nuanced, biologically-realistic movement behaviors, including rare long-distance dispersal events, site fidelity and memory, attraction and avoidance, sex-specific behaviors, to name a few. HexSim's design makes all of these simulation strategies possible, and more, while still being an ideal platform for simple models like the one explored in this study. Our methodology can also be extended to capture complex intermediate relationships between genotype, phenotype, and fitness, thus opening the door to evolutionary modeling at the level of genes, phenotypic heritable traits, populations, or even communities.

Acknowledgements

Allen Brookes developed the source code for HexSim and has been an invaluable team member. David Beck at the University of Washington eScience Institute aided in data management and processing. The information in this document has been subjected to review by the US EPA Center for Public Health and Environmental Assessment, and approved for publication.

References

1. Martens EA, Kostadinov R, Maley CC, Hallatschek O. Spatial structure increases the waiting time for cancer. New J Phys. 2011; 13 (11): 115014. https://doi.org/10.1088/1367-2630/13/11/115014

2. Walther GR, Post E, Convey P, Menzel A, Parmesan C, Beebee TCJ, Fromentin JM, Hoegh-Guldberg O, Bairlein F. Ecological responses to recent climate change. Nature. 2002; 416 (6879): 389–95. https://doi.org/10.1038/416389a

3. Waldvogel AM, Feldmeyer B, Rolshausen G, Exposito-Alonso M, Rellstab C, Kofler R, Mock T, Schmid K, Schmitt I, Bataillon T, Savolainen O, Bergland A, Flatt T, Guillaume F, Pfenninger M. Evolutionary genomics can improve prediction of species' responses to climate change. Evol Lett. 2020; 4: 4-18. https://doi.org/10.1002/evl3.154

4. Kinnison MT, Hendry AP. The pace of modern life II: from rates of contemporary microevolution to pattern and process. In Microevolution rate, pattern, process, edited by A. P. Hendry and M. T. Kinnison. Contemporary Issues in Genetics and Evolution 8. Springer Netherlands. 2001; 145–64. https://doi.org/10.1007/978-94-010-0585-2_10

5. Thompson JN. Rapid Evolution as an ecological process. Trends Ecol Evol. 1998; 13 (8): 329–32. doi:10.1016/S0169-5347(98)01378-0. https://doi.org/10.1016/S0169-5347(98)01378-0

6. Sinervo B, Svensson E, Comendant T. Density cycles and an offspring quantity and quality game driven by natural selection. Nature. 2000; 406 (6799): 985–88. https://doi.org/10.1038/35023149

7. Yoshida T, Jones LE, Ellner SP, Fussmann GF, Hairston NG. Rapid evolution drives ecological dynamics in a predator–prey system. Nature. 2003; 424 (6946): 303–6. https://doi.org/10.1038/nature01767

8. Hairston NG, Ellner SP, Geber MA, Yoshida T, and Fox JA. Rapid evolution and the convergence of ecological and evolutionary time. Ecol Lett. 2005; 8 (10): 1114–27. https://doi.org/10.1111/j.1461-0248.2005.00812.x

9. Pelletier F, Clutton-Brock T, Pemberton J, Tuljapurkar S, Coulson T. The evolutionary demography of ecological change: linking trait variation and population growth. Science. 2007; 315 (5818): 1571–74. https://doi.org/10.1126/science.1139024

10. Reznick DN, Bassar RD, Handelsman CA, Ghalambor CK, Arendt J, Coulson T, Potter T, Ruell EW, Torres-Dowdall J, Bentzen P, Travis J. Eco-evolutionary feedbacks predict the time course of rapid life-history evolution. Am Nat. 2019; 194(5): 671-692. https://doi.org/10.1086/705380

11. Oke KB, Cunningham CJ, Westley PAH et al. Recent declines in salmon body size impact ecosystems and fisheries. Nat Commun. 2020; 11, 4155. https://doi.org/10.1038/s41467-020-17726-z

12. Bassar RD, Coulson T, Travis J, Reznick DN. Towards a more precise – and accurate – view of eco-evolution. Ecol Lett. 2021; 24(4): 623-625. https://doi.org/10.1111/ele.13712

13. Zeller KA, Lewsion R, Fletcher RJ, Tulbure MG, Jennings MK. Understanding the importance of dynamic landscape connectivity. Land. 2020; 9(9), 303. https://doi.org/10.3390/land9090303

14. Schumaker NH, Brookes A. HexSim: a modeling environment for ecology and conservation. Landsc Ecol. 2018; 33:197-211. https://doi.org/10.1007/s10980-017-0605-9

15. Balkenhol N, Waits LP, Dezzani RJ. Statistical approaches in landscape genetics: an evaluation of methods for linking landscape and genetic data. Ecography. 2009; 32 (5): 818–30. https://doi.org/10.1111/j.1600-0587.2009.05807.x

16. Landguth EL, Forester BR, Eckert AJ, Shirk AJ, Menon M, Whipple A, Day CC, Cushman SA. Modelling multilocus selection in an individual-based, spatially-explicit landscape genetics framework. Mol Ecol. Resour. 2020; 20(2), 605– 615. https://doi.org/10.1111/1755-0998.13121

17. Hoban S, Bertorelle G, Gaggiotti OE. Computer simulations: tools for population and evolutionary genetics. Nat Rev Genet. 2012; 13 (2): 110–22. https://doi.org/10.1038/nrg3130

18. Bradburd GS, Ralph PL. Spatial population genetics: it's about time. Annu Rev Ecol Evol Syst. 2019; 50: 427-449. https://doi.org/10.1146/annurev-ecolsys-110316-022659

19. Lande R. genetics and demography in biological conservation. Science. 1988; 241 (4872): 1455–60. https://doi.org/10.1126/science.3420403

20. Frankham R. Genetics and extinction. Biol Conserv. 2005; 126 (2): 131–40. https://doi.org/10.1016/j.biocon.2005.05.002

21. Akçakaya, HR, Root W. RAMAS GIS: linking spatial data with population viability analysis. Applied Biomathematics. 2005. Setauket, New York.

22. Lacy RC, Pollak JP. Vortex: A stochastic simulation of the extinction process. Version 10.5.5. 2021. Chicago Zoological Society, Brookfield, Illinois, USA.

23. Pelletier F, Garant D, Hendry AP. Eco-evolutionary dynamics. Philosophical Transactions of the Royal Society B: Biological Sciences. 2009; 364(1523), 1483-1489. https://doi.org/10.1098/rstb.2009.0027

24. Hendry AP. Eco-evolutionary dynamics. Princeton university press. 2016. 416 pages. ISBN: 9780691204178

25. Schoener TW. The newest synthesis: understanding the interplay of evolutionary and ecological dynamics. Science. 2011; 331(6016), 426-429. https://doi.org/10.1126/science.1193954

26. Legrand D, Cote J, Fronhofer EA, Holt RD, Ronce O, Schtickzelle N, Travis JM, Clobert J. Eco-evolutionary dynamics in fragmented landscapes. Ecography. 2017; 40(1), 9-25. https://doi.org/10.1111/ecog.02537

27. Pritchard JK, Stephens M, Donnelly P. Inference of population structure Using multilocus genotype data. Genetics. 2000; 155 (2): 945–59. https://doi.org/10.1093/genetics/155.2.945

28. Hamilton WD. The moulding of senescence by natural selection. J Theor. Biol. 1966; 12 (1): 12–45. https://doi.org/10.1016/0022-5193(66)90184-6

29. Wright S. Isolation by distance. Genetics. 1943; 28 (2): 114–38. https://doi.org/10.1093/genetics/28.2.114

30. Miller MP. Alleles In Space (AIS): Computer software for the joint analysis of interindividual spatial and genetic information. J Hered. 2005; 96 (6): 722–24. https://doi.org/10.1093/jhered/esi119

31. Paradis E, Claude J, Strimmer K. APE: Analyses of phylogenetics and evolution in R language. Bioinformatics. 2004; 20 (2): 289–90. https://doi.org/10.1093/bioinformatics/btg412

32. R Core Team. R: a language and environment for statistical computing. 2013. Vienna, Austria: R Foundation for Statistical Computing. https://www.R-project.org/

33. Pritchard JK, Wen X, Falush D. Documentation for structure software: Version 2.3. 2010. https://web.stanford.edu/group/pritchardlab/structure.html

34. Evanno G, Regnaut S, and Goudet J. Detecting the number of clusters of individuals using the software Structure: a simulation study. Mol Ecol. 2005; 14 (8): 2611–20. https://doi.org/10.1111/j.1365-294X.2005.02553.x

35. Keenan K, McGinnity P, Cross TF, Crozier WW, Prodöhl PA. diveRsity: An R package for the estimation and exploration of population genetics parameters and their associated errors. Methods Ecol Evol. 2013; 4 (8): 782–88. https://doi.org/10.1111/2041-210X.12067

36. Cushman SA & Landguth EL. Spurious correlations and inference in landscape genetics. Mol Ecol. 2010; 19(17), 3592-3602. https://doi.org/10.1111/j.1365-294X.2010.04656.x

37. Zeller KA, Creech TG, Millette KL, Crowhurst RS, Long RA, Wagner HH, Balkenhol N, Landguth EL. Using simulations to evaluate Mantel-based methods for assessing landscape resistance to gene flow. Ecol Evol. 2016; 6: 4115-4128. https://doi.org/10.1002/ece3.2154

38. Waples RS, Gaggiotti O. What is a population? An empirical evaluation of some genetic methods for identifying the number of gene pools and their degree of connectivity. Mol Ecol. 2006; 15 (6): 1419–39. https://doi.org/10.1111/j.1365-294X.2006.02890.x

39. Lande R, Barrowclough GF. Effective population size, genetic variation, and their use in population management. Pages 87-123 in M. E. Soule, editor. Viable populations for conservation. Cambridge University Press, Cambridge, England. 1987.

Figure 1

Click here to access/download;Figure;Figure 1.png

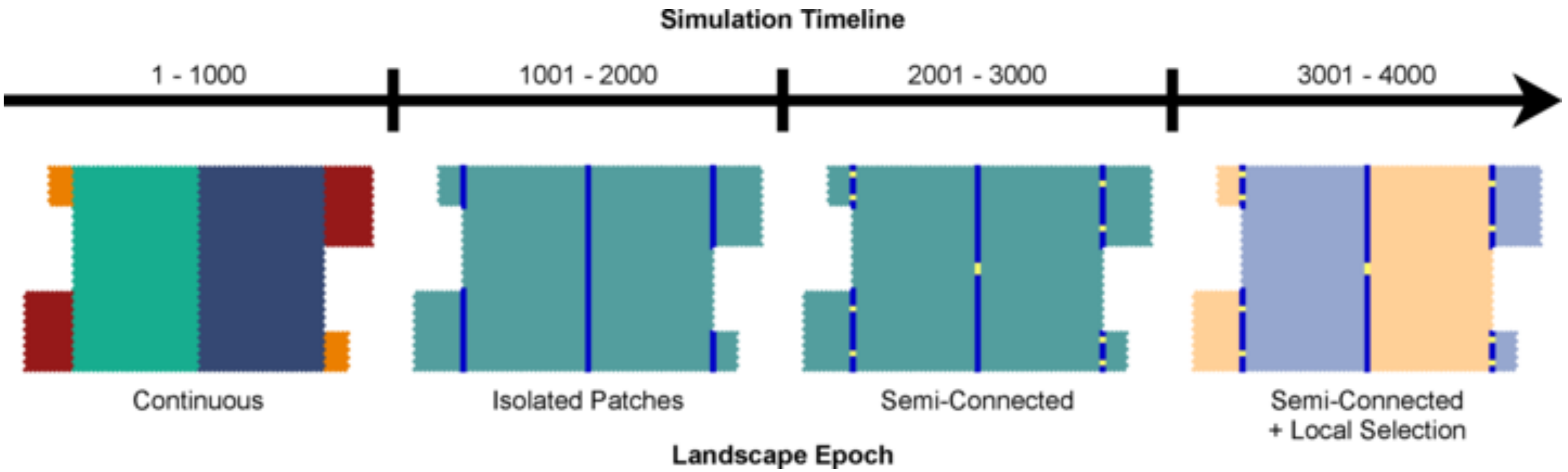

Figure 2

Click here to access/download;Figure;Figure 2.png

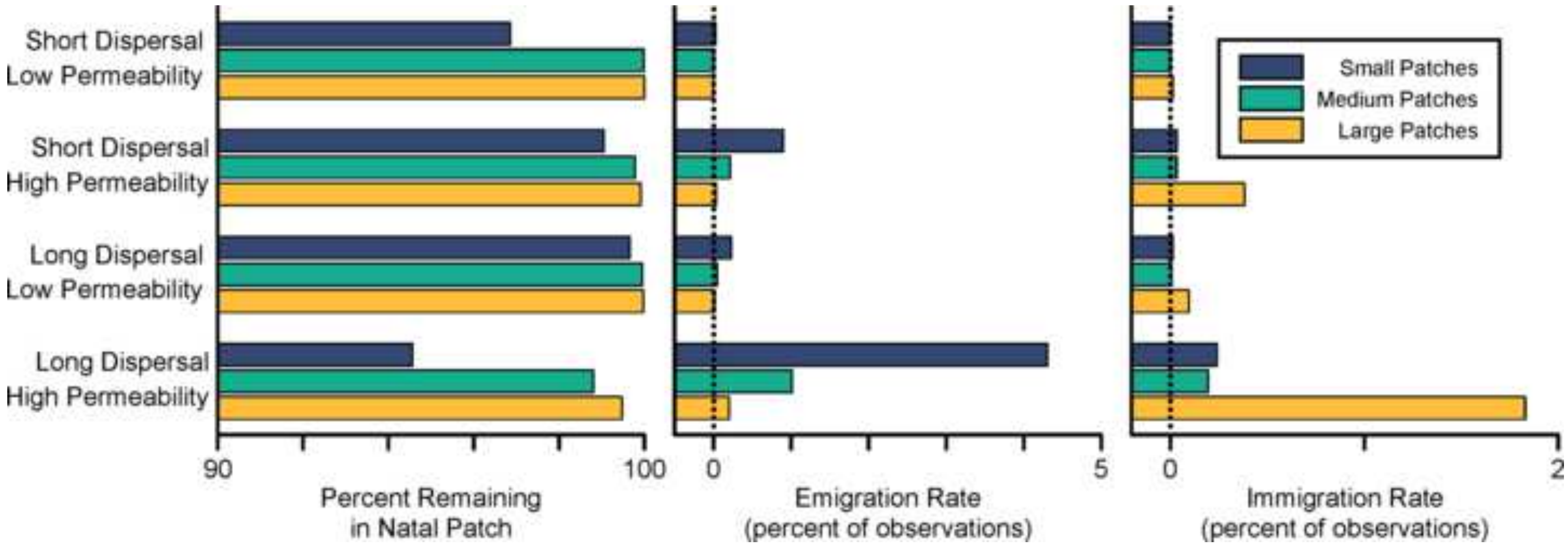

Figure 3

Click here to access/download;Figure;Figure 3.png

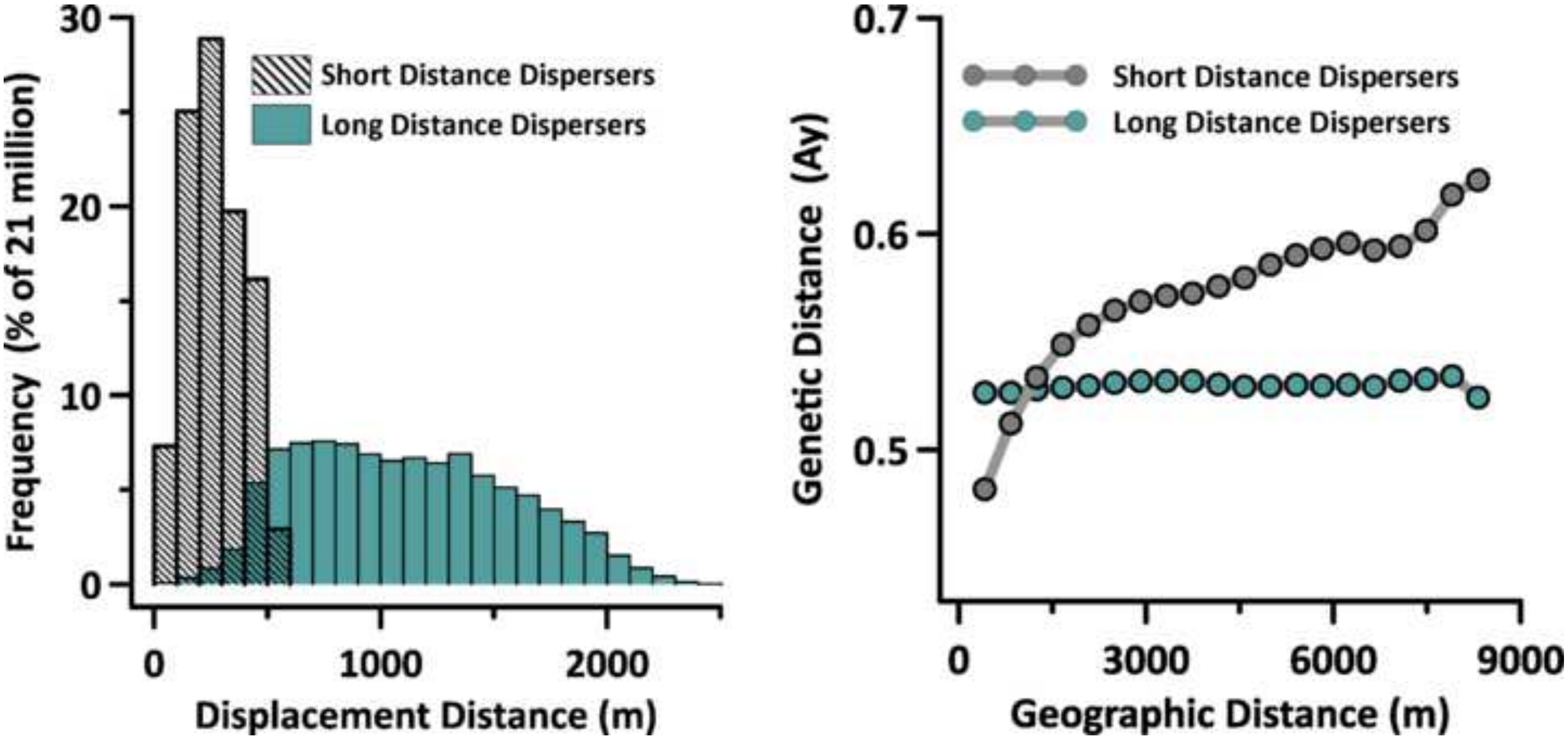

Figure 4

Click here to access/download;Figure;Figure 4.png

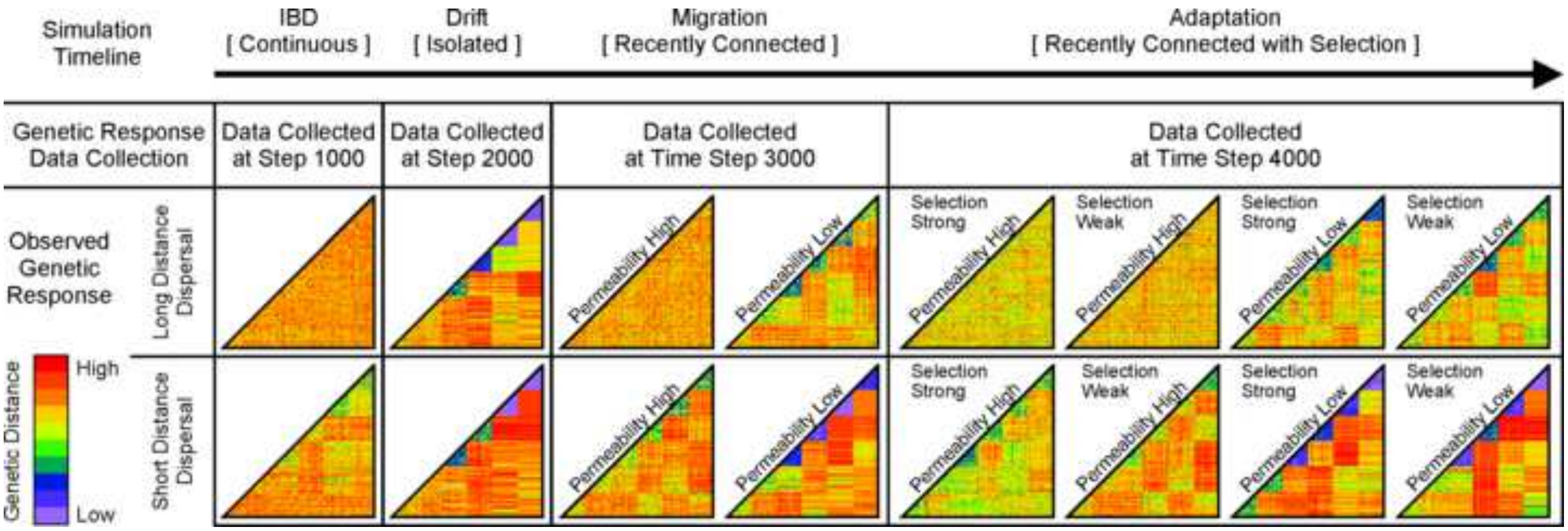

Figure 5

Click here to access/download;Figure;Figure 5.png

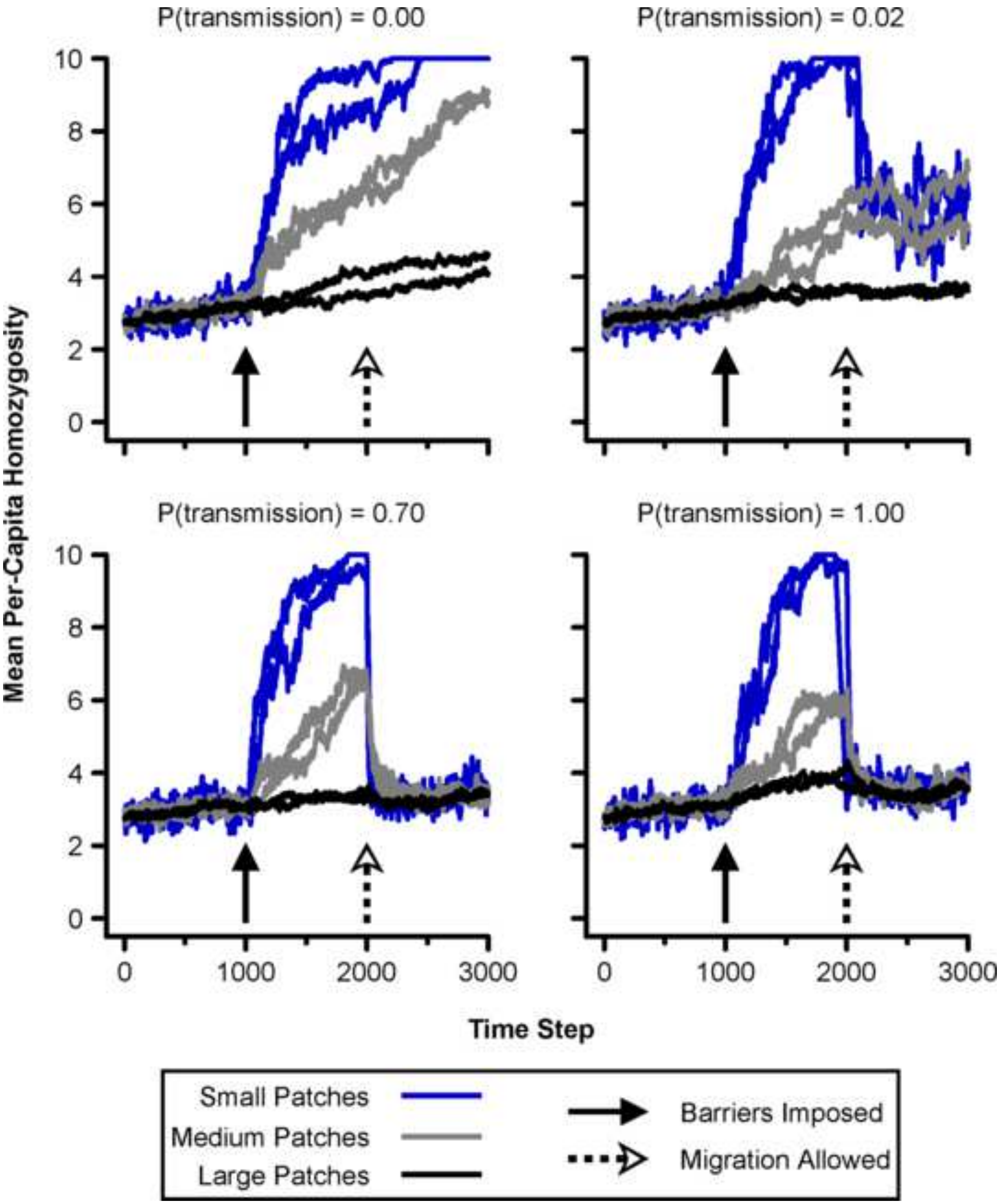

Figure 6

Click here to access/download;Figure;Figure 6.png

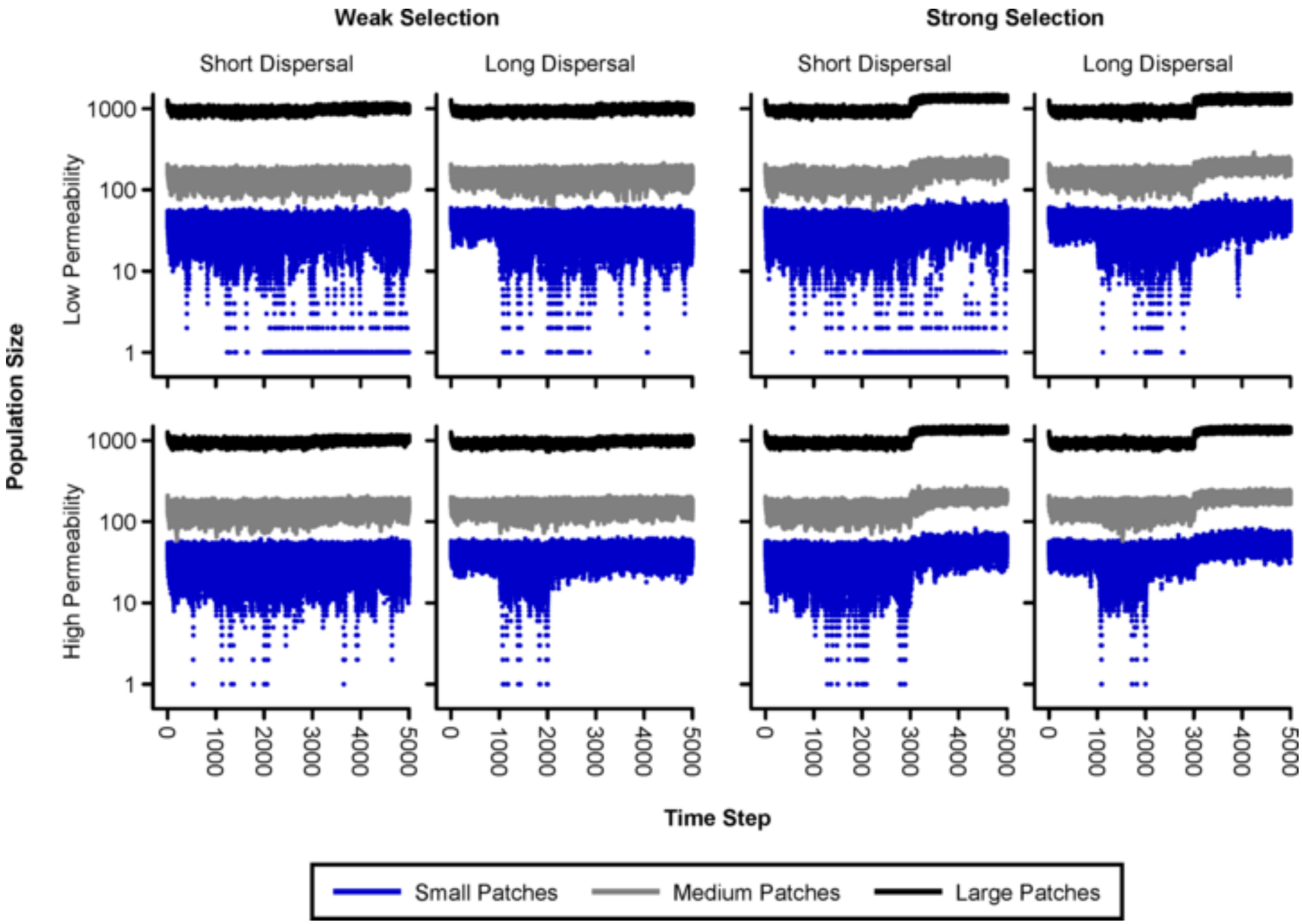

Revised Manuscript with Track Changes

Adding pattern and process to eco-evo theory and applications

Jennifer M. White [1]
Nathan H. Schumaker [2]*
Rachel Y. Chock [3]
Sydney M. Watkins [4]

[1] Center for Conservation Biology, Department of Biology, University of Washington, Seattle, Washington, United States of America

[2] U.S. Environmental Protection Agency, Pacific Ecological Systems Division, Corvallis, Oregon, United States of America

[3] San Diego Zoo Wildlife Alliance, Conservation Science, Escondido, California, United States of America

[4] Oak Ridge Institute for Science and Education, Oak Ridge, Tennessee, United States of America

*Corresponding author:
E-mail: nathan.schumaker@gmail.com (NHS)

Abstract

Eco-evolutionary dynamics result when interacting biological forces simultaneously produce demographic and genetic population responses. Eco-evolutionary simulators traditionally manage complexity by minimizing the influence of spatial pattern on process. However, such simplifications can limit their utility in real-world applications. We present a novel simulation modeling approach for investigating eco-evolutionary dynamics, centered on the driving role of landscape pattern. Our spatially-explicit, individual-based mechanistic simulation approach overcomes existing methodological challenges, generates new insights, and paves the way for future investigations in four focal disciplines: Landscape Genetics, Population Genetics, Conservation Biology, and Evolutionary Ecology. We developed a simple individual-based model to illustrate how spatial structure drives eco-evo dynamics. By making minor changes to our landscape's structure, we simulated continuous, isolated, and semi-connected landscapes, and simultaneously tested several classical assumptions of the focal disciplines. Our results exhibit expected patterns of isolation, drift, and extinction. By imposing landscape change on otherwise functionally-static eco-evolutionary models, we altered key emergent properties such as gene-flow and adaptive selection. We observed demo-genetic responses to these landscape manipulations, including changes in population size, probability of extinction, and allele frequencies. Our model also demonstrated how demo-genetic traits, including generation time and migration rate, can arise from a mechanistic model, rather than being specified *a priori*. We identify simplifying assumptions common to four focal disciplines, and illustrate how new insights might be developed in eco-evolutionary theory and applications by better linking biological processes to landscape patterns that we know influence them, but that have understandably been left out of many past modeling studies.

Introduction

Eco-evolutionary dynamics are shaped by spatial patterns and mediated by movement. The influence of spatial pattern on eco-evolutionary (hereafter "eco-evo") dynamics spans spatio-temporal scales and scientific disciplines, from cancer development within populations of somatic cells [1] to ecosystem responses to climate change (e.g., [2, 3]). With advances in molecular genetics, the time scales between evolutionary and ecological forces have been shown to be sufficiently coincident [4] and feedbacks from rapid-evolution impinging on ecological processes have been directly observed in an increasing number of natural systems [5-11]. For example, evolutionary geneticists follow the evolution of heritable traits responding to spatially heterogeneous selection pressures, population geneticists examine changes in allele frequencies influenced by spatially-restricted gene-flow, phylogeographers investigate coalescent timing across glaciations, and conservation biologists explore links between genetic diversity and resilience in spatially isolated populations. Despite the inherently spatial nature of these processes, the dynamics of landscape structure are rarely considered. But landscape dynamics may be critical for understanding reciprocal feedbacks between ecology and evolution [12], and for informing management applications [13].

Here, we illustrate how spatial structure drives eco-evo dynamics within four focal disciplines: Landscape Genetics, Population Genetics, Conservation Biology, and Evolutionary Ecology. We employ computer simulations to illustrate these dynamics using a novel modeling environment called HexSim [14] in which both biological forces and observable demographic and genetic responses emerge mechanistically from changes to landscape structure. HexSim life history processes are directly linked to static or dynamic landscape maps, and multiple spatial drivers can simultaneously influence different parts of the same simulation. Further, movement responses to landscape structure are not constrained by a reliance on resistance surfaces, patch-mosaic structures, stepping stones, or the use of graph-theoretic networks – all simplifications that are frequently employed to speed model development, but at a cost to biological realism. Lastly, HexSim eco-evo processes are mechanistically connected using a highly flexible system of demographic and genetic (hereafter "demo-genetic") life history traits.

While more than 60 publications have resulted from HexSim-based studies (www.hexsim.net), adoption and application of the model's genetics toolkit has lagged. Below we describe how increasing attention to spatial and demographic details, and the further integration of ecological and evolutionary processes, will likely contribute to the focal disciplines mentioned above. Both the HexSim application and the simulation models described here are available at www.hexsim.net.

Landscape Genetics

This relatively new field endeavors to describe how landscape pattern influences gene-flow. A standard approach is to compare inter-individual genetic distance to metrics of landscape structure, often quantified as a cost-distance across a resistance surface. Available landscape genetics modeling platforms have had limited spatial, demographic, or behavioral sophistication [15], though some such constraints are being actively addressed by leaders in the field (e.g., [16]). Models that are unable to incorporate mechanisms underlying species-landscape interactions have a limited ability to simulate complex movements and resulting gene-flow. Additionally, most platforms cannot simultaneously simulate multiple interacting eco-evo drivers of gene-flow, such as local adaptation along with source-sink demographics [17].

Population Genetics

Investigators study the causes and consequences of population genetic structure. For simplicity, complex biological forces such as selection and mutation rates are often assumed to be spatially constant. Additionally, in classic population genetic models, spatial complexity is reduced to discrete populations, and migration processes are characterized by a single univariate input parameter, '*m*'. These simplifications have facilitated the generation of a wealth of theory; but, a lack of biological realism and spatial complexity have precluded the application of this theory to systems in which space is heterogeneous and dynamic (e.g., where landscape structure influences population size, density, dispersal flux, or local adaptation), or when gene-flow is governed by complex movement and mating behavior (e.g., when dispersal rates and distances are unequal, or mate-choice is non-random; see [18] for review).

Conservation Biology

This broadly defined discipline frequently involves the exploration of forces affecting population viability. Landscape pattern drives population viability through its influence on ecological and evolutionary processes. The relative importance of demography versus genetics has been actively debated in the conservation literature [19, 20]. Some forecasting tools can simulate either inbreeding rates or landscape use, and incorporate them into probabilities of extinction (e.g., *RAMAS GIS* [21] or *Vortex* [22]), but existing models simplify the linkages between ecological and evolutionary processes, and these shortcomings limit the utility of their forecasts. For example, a platform capable of simulating complex interacting demographic and genetic traits is necessary to forecast how landscape changes will alter a population's size and distribution (eco), while simultaneously impacting its mate-finding or natural selection (evo).

Evolutionary Ecology

Researchers explore interactions and feedback between ecological and evolutionary drivers that affect demographic and genetic traits. Landscape patterns shape myriad mechanisms through which biological forces influence population and community demo-genetic traits. Renewed interest in these processes (e.g., [23, 24]) has underscored the need for biologically sophisticated, mechanistic simulation platforms capable of explicitly modeling dynamic eco-evo feedback in a spatially-realistic setting. Simply modeling how environmental change impacts heritable traits is only half of the story; our long-term goals must also include asking how these emergent genetic traits connect back to ecological dynamics [25, 26].

Below, we describe a relatively simple theoretical model designed to illustrate how the explicit inclusion of detailed spatial patterns and biologically realistic processes might facilitate the development of new eco-evo theory and applications. We demonstrate model relevance by highlighting results that (a) illustrate *a priori* expectations of a core eco-evo dynamic fundamental to a discipline, and (b) illustrate how additional biological and spatial realism might contribute to new theory and improve our confidence in modeling applications (Table 1). Our primary goals include both evaluating and articulating the benefits derived from adding spatial structure to genetic models, and illustrating the mechanics involved in doing this well.

While the incorporation of space can substantially enhance realism and defensibility, it also complicates models, especially those which are already mechanistically-rich (e.g., that integrate demography, movement, and gene flow). In pursuit of the former goal, we trade away elements of realism, in the form of landscape structure and movement behavior, for mechanistic clarity. Readers evaluating our assertions will benefit from the simplicity of our simulations, as the number of drivers and responses they must track is limited. In regards to the latter goal, our work constitutes an important extension to the existing descriptions and applications of HexSim [14]. Readers interested in adopting this software will benefit from the case studies discussed here, as they provide detail on HexSim genetics that is unavailable in previous publications.

Table 1. How spatially structured eco-evolutionary models might contribute to four focal disciplines.

Discipline	Research Questions	Expected Outcomes	Anticipated Contributions
**Landscape Genetics**	How is gene-flow controlled by the landscape?	Isolation by Distance is a function of dispersal behavior.	Replacing resistance surfaces and cost-distance matrices with measures of genetic distance arising from eco-evo processes and species-landscape interactions.
**Population Genetics**	How is genetic structure controlled by the landscape?	Genetic structure is influenced by isolation by distance and demography.	Allowing migration rates between populations to emerge mechanistically from the interplay between dispersal behavior and landscape structure.
**Conservation Biology**	How are inbreeding and population viability controlled by the landscape?	Homozygosity and stochastic extinction are consequences of demographic processes.	Ensuring that forecasts of genetic degradation are driven by spatially-realistic movement models and incorporate pre-existing genetic structure and diversity.
**Evolutionary Ecology**	How are feedbacks between ecological and heritable traits controlled by the landscape?	Population demo-genetic traits are an ecological response to the evolutionary force of selection.	Creating eco-evo feedback loops between local selection for specific alleles and counteracting asymmetric migration stemming from source-sink dynamics in heterogeneous landscapes.

Methods

Our study was built around a simple heuristic simulation constructed within the HexSim modeling environment [14]. HexSim is a user-friendly, spatially-explicit, individual-based, demo-genetic modeling environment. We modeled a single species, and parameterized its life history, demographics, genetic traits, and the interactions between habitat type and genetics (described below). We created a minimally-complex landscape structure that varied episodically throughout our simulations. Each epoch (1,000 simulation time steps) included

unique barriers, barrier gaps, or habitat types. We varied behavior (dispersal distance), strength of selection, and barrier gap permeability for a total of eight treatment combinations, and tracked individual genotypes, per-capita homozygosity, per-patch population size, and the number of dispersers moving between the patches in each treatment. Through the use of this single model, we were able to probe expected outcomes of the four focal disciplines, and test the anticipated behaviors of this eco-evo modeling approach.

Landscape Structure

Our landscape was composed of six adjacent habitat patches (two small, two intermediate, two large) built up from multiple hexagonal cells of uniform quality (Fig 1). Two large adjoining patches (1326 hexagons each) lie at the landscape center. Two medium-sized patches (200 hexagons each) and two small patches (50 hexagons each) abut the large patches, but not each other. Patch dimensions expressed as columns × rows, were 26 × 51, 10 × 20, and 5 × 10. Collectively, the six patches were assembled from 3152 individual hexagons. Our simulated individuals were never allowed to enter the surrounding non-habitat matrix. Movement barriers were used, at times, to isolate the patches from each other. At other times, small semi-permeable openings in the barriers allowed limited dispersal between neighboring patches. The total size of the barrier gaps between adjacent patches was identical, ensuring individuals had a uniform potential for crossing all patch interfaces.

Fig 1. The progression of landscape epochs over 4000 simulation time steps. Movement barriers (shown as dark blue lines) and landscape edges are reflective. The six patches are distinguished by color within the *Continuous* epoch. Yellow barrier gaps, introduced at time step 2001, are of the same total length for each barrier. At time step 3001, the landscape is assigned two distinct habitat types (light blue vs. orange) that confer genotype-specific adaptive advantage to juvenile survival.

Individual Ecological Characteristics

Our simulations ran for a series of time steps, each corresponding to one year, with a sequence of life history events and species-landscape interactions performed at each step. Note that we

subsequently use the terms "time step" and "year" synonymously. We favor "time step" when describing model mechanics, and "year" when interpreting simulation output. In brief, the life cycle consisted of (a) resource acquisition, (b) pair formation, (c) reproduction, (d) juvenile dispersal, and (e) survival. The individuals making up our population included both sexes and two age classes (juvenile and adult), and our simulations began with the landscape being saturated with adults (3152 individuals spread uniformly across all patch hexagons). Adult females reproduced only if they could pair exclusively with an adult male located nearby (neither females or males had multiple mates). Individuals were assigned normally distributed reproductive rates, with mean values based on their resource allocation, which introduced a density-dependent feedback that was further modified by the requirement for pair-formation. Individuals acquired resources from a roughly-circular neighborhood of 37 hexagonal cells, and resources were shared equally by all individuals attempting to utilize them (scramble competition). Juveniles dispersed from their natal site in the year of their birth, and transitioned to adults at the start of the subsequent year. Adults did not move. Dispersal path lengths were drawn from one of two uniform distributions, either “short” (1-5 hexagon steps) or “long” (5-25 hexagon steps). Dispersal autocorrelation was set at 75, on a scale of 0 (completely random) to 100 (perfectly linear). Individual dispersers took a series of steps from hexagon to adjacent hexagon, and stopped when their path length had been reached. Yearly survival probability was based on stage class (juvenile = 0.500, adult = 0.885), but individuals with less than 20% of their resource goal were assigned an additional 10% probability of mortality. Our simulations included a period during which survival decisions were also based on genetic adaptation. In these cases, emergent mortality rates became jointly determined by stage class, location, and genotype (see below).

Individual Evolutionary Characteristics

Our simulated individuals were diploid with ten loci and zero linkage between loci. Each locus was assigned five alleles labeled A1-A5. The starting population’s allele assignment was governed by locus-specific initial allele frequencies (Table 2). Initial allele frequencies were not spatially stratified, and we did not simulate mutation. Offspring genotypes were assembled by

drawing a single allele from each parent at each locus. Individuals possessed five purely neutral loci (L1-L5), and five loci (L6-L10) containing at least one allele capable of conferring a fitness advantage. These locally adaptive alleles imparted a survival benefit to juveniles when they were in the habitat type to which they were genetically pre-adapted. The survival benefit (*S*) per adaptive allele was either strong (*S* = 0.10) or weak (*S* = 0.01). Modeled this way, selection was a predictable process because genotype dictated the mean juvenile survival probability. Selection was initiated at time step 3000. All ten loci were utilized for all genetic analyses described below, with the exception of adaptation, for which only L6-L10 were employed.

Table 2. Initial allele frequencies for 10-locus genotypes.

Locus	Allele	Initial Allele Frequency	Local Adaptation
1	1-5	0.20, 0.20, 0.20, 0.20, 0.20	Neutral
2	1-5	0.30, 0.25, 0.20, 0.15, 0.10	Neutral
3	1-5	0.10, 0.15, 0.20, 0.25, 0.30	Neutral
4	1-5	0.01, 0.04, 0.15, 0.30, 0.50	Neutral
5	1-5	0.50, 0.30, 0.15, 0.04, 0.01	Neutral
6	1	0.20	Neutral
	2	0.20	Locally Adapted - Habitat Type A
	3	0.20	Neutral
	4	0.20	Locally Adapted - Habitat Type B
	5	0.20	Neutral
7	1	0.30	Neutral
	2	0.25	Neutral
	3	0.20	Neutral
	4	0.15	Neutral
	5	0.10	Locally Adapted - Habitat Type A
8	1	0.10	Neutral
	2	0.15	Neutral
	3	0.20	Neutral

	4	0.25	Neutral
	5	0.30	Locally Adapted - Habitat Type B
9	1	0.01	Locally Adapted - Habitat Type A
	2	0.04	Neutral
	3	0.15	Neutral
	4	0.30	Neutral
	5	0.50	Neutral
10	1	0.50	Locally Adapted - Habitat Type B
	2	0.30	Neutral
	3	0.15	Neutral
	4	0.04	Neutral
	5	0.01	Neutral

Progression of Landscape Change

All of our landscapes were binary, consisting of habitat patches embedded within a non-habitat matrix. Our initial landscape was continuous and free from movement barriers. This landscape, hereafter referred to as *Continuous*, persisted for the first epoch of 1000 simulation time steps (Fig 1). We anticipated that Isolation by Distance (IBD) would be the predominant evolutionary force in this landscape. To simulate the effect of drift alone, we then imposed absolute movement barriers that isolated subpopulations into six separate landscape patches (two each, of three different sizes) for the subsequent 1000 simulation time steps. We refer to this epoch as *Isolated Patches*.

Following patch isolation, we created small gaps in the movement barriers, allowing infrequent migration between the previously isolated sub-populations. Barrier gaps varied in permeability (high = 0.70 transmission probability per encounter, low = 0.02 transmission probability per encounter), affecting the likelihood that individuals would cross the barrier when they encountered a gap during dispersal. We refer to this third epoch of 1000 time steps as *Semi-Connected*. During the final 1000 time steps, the patches were each assigned one of two

"habitat types" that conferred increased fitness to juveniles with specific genotypes. We refer to this landscape as *Semi-Connected with Local Selection*.

Treatments and Observable Responses

Individual dispersal behavior (long vs. short), strength of selection (strong vs. weak), and barrier gap permeability (low vs. high) together formed eight treatment combinations. Each treatment was repeated in ten replicates. For each treatment, we tracked individual genotypes, per-capita homozygosity, per-patch population size, and the number of dispersers moving between the patches. We did not track individual pedigree information. Upon completion of the simulations, we used HexSim's report generator to create files suitable for input to the genetic software package STRUCTURE [27], which we subsequently used for some of our analyses.

Adult females in our model could reproduce each time step until their death, thereby producing overlapping generations. We measured generation time as the observed average age of reproducing females [28]. Migration rates between patches were measured during the *Semi-Connected* epoch. To qualify as a migrant, individuals had to cross through a barrier gap and subsequently reproduce somewhere other than their natal patch. Our migration data thus intentionally excluded non-breeders and individuals that reproduced in their natal patch after making a temporary excursion elsewhere.

Landscape Genetics

Isolation by distance (IBD) produced by dispersal limitations [29] is a core concept in landscape genetics. We used results from the *Continuous* epoch to illustrate the degree of IBD for our short and long-distance dispersal treatment groups. We constructed dispersal kernels by plotting the frequency of observed dispersal distances for all individuals in all replicates, from time step 1 to 1000. We visually assessed the degree of IBD using correlograms generated by the application *Alleles in Space* [30], where the average genetic distance between individuals is plotted for varying distance classes. We also plotted the frequency of observations for each distance class, to ensure that the inter-individual genetic distances were not due to underlying distribution of individuals on the landscape.

We calculated inter-individual genetic-distances resulting cumulatively from each landscape history. For ease of illustration, we subset our results as follows: for each treatment group, we randomly selected a single replicate simulation. From the selected replicate, we extracted genotype reports from the last time step of each of the four landscape epochs. From those reports, we randomly selected 25 individuals from each of the six patch locations. For those 150 individuals, we calculated a simple metric of pairwise genetic distance (number loci for which alleles differ between individuals / total number loci) using the "*ape"* R (v3.2.2) package "*dist.gene"* function with the "*percentage"* method [31, 32]. We visualized the 150 x 150 pairwise genetic distances as triangular matrices.

Population Genetics

We used a Bayesian clustering method, implemented by the program STRUCTURE (v 2.3.4), to analyze population genetic structure [27]. At the end of each landscape epoch, we collected genotype information stratified by putative population (patch), and imported these data into STRUCTURE. Unequal sample sizes (small patches ≈ 25 individuals, large patches ≈ 1000) interfered with the assessment of the number of subpopulations and assignment probabilities. This known limitation of the software [33] was easily overcome by randomly drawing a fixed number of individuals (n=25) from each patch for analysis. In cases where there were fewer than 25 individuals extant in a given patch, genotypes were randomly selected for inclusion more than once until each patch had a sample of 25, for 150 total individuals.

We expected the number of unique genetic clusters (commonly referred to as "K") to vary from 1 to 6 based on landscape structure. Therefore, following convention, we tested possible values of K ranging from 1 to 20. All STRUCTURE analyses were run with a burn-in period of 10,000 iterations, with an additional 10,000 analysis iterations. We performed 20 replicate trials for each possible K value using the default settings of the admixture model and correlated allele frequencies. The best supported *K* values were identified using two methods: 1) plotting the replicate average *Ln P(D|K)*, and visually determining the minimum *K* of the curve's asymptote [33], and 2) using Evanno's Δ*K* method [34]. When there were close ties between supported *K*

values from the competing methods, we considered them all for individual assignment analyses.

Conservation Biology

We calculated *Per-capita Homozygosity* as the percent of homozygous genotypes across all alleles and individuals within a given landscape patch. Large values of *Per-capita Homozygosity* could result from a few highly inbred individuals or from many slightly inbred individuals. We also examined the effect of patch isolation on allele frequencies from data collected at the ends of the *Continuous* and *Isolated Patches* epochs. We calculated three metrics of genetic degradation for each locus in each subpopulation using output from the "'*diveRsity"* R package "*divBasic"* function [35], as described below:

1) *Allelic richness* = the number of unique alleles per locus. All ten loci began with 5 alleles each.

2) *Allelic evenness* = $\frac{\sum p_i \ln(p_i)}{\ln(5)}$, where p_i is the frequency of the ith allele within each locus, and ln (5) is the maximum evenness in each locus, given that all were initiated with 5 alleles. The starting population had different initial allele frequencies corresponding to allelic evenness values of 1.0 = "Equal", 0.96 = "Unequal", and 0.72 = "Rare" (Table 2).

3) *Heterozygosity deficit* = $H_{obs} - H_{exp}$, where H_{obs} is the number of observed heterozygous genotypes and expected heterozygosity is calculated as: $H_{exp} = 1 - \sum_1^n (q_i)^2$, where n represents the number of alleles, and q_i is the frequency of the ith allele at a locus. Note that H_{exp} is calculated based on the number of extant alleles at a locus, and will fluctuate dramatically as alleles are lost from a population. Therefore, unlike *Allelic richness* or *Allelic evenness*, this metric has a shifting baseline.

For each metric, we calculated the mean and standard deviation across all replicates within the same patch size (small, medium, or large) and of the same initial allele frequency (equal, unequal, or rare) for each of the two time points of interest (at the end of the *Continuous* and *Isolated Patches* epochs).

Evolutionary Ecology

Population size was tracked for each patch across all time steps to facilitate measuring the response to adaptation. Additionally, we computed the change in frequency of adaptive alleles within each patch during the *Semi-Connected with Local Selection* epoch. L6 contained two adaptive alleles and 3 neutral, while L7-through L10 each contained one adaptive allele and 4 neutral. L6-A2, L7-A5 and L9-A1 were adaptive in half of the patches, while L6-A4, L8-A5 and L10-A1 were adaptive in the other half. Tracking allele frequencies in L6 allowed us to examine the effect of local adaptation on allele frequency in combination with asymmetric migration from adjacent patches, where the opposite allele was advantageous.

~~Computational Effort and Model Availability~~

~~All 80 simulations were run on a Dell PowerEdge R820 server. Individual model runs took roughly four hours of processing time to complete. Our simulations could likely be replicated at a higher speed on a modern desktop or laptop computer, and use of the server simply made it possible to run all simulations simultaneously. Both the HexSim application and the simulation models described here are available at www.hexsim.net.~~

Results

The age-class structure that arose from our model resulted in an average age of reproducing females of 8.7 years. Therefore, each landscape epoch of 1000 simulation time steps (Fig 1) spanned approximately 115 generations. During the *Semi-Connected* epoch, migration rate between adjacent patches varied with dispersal distance, barrier gap permeability, and patch size (Fig 2); but regardless of these values, the majority of individuals remained in their natal patch. As anticipated, we observed higher migration rates when individuals were assigned the long-distance dispersal behavior and the barrier gaps were highly permeable. Unequal perimeter-area ratios ensured that migration was asymmetric, with smaller patches generating proportionately greater numbers of emigrants than larger patches, and larger patches receiving proportionately higher numbers of immigrants than smaller patches.

Fig 2. Observed migration rates of long or short-dispersing individuals across barriers of varying permeability during the *Semi-Connected* epoch. Left: Percent of the population that is born and reproduces in the same patch. Middle: Emigration rates, sorted by size of sending patch. Right: Immigration rates, sorted by size of receiving patch.

Landscape Genetics

The short and long-distance dispersal behaviors measured during the *Continuous* landscape epoch produced notably different dispersal kernels (Fig 3 *left*). IBD was evident in the relationship between geographic distance and genetic distance when dispersal was short, but not when dispersal distances were long (Fig 3 *right*).

Fig 3. In a spatially-continuous landscape, the observed dispersal kernels reflected juvenile dispersal ability (left). Genetic IBD was evident when dispersal paths were short, but not when they became longer (right). These data were gathered at the end of the *Continuous* landscape epoch, after 115 generations of unobstructed movement.

We also interpret our series of landscape epochs as four nested hypothetical landscape histories: years 1-1000, years 1-2000, years 1-3000, and years 1-4000. Genetic distance matrices observed at the conclusion of each epoch illustrate the effectiveness of gene-flow at mixing subpopulations, given landscape history and dispersal behavior (Fig 4). Additionally, barrier gap permeability affects gene-flow in the *Semi-Connected* and *Semi-Connected with Local Selection* epochs, and selection strength affects gene-flow in the *Semi-Connected with Local Selection epoch*.

Fig 4. Modeling gene-flow for four landscape histories, beginning with IBD only, and building to a complex history of sequential epochs incorporating IBD, drift, migration, and finally, local selection. The resulting pairwise genetic distance matrices (colored triangles) illustrate the strength of gene-flow within a given landscape history, where increasing contrast in the geometric patterns visible within the matrices results from reduced gene-flow between landscape patches. Data collected at time step 4000 illustrate the interacting effects of gene-flow and local adaptation on genetic-distance. See Methods section for additional details.

Population Genetics

In the *Continuous* epoch, we know a single biological population was present on the landscape. But without the benefit, inherent in modeling, of knowing the true population dynamics, genetic analysis could easily lead to a conclusion that several distinct populations were present (Table 3). For example, neither Evanno's method or L(*K*) produced consistent results in this case. Short distance dispersal and the resulting IBD led to an inference of multiple genetic clusters under L(*K*), but a single genetic cluster under *E*. When dispersers moved longer distances, and IBD was therefore absent, the L(*K*) method produced the expected result, but Evanno's method did not (Table 3).

Table 3. Inferred number of genetic subpopulations *K* resulting from either the visual interpretation of likelihood probabilities *L(K)* or from Evanno's ΔK method *E*, as a function of dispersal distance and epoch. When a single method produced multiple equally-supported estimates of K, then all values were included.

	K	Continuous Epoch	Isolated Patches Epoch
**Long Distance Dispersal**	1	L(K)	
	2	E	
	5		L(K)
	6		L(K)
	8		E
**Short Distance Dispersal**	2	E	
	4	L(K)	
	5	L(K)	L(K)
	6		L(K)
	7		E

In the *Isolated Patches* epoch, there are 6 distinct populations isolated by absolute barriers. By the end of the epoch, 5-8 unique genetic clusters were identified by our genetic analyses, regardless of dispersal behavior (Table 3). Evanno's method (*E*) tended to produce inflated

estimates of *K* that exceeded the true number of 6 genetic subpopulations. The STRUCTURE plots (not shown) indicated clearly that the 4 smaller patches were each home to a single genetic cluster, made unique by drift. The two large patches housed either one or two genetic clusters, due to a weak effect of drift in these larger populations.

The population genetic structure produced by drift during the *Isolated Patches* epoch was mitigated by the limited inter-patch migration that characterized the subsequent *Semi-Connected* epoch (Table 4). The differing numbers of migrants per generation produced varying degrees of genetic mixing over the course of these 115 generations. The observed >15 migrants per generation resulting from long distance dispersal and high barrier gap permeability produced sufficient genetic mixing to return to panmixia, while the other treatments failed to do so. Long-distance dispersal with low barrier permeability resulted in <1 migrant per generation, and this migration rate was insufficient to return the population to panmixia within the 115 generations of the epoch. A return to panmixia by the end of the *Semi-Connected* epoch was not expected in simulations employing a short dispersal distance, and no such outcome was observed.

Table 4. The replicated average number of observed migrants per generation, presented as a function of dispersal distance and barrier gap permeability, at the end of the *Semi-Connected* epoch. In most cases, population genetic structure (change in possible *K*, evaluated from either the visual interpretation of likelihood probabilities *L(K)* (top) or from Evanno's ΔK method *E* (bottom)) declined from where it began in the prior *Isolated Patches* epoch.

	Low Barrier Gap Permeability		High Barrier Gap Permeability	
	**Migrants per Generation**	**Change in Possible K**	**Migrants per Generation**	**Change in Possible K**
**Long Distance Dispersal**	0.757	5 or 6 → 4	15.456	5 or 6 → 1
		8 → 6		8 → 2
**Short Distance Dispersal**	0.086	5 or 6 → 6	2.598	5 or 6 → 6
		7 → 2		7 → 2

Conservation Biology

In this analysis, which included only the *Continuous*, *Isolated Patches*, and *Semi-Connected* epochs, per-capita homozygosity varied dramatically with landscape epoch, and fluctuated more in the small patches than the large ones (Fig 5). While we observed little accumulation of homozygosity during the *Continuous* epoch, this changed when migration between patches was limited by dispersal barriers. During the *Isolated Patches* epoch, homozygosity increased rapidly, but this general trend was strongly influenced by patch size. Both patch size and barrier gap permeability affected the amount of "genetic rescue" resulting from migration during the *Semi-Connected* epoch.

Fig 5. Per-capita homozygosity across simulation time steps spanning the *Continuous*, *Isolated Patches*, and *Semi-Connected* landscape epochs, displayed by patch size. Our simulated low and high barrier crossing probabilities corresponded to P(transmission) values of 0.02 and 0.70, respectively. Six lines per plot result from two small, two medium, and two large patches.

Changes in allele frequencies observed during the *Continuous* and *Isolated Patches* epochs were affected by patch size, locus-specific initial allele frequencies, and dispersal distance. Even during the *Continuous* epoch, prior to patch isolation, rare alleles were lost due to drift. As anticipated, this process became more pronounced when dispersal was limited by impermeable barriers (the *Isolated Patches* epoch). The loss of allelic richness and evenness during patch

isolation was most pronounced in the small patches, and least severe in the large patches. Short-distance dispersal also influenced loss of allelic richness and evenness in both epochs, but only in medium and small patches. A heterozygosity deficit was observed when dispersal distances were short, but only in large and medium patches.

Evolutionary Ecology

Selection pressure was initiated at the beginning of the final landscape epoch, at time step 3000. Effective carrying capacity and population sizes emerged mechanistically from our model. Population size was most stochastic in the smaller patches, and most stable in the large patches (Fig 6). A nonzero probability of extinction was observed only for the small patches, and was greatest when the dispersal distances were short and movement barriers were present. The simulated adaptation to local conditions (time steps 3000-5000) effectively increased patch carrying capacity. These effects of adaptation were more pronounced when selection pressure was strong.

Fig 6. Observed trends in population size stratified by patch size. Results from all ten simulation replicates are shown. Data are displayed for an additional 1000 time steps during the final landscape epoch in order to better visualize the long-term population response to selection pressure. Carrying capacity was effectively increased by local adaptation, but the magnitude of this effect depended on the strength of selection. Extinction events can be inferred from occasional very low population sizes observed for the small patches (note the logarithmic vertical axis).

The frequency of the adaptive alleles at loci L7 and L10 increased globally across all subpopulations as selection acted locally and migration moved the adaptive alleles across the landscape. However, the effect of local selection at Locus L6 was counteracted by asymmetric migration (see Fig 2) between adjacent patches of different habitat types. In small patches, local selection was almost always swamped by migration from the neighboring large patches, except when selection was strong, dispersal distance was short, and gap permeability was low. Otherwise, for the small and medium patches, the extent to which selection was swamped by migration varied more continuously based on the strength of selection, barrier gap

permeability, and dispersal distance. In large patches, selection was often able to act effectively regardless of selection strength or migration rate, except when selection was weak and dispersal distance was long.

Discussion

We attempted to develop the simplest individual-based model capable of clearly illustrating how spatial structure drives eco-evo dynamics. By imposing landscape change on otherwise functionally-static eco-evolutionary models, we were able to alter key emergent forces such as gene-flow, genetic drift, and adaptive selection, as well as demo-genetic responses including population size, probability of extinction, and allele frequencies. Our model also demonstrated how critical demo-genetic traits, for example generation time and migration rate, can arise from a parsimonious mechanistic model, rather than being specified *a priori*.

Landscape Genetics

A common approach in landscape genetics is to assume that the current genetic pattern is a result of landscape pattern (with some lag-time) represented by resistance surfaces. Hypothetical "resistance distances" between individuals are compared to actual genetic distances between sampled individuals (or groups), and tests are performed to infer which elements in the landscape most strongly influence gene-flow. But complex movement behavior and dynamic landscape histories are not easily captured by resistance surfaces, and this in turn limits our ability to identify causal processes and detect past gene-flow [36, 37].

We demonstrated how the cumulative effects of gene-flow, genetic drift, and selection can be simulated over several hypothetical landscape histories. The resulting inter-individual genetic distance matrices simultaneously captured the influences of dynamic landscape structure, dispersal behavior, and demography. Long-distance dispersal behavior tended to swamp any effect of landscape history when landscape connectivity was high. This suggests it may be difficult to identify past landscape discontinuities when dispersal distances are long relative to the spatial extent of the study area.

Population Genetics

An ongoing challenge for empirical population geneticists and phylogeneticists has been determining the number of populations via analysis of genetic clustering patterns. Our model demonstrates that small amounts of IBD inflate such inferences, and that popular statistical solutions for choosing between possible numbers of genetic clusters are prone to overestimation [38].

Our approach offers population geneticists a methodology that allows migration rates to emerge from species-landscape interactions. Our model demonstrates how a simple alteration of barrier gap permeability can produce departures from the “one-migrant-per-generation” theoretically-derived guideline for re-establishing genetic panmixia [39]. We observed that, with short-distance dispersal behavior, the inference of population structure was similar when that structure resulted from either IBD or from gene-flow following a period of isolation, highlighting the difficulty in disentangling past genetic history from contemporary genetic structure.

Conservation Biology

Forecasting genetic degradation in small populations facing extinction is a challenging task that should ideally acknowledge the influences of both demographic and genetic processes. Our methodology addresses this challenge, as illustrated by two examples from our results. First, rare alleles were lost from the small patches, even when the landscape was fully connected; and the severity of this loss was more extreme when individuals dispersed short distances. Second, the loss of allelic evenness we observed exhibited different patterns depending on patch size. In the small patches, the allelic evenness eventually approached zero, due to single allele fixation, regardless of initial allele frequencies or dispersal distance. Conversely, in the large patches, allelic evenness varied depending upon initial conditions, but did not vary with dispersal distance. We observed a combined effect of dispersal distance and initial conditions in the medium patches, where limiting dispersal distance increased the loss of allelic evenness, but the steady-state depended on initial conditions. Traditional simulation approaches would associate a single trend with any given landscape.

These findings highlight how eco-evo simulators can add realism to the forecasts of genetic degradation used in conservation, and demonstrate how future studies might improve our understanding of inbreeding and outbreeding depression, better explore the relative importance of genetic versus demographic components of viability, and more.

Evolutionary Ecology

The HexSim modeling platform allows users to develop dynamic feedback loops linking evolutionary and ecological forces. Our model captured the counteracting forces of local selection and asymmetric migration from spatially proximal sub-populations. Adaptive alleles increased in frequency in spatially discontinuous landscape patches due to local selection, while migration rates between patches varied by patch size, dispersal behavior, and barrier gap permeability. The increase in adaptive alleles was most pronounced when selection was strong, yet this effect was not observed in the small and medium-sized patches when dispersal distance was short and barrier gap permeability was low, presumably because stochastic drift was then the driving force. When selection was weak, asymmetric migration between the large and proximate smaller patches produced highly variable changes in the frequency of the locally adaptive allele in the smaller patches. Even in the large patches, where in the absence of drift, selection might be expected to act efficiently, the anticipated changes in adaptive alleles were only observed when dispersal ability was limited and barrier gap permeability was low. The interplay between local selection and asymmetric migration that we found in our smaller patches demonstrates what we might expect to see as species' ranges shift due to climate change.

Conclusions

Contemporary ecological theory reflects a scientific world-view heavily influenced by mathematics and simulation models. But these useful abstractions only have practical value when equations can be formulated and solved, or source code can be designed and executed; and as is true with science in general, ecologists have made use of simplifying assumptions in order to keep model development tractable. The cost of these simplifications has been that our

theory can lack rigorous grounding in the very biological detail that we know governs species' interactions with their environments, and with each other. Simplifying assumptions are a practical necessity, but we should resist becoming too comfortable with them.

Our study used a two-pronged approach to examine these issues. First, we illustrated how advanced software tools (HexSim, in our case) allow researchers to explore complex biological mechanisms while retaining species-landscape and intraspecific interactions. Second, we examined specific simplifying assumptions that have shaped the development of theories in landscape genetics, population genetics, conservation biology, and evolutionary ecology. For each discipline, we used our hypothetical model system to illustrate how renewed attention to pattern and process can highlight the limits of existing theory. Our objective is not to find fault in past work, but rather to improve our models by challenging the assumptions upon which they have been constructed. To be sure, eco-evo theories of the future will extend existing models, not discard them. And the next generation of eco-evo theory will better explain patterns and processes when landscapes are spatially and temporally dynamic, species' life histories and genetics are complex and interacting, and when the details matter.

The simulations described here are, by design, simple and straightforward. But they illustrate fundamental biological forces, track familiar observable responses, and address pressing challenges in the fields of landscape genetics, population genetics, conservation biology, and evolutionary ecology. We also expect our methods to have applications in the fields of phylogenetics, phylogeography, medical science, and evolutionary theory. For example, phylogeneticists might simulate past species' range shifts, model incomplete lineage sorting, or ask how linkage affects coalescent metrics. Phylogeographers may explore the stability of admixture zones over time, or improve the demarcation of evolutionary significant units. More generally, future applications of our methods will benefit from the HexSim modeling platform's ability to incorporate dynamic geographically realistic landscapes, and to simulate real species, interactions, and disturbance regimes.

While our simulations included only simple microsatellite-like genotypes, we anticipate future projects will investigate how mode of inheritance, linkage between alleles, initial geographic

distributions of allele frequencies, and multiple mutation regimes (e.g., stepwise, infinite alleles, or two-phase models) interact with landscape spatial pattern and movement. Conservation geneticists may simulate mutation rates based on exposure to spatially-distributed chemical mutagens encountered during dispersal and mitigated by selection against deleterious alleles. Similarly, rates and patterns of gene-flow may be investigated within the context of nuanced, biologically-realistic movement behaviors, including rare long-distance dispersal events, site fidelity and memory, attraction and avoidance, sex-specific behaviors, to name a few. HexSim's design makes all of these simulation strategies possible, and more, while still being an ideal platform for simple models like the one explored in this study. Our methodology can also be extended to capture complex intermediate relationships between genotype, phenotype, and fitness, thus opening the door to evolutionary modeling at the level of genes, phenotypic heritable traits, populations, or even communities.

Acknowledgements

Allen Brookes developed the source code for HexSim and has been an invaluable team member. David Beck at the University of Washington eScience Institute aided in data management and processing. The information in this document has been subjected to review by the US EPA Center for Public Health and Environmental Assessment, and approved for publication.

References

1. Martens EA, Kostadinov R, Maley CC, Hallatschek O. Spatial structure increases the waiting time for cancer. New J Phys. 2011; 13 (11): 115014. https://doi.org/10.1088/1367-2630/13/11/115014

2. Walther GR, Post E, Convey P, Menzel A, Parmesan C, Beebee TCJ, Fromentin JM, Hoegh-Guldberg O, Bairlein F. Ecological responses to recent climate change. Nature. 2002; 416 (6879): 389–95. https://doi.org/10.1038/416389a

3. Waldvogel AM, Feldmeyer B, Rolshausen G, Exposito-Alonso M, Rellstab C, Kofler R, Mock T, Schmid K, Schmitt I, Bataillon T, Savolainen O, Bergland A, Flatt T, Guillaume F,

Pfenninger M. Evolutionary genomics can improve prediction of species' responses to climate change. Evol Lett. 2020; 4: 4-18. https://doi.org/10.1002/evl3.154

4. Kinnison MT, Hendry AP. The pace of modern life II: from rates of contemporary microevolution to pattern and process. In Microevolution rate, pattern, process, edited by A. P. Hendry and M. T. Kinnison. Contemporary Issues in Genetics and Evolution 8. Springer Netherlands. 2001; 145–64. https://doi.org/10.1007/978-94-010-0585-2_10

5. Thompson JN. Rapid Evolution as an ecological process. Trends Ecol Evol. 1998; 13 (8): 329–32. doi:10.1016/S0169-5347(98)01378-0. https://doi.org/10.1016/S0169-5347(98)01378-0

6. Sinervo B, Svensson E, Comendant T. Density cycles and an offspring quantity and quality game driven by natural selection. Nature. 2000; 406 (6799): 985–88. https://doi.org/10.1038/35023149

7. Yoshida T, Jones LE, Ellner SP, Fussmann GF, Hairston NG. Rapid evolution drives ecological dynamics in a predator–prey system. Nature. 2003; 424 (6946): 303–6. https://doi.org/10.1038/nature01767

8. Hairston NG, Ellner SP, Geber MA, Yoshida T, and Fox JA. Rapid evolution and the convergence of ecological and evolutionary time. Ecol Lett. 2005; 8 (10): 1114–27. https://doi.org/10.1111/j.1461-0248.2005.00812.x

9. Pelletier F, Clutton-Brock T, Pemberton J, Tuljapurkar S, Coulson T. The evolutionary demography of ecological change: linking trait variation and population growth. Science. 2007; 315 (5818): 1571–74. https://doi.org/10.1126/science.1139024

10. Reznick DN, Bassar RD, Handelsman CA, Ghalambor CK, Arendt J, Coulson T, Potter T, Ruell EW, Torres-Dowdall J, Bentzen P, Travis J. Eco-evolutionary feedbacks predict the time course of rapid life-history evolution. Am Nat. 2019; 194(5): 671-692. https://doi.org/10.1086/705380

11. Oke KB, Cunningham CJ, Westley PAH et al. Recent declines in salmon body size impact ecosystems and fisheries. Nat Commun. 2020; 11, 4155. https://doi.org/10.1038/s41467-020-17726-z

12. Bassar RD, Coulson T, Travis J, Reznick DN. Towards a more precise – and accurate – view of eco-evolution. Ecol Lett. 2021; 24(4): 623-625. https://doi.org/10.1111/ele.13712

13. Zeller KA, Lewsion R, Fletcher RJ, Tulbure MG, Jennings MK. Understanding the importance of dynamic landscape connectivity. Land. 2020; 9(9), 303. https://doi.org/10.3390/land9090303

14. Schumaker NH, Brookes A. HexSim: a modeling environment for ecology and conservation. Landsc Ecol. 2018; 33:197-211. https://doi.org/10.1007/s10980-017-0605-9

15. Balkenhol N, Waits LP, Dezzani RJ. Statistical approaches in landscape genetics: an evaluation of methods for linking landscape and genetic data. Ecography. 2009; 32 (5): 818–30. https://doi.org/10.1111/j.1600-0587.2009.05807.x

16. Landguth EL, Forester BR, Eckert AJ, Shirk AJ, Menon M, Whipple A, Day CC, Cushman SA. Modelling multilocus selection in an individual-based, spatially-explicit landscape genetics framework. Mol Ecol. Resour. 2020; 20(2), 605– 615. https://doi.org/10.1111/1755-0998.13121

17. Hoban S, Bertorelle G, Gaggiotti OE. Computer simulations: tools for population and evolutionary genetics. Nat Rev Genet. 2012; 13 (2): 110–22. https://doi.org/10.1038/nrg3130

18. Bradburd GS, Ralph PL. Spatial population genetics: it's about time. Annu Rev Ecol Evol Syst. 2019; 50: 427-449. https://doi.org/10.1146/annurev-ecolsys-110316-022659

19. Lande R. genetics and demography in biological conservation. Science. 1988; 241 (4872): 1455–60. https://doi.org/10.1126/science.3420403

20. Frankham R. Genetics and extinction. Biol Conserv. 2005; 126 (2): 131–40. https://doi.org/10.1016/j.biocon.2005.05.002

21. Akçakaya, HR, Root W. RAMAS GIS: linking spatial data with population viability analysis. Applied Biomathematics. 2005. Setauket, New York.

22. Lacy RC, Pollak JP. Vortex: A stochastic simulation of the extinction process. Version 10.5.5. 2021. Chicago Zoological Society, Brookfield, Illinois, USA.

23. Pelletier F, Garant D, Hendry AP. Eco-evolutionary dynamics. Philosophical Transactions of the Royal Society B: Biological Sciences. 2009; 364(1523), 1483-1489. https://doi.org/10.1098/rstb.2009.0027

24. Hendry AP. Eco-evolutionary dynamics. Princeton university press. 2016. 416 pages. ISBN: 9780691204178

25. Schoener TW. The newest synthesis: understanding the interplay of evolutionary and ecological dynamics. Science. 2011; 331(6016), 426-429. https://doi.org/10.1126/science.1193954

26. Legrand D, Cote J, Fronhofer EA, Holt RD, Ronce O, Schtickzelle N, Travis JM, Clobert J. Eco-evolutionary dynamics in fragmented landscapes. Ecography. 2017; 40(1), 9-25. https://doi.org/10.1111/ecog.02537

27. Pritchard JK, Stephens M, Donnelly P. Inference of population structure Using multilocus genotype data. Genetics. 2000; 155 (2): 945–59. https://doi.org/10.1093/genetics/155.2.945

28. Hamilton WD. The moulding of senescence by natural selection. J Theor. Biol. 1966; 12 (1): 12–45. https://doi.org/10.1016/0022-5193(66)90184-6

29. Wright S. Isolation by distance. Genetics. 1943; 28 (2): 114–38. https://doi.org/10.1093/genetics/28.2.114

30. Miller MP. Alleles In Space (AIS): Computer software for the joint analysis of interindividual spatial and genetic information. J Hered. 2005; 96 (6): 722–24. https://doi.org/10.1093/jhered/esi119

31. Paradis E, Claude J, Strimmer K. APE: Analyses of phylogenetics and evolution in R language. Bioinformatics. 2004; 20 (2): 289–90. https://doi.org/10.1093/bioinformatics/btg412

32. R Core Team. R: a language and environment for statistical computing. 2013. Vienna, Austria: R Foundation for Statistical Computing. https://www.R-project.org/

33. Pritchard JK, Wen X, Falush D. Documentation for structure software: Version 2.3. 2010. https://web.stanford.edu/group/pritchardlab/structure.html

34. Evanno G, Regnaut S, and Goudet J. Detecting the number of clusters of individuals using the software Structure: a simulation study. Mol Ecol. 2005; 14 (8): 2611–20. https://doi.org/10.1111/j.1365-294X.2005.02553.x

35. Keenan K, McGinnity P, Cross TF, Crozier WW, Prodöhl PA. diveRsity: An R package for the estimation and exploration of population genetics parameters and their associated errors. Methods Ecol Evol. 2013; 4 (8): 782–88. https://doi.org/10.1111/2041-210X.12067

36. Cushman SA & Landguth EL. Spurious correlations and inference in landscape genetics. Mol Ecol. 2010; 19(17), 3592-3602. https://doi.org/10.1111/j.1365-294X.2010.04656.x

37. Zeller KA, Creech TG, Millette KL, Crowhurst RS, Long RA, Wagner HH, Balkenhol N, Landguth EL. Using simulations to evaluate Mantel-based methods for assessing landscape resistance to gene flow. Ecol Evol. 2016; 6: 4115-4128. https://doi.org/10.1002/ece3.2154

38. Waples RS, Gaggiotti O. What is a population? An empirical evaluation of some genetic methods for identifying the number of gene pools and their degree of connectivity. Mol Ecol. 2006; 15 (6): 1419–39. https://doi.org/10.1111/j.1365-294X.2006.02890.x

39. Lande R, Barrowclough GF. Effective population size, genetic variation, and their use in population management. Pages 87-123 in M. E. Soule, editor. Viable populations for conservation. Cambridge University Press, Cambridge, England. 1987.

Response to Reviewers
PONE-D-22-20501_R1

Reviewer-1 developed a 9-page document as part of their assessment of our revised manuscript. This document contains 25 sections, which we refer to below as *statements*. Of the 25 statements, 4 concern our Introduction, 11 concern our Methods section, and 10 concern our Results section.

Every reviewer statement is accompanied by a range of line numbers, and begins with an overview of what we said in that portion of the manuscript. These passages are largely copied directly from our text, but the reviewer has sprinkled in some of their own words here and there.

No comments, questions, assertions, etc. have been added to the 14 statements that are matched to portions of our Introduction or Results sections. All of the reviewer's feedback is contained within the 11 statements that pertain to our Methods section. It appears the reviewer approached their task by (A) developing summaries, principally using our words, of most subsections within our Introduction, Methods, and Conclusions sections; and then (B) adding a comment to each summary that pertained to our Methods section. As a result, we have only provided nontrivial responses to the 11 reviewer statements that pertain to our Methods.

<u>All 11 of these statements are essentially the same</u>. They point out where our model lacks nuance that might be found in real landscapes and actual species.

The reviewer misses the point of our manuscript entirely. Yes, our model is simple by design, and by comparison to a complex natural system. We have intentionally designed it this way in order to maximize its generality. The simplicity and generality of our model are assets, and features that allow us to develop results and conclusions with relevance to all four focal disciplines discussed throughout our manuscript (landscape genetics, population genetics, conservation biology, evolutionary ecology). As is done in scientific research generally, we are beginning with simple theories and models; we will and add complexity in time, and then explore the veracity of those more nuanced models. That being said, our simple simulator already captures more demographic nuance, plus species-landscape and species-species interactions, than the majority of existing genetic models.

I was an associate editor Landscape Ecology for eight years, and have spent decades reviewing papers for journals. From that perspective, I found this reviewer's comments did not reflect an interest in understanding our improving our study; rather, they seemed purely intended to disparage our work. The reviewer's comments belie a lack of understanding and appreciation of even the most basic elements of our study. They appear disingenuous in that they don't attempt to critique our work, but instead read as a rushed and inexperienced attempt to discredit it. In fact, it seems the reviewer likely intended to pen 14 additional concerns (4 for the Introduction and 10 for the Results), but just couldn't think of anything to say.

Below, we address each of the reviewers 25 statement separately, in detail. We first copy the reviewer statements exactly as they were provided to us. We then add our response to the end of each statement. There is repetition in the reviewer statements, and thus also in our responses.

Reviewer Statement 1

Line 72-80

you provided that the field of landscape genetics is relatively new and still evolving, and as a result, the available landscape genetics modeling platforms may have limited spatial, demographic, or behavioral sophistication, or may not be able to incorporate mechanisms underlying species-landscape interactions or simultaneously simulate multiple interacting eco-evo drivers of gene-flow. This could potentially limit the ability of these models to accurately simulate complex movements and resulting gene-flow, and may restrict their ability to fully capture the influence of landscape pattern on gene-flow. Another is that the standard approach of comparing inter-individual genetic distance to metrics of landscape structure, such as cost-distance across a resistance surface, is it sufficient for fully understanding the influence of landscape pattern on gene-flow, and may not adequately account for other factors that may also be important in shaping gene-flow.

<u>Authors' Response</u>:

Here, the reviewer is restating what we wrote in this portion of the manuscript. There does not appear to be a question, comment, or actionable statement for us to respond to.

Reviewer Statement 2

Line 82-92

the classic population genetic models often assume that complex biological forces such as selection and mutation rates are spatially constant and may not adequately account for spatial heterogeneity and dynamics that may influence population size, density, dispersal flux, or local adaptation. Additionally, these models often reduce spatial complexity to discrete populations and characterize migration processes using a single univariate input parameter, which may not adequately capture the complexity of movement and mating behavior that may govern gene-flow. These simplifications may facilitate the generation of theory, but may also limit the ability of these models to accurately represent systems in which space is heterogeneous and dynamic or gene-flow is governed by complex movement and mating behavior. such systems may be limited and may not adequately account for the influence of landscape structure or non-random mate-choice on gene-flow.

<u>Authors' Response</u>:

Here, the reviewer is restating what we wrote in this portion of the manuscript. There does not appear to be a question, comment, or actionable statement for us to respond to.

Reviewer Statement 3

Line 94-103

you provided that existing tools and models for forecasting population viability, such as RAMAS GIS or Vortex, often simplify the linkages between ecological and evolutionary processes and is

it capable of simulating complex interacting demographic and genetic traits. This may limit their ability to accurately forecast how landscape changes will impact a population's size, distribution, mate-finding, or natural selection, and may reduce the utility of their forecasts for conservation purposes. Additionally, the relative importance of demography versus genetics in influencing population viability has been actively debated in the conservation literature, and the lack of models or tools that are capable of simultaneously accounting for both of these factors may further hinder the ability to accurately forecast population viability in the face of landscape change.

<u>Authors' Response</u>:

Here, the reviewer is restating what we wrote in this portion of the manuscript. There does not appear to be a question, comment, or actionable statement for us to respond to.

Reviewer Statement 4

Line 104-130

The inclusion of detailed spatial patterns and biologically realistic processes in eco-evo models may substantially enhance their realism and defensibility, but may also complicate the models, especially those that are already mechanistically-rich and integrate demography, movement, and gene flow. This complexity may make it more difficult for readers to evaluate the assertions made in these models and to understand the mechanics involved in incorporating spatial structure into genetic models. Additionally, the trade-off between realism, in the form of landscape structure and movement behavior, and mechanistic clarity in the pursuit of enhanced realism may limit the ability of these models to fully capture the complexity of eco-evo processes in natural systems.

<u>Authors' Response</u>:

Here, the reviewer is restating what we wrote in this portion of the manuscript. There does not appear to be a question, comment, or actionable statement for us to respond to.

Reviewer Statement 5

Line 134-146

The study is based on a simple heuristic simulation constructed within the HexSim modeling environment, which is a user-friendly, spatially-explicit, individual-based, demo-genetic modeling environment. This may limit the complexity and realism of the model and the range of processes it can capture, potentially leading to a lack of accuracy or generalizability in the results. Additionally, the use of a single species, minimally-complex landscape structure, and a limited number of treatment combinations may not adequately represent the complexity of natural systems and the many factors that can influence eco-evo dynamics. Is this study applicable to other systems ?

<u>Authors' Response</u>:

The first half of the reviewer's statement is a summary of content we provide in the manuscript. The second half contains a query regarding whether our use of a single species and a simple abstract landscape prevents our study from capturing the complexity found in natural systems.

Yes, our model is simple by design, and by comparison to a complex natural system. We have intentionally designed it this way in order to maximize its generality. The simplicity and generality of our model are assets, and the features that allows us to develop results and conclusions with relevance to all four focal disciplines discussed throughout our manuscript (landscape genetics, population genetics, conservation biology, evolutionary ecology). As is done in scientific research generally, we begin here with simple theories and models; we will and add complexity in time, and then explore the veracity of those more nuanced models.

As the reviewer correctly points out, we have clearly stated in our manuscript (in this and other locations) that our goal was to construct and explore a simple and heuristic demo-genetic simulation model. We designed our study to help readers fully grasp and adopt our novel methods, without becoming distracted by myriad landscape- or species-specific details. This is very standard and highly appropriate when developing methods papers such as ours. That being said, our simple simulator already captures more demographic nuance, plus species-landscape and species-species interactions, than the majority of existing genetic models.

We designed our manuscript to inspire readers from multiple disciplines, and encourage them to adopt and apply our methods. These future applications (including pending studies of our own) will employ actual landscapes and make use of empirically-derived demographic and genetic information. In the current study, we provide a roadmap for these future studies -- a roadmap that is easy to follow because we have limited the complexity of our heuristic model system. Readers who are interested in merging our methods with detailed species and landscape information need only turn to the HexSim website for inspiration and relevant examples. At that website, we provide a list of almost 70 peer-reviewed publications in which HexSim has already been applied in a wide range of settings and systems.

In summary, the reviewer's observation reflects a key design feature of our study – one that we feel is appropriate, and has been thoughtfully articulated throughout the manuscript. We do not to suggest that our simple heuristic system is realistic. In contrast, we designed and used it as a vehicle for exploring our novel methods, and illustrating how a better accounting for the role of space in the four focal disciplines might help generate future scientific advancements.

For these reasons, we have not changed the manuscript in response to this reviewer statement.

Reviewer Statement 6

Line 148-158

the landscape used in the study is artificial and simplified, consisting of only six habitat patches of different sizes that are isolated from a surrounding non-habitat matrix. Is it accurately represent real landscapes, which are typically more complex and diverse and is it include a variety of habitat types and structures ?. Additionally, the use of movement barriers to isolate the patches from each other at certain times and allow limited dispersal at other times

accurately represent or not the natural barriers and corridors that influence gene flow in real systems? . Additionally, the use of a uniform potential for crossing all patch interfaces accurately represent or not represent the heterogeneous nature of real landscapes and the different costs and benefits of movement between different patches.

Is the findings of this study applicable to other systems or fully capture the complexity of eco-evo processes in nature?

<u>Authors' Response</u>:

These reviewer questions are quite similar to those from *Reviewer Statement 5*, above. For background, please see our *Authors Response* to *Reviewer Statement 5*.

We designed our study so that the primary results could be anticipated, even as these outcomes change depending on the study *epoch* (see manuscript). Specifically, the use of six landscape patches arrayed as they are, including barriers we could carefully control, allowed us to search for cases where our spatial IBM's emergent properties departed from the expectations of existing theories and methods.

Of course, we are not suggesting that our landscape or barriers mimic those found in the real world. The reviewer here misses the point of our manuscript entirely. The purpose of our study is to compare new models that carefully account for space to old ones that use simplifying assumptions to avoid having to do so. Our study landscape is ideally suited to this purpose. In essence, the reviewer is suggesting we might have instead written a completely different manuscript. Such manuscripts will be written, but they will rely on this work as a foundation.

For these reasons, we have not changed the manuscript in response to this reviewer statement.

Reviewer Statement 7

Line 166-190

It is not clear from this description what the specific goals of the study or how the use of a heuristic simulation with a simple, artificial landscape and a limited set of life history events and species-landscape interactions is intended to contribute to the understanding of eco-evo dynamics. Additionally, the use of uniformly distributed reproductive rates and resource acquisition from a roughly circular neighborhood may accurately reflect or not reflect the complexities of natural systems. It would be helpful to have more information about the intended realism of the model and how the chosen design decisions support the goals of the study.

Overall, it would be useful to consider ways of improving the realism and complexity of this simulation, such as incorporating mechanisms for mutation and more nuanced models of adaptation.

<u>Authors' Response</u>:

We initially articulate the goals of the study in the last paragraph of the Introduction. The lines (166-190) cited by the reviewer are in fact part of our Methods section. In this last paragraph of the Introduction, we state:

> Below, we describe a relatively simple theoretical model designed to illustrate how the explicit inclusion of detailed spatial patterns and biologically realistic processes might facilitate the development of new eco-evo theory and applications. We demonstrate model relevance by highlighting results that (a) illustrate a priori expectations of a core eco-evo dynamic fundamental to a discipline, and (b) illustrate how additional biological and spatial realism might contribute to new theory and improve our confidence in modeling applications (Table 1). Our primary goals include both evaluating and articulating the benefits derived from adding spatial structure to genetic models, and illustrating the mechanics involved in doing this well. While the incorporation of space can substantially enhance realism and defensibility, it also complicates models, especially those which are already mechanistically-rich (e.g., that integrate demography, movement, and gene flow). In pursuit of the former goal, we trade away elements of realism, in the form of landscape structure and movement behavior, for mechanistic clarity. Readers evaluating our assertions will benefit from the simplicity of our simulations, as the number of drivers and responses they must track is limited. In regards to the latter goal, our work constitutes an important extension to the existing descriptions and applications of HexSim [14]. Readers interested in adopting this software will benefit from the case studies discussed here, as they provide detail on HexSim genetics that is unavailable in previous publications.

We suggest that this statement describing our study goals is exceedingly clear, and that we also have used this paragraph to articulate our landscape and species life history design criteria. As it is very traditional to place such a paragraph at the end of a paper's Introduction section, we're a bit perplexed regarding how the reviewer missed this content.

The reviewer goes on to suggest that:

- It would be helpful to have more information about the intended realism of the model and how the chosen design decisions support the goals of the study.
- Overall, it would be useful to consider ways of improving the realism and complexity of this simulation, such as incorporating mechanisms for mutation and more nuanced models of adaptation.

In addressing the first of these two points, we suggest that this information is in fact contained in the paragraph from the Introduction that we've copied above. Further, we revisit those issues in multiple places throughout the manuscript.

In addressing the second of these two points, we suggest that (again) the reviewer is missing the point of the manuscript entirely. Please see our responses (above) to *Reviewer Statement 5* and *Reviewer Statement 6* for background and further detail.

For these reasons, we have not changed the manuscript in response to this reviewer statement.

Reviewer Statement 8

Line 191-205

There are several issues with this method for simulating population genetics and evolution:

1. Using a fixed set of initial allele frequencies and not allowing for mutation means that the gene pool of the population is fixed and not subject to change over time. This means that the population will not be able to adapt to changing environments or selective pressures.
2. The use of pure neutrality for five of the loci may not be realistic, as many loci in real populations are subject to some degree of selective pressure, even if they do not directly impact survival or reproduction.
3. The use of a fixed set of locally adapted alleles may not accurately reflect the complex and multifaceted nature of adaptation in real populations, which can involve a wide range of genetic and environmental factors.
4. The decision to initiate selection at a specific time step (3000) may not be justified and could potentially lead to inflated estimates of the strength of selection.

***Authors' Response*:**

Regarding item 1. The HexSim model can simulate mutation events quite easily and effectively. However, simulating the influence of mutation lies entirely outside the scope of the current study. Our goal here is not to study the evolution of an actual species in a real landscape.

Regarding items 2 & 3. As we explain carefully throughout earlier portions of this *Response to Reviewers* document, the goal of this study was not to simulate a realistic landscape or species.

Regarding item 4. This is a bit of an odd comment, as the reviewer is descending quite far into minutiae and conjecture. We fear the reviewer has not clearly understood the study goals.

For these reasons, we have not changed the manuscript in response to this reviewer statement.

Reviewer Statement 9

Line 206-222

1. Using a binary landscape with habitat patches embedded within a non-habitat matrix accurately reflect or not reflect the complex and varied nature of real landscapes, which can have multiple habitat types and multiple scales of patchiness.
2. Is the use of a fixed set of movement barriers and gap permeabilities adequately capture the dynamic and variable nature of movement in real populations, which can be influenced by a wide range of factors such as individual behavior, habitat quality, and environmental conditions.
3. Is the decision to impose absolute movement barriers justified and could potentially lead to estimates of the strength of drift and isolation by distance.

4. Is the use of habitat types that confer increased fitness to specific genotypes accurately reflect the complex and multifaceted nature of adaptation in real populations, which can involve a wide range of genetic and environmental factors.

Authors' Response:

Regarding items 1 & 2. The reviewer previously articulated these concerns. Please see our responses (above) to *Reviewer Statement 5* and *Reviewer Statement 6*.

Regarding item 3. There are no barriers to movement in Epoch 1. We use absolute movement barriers in Epoch 2, so as to completely separate the six landscape patches. Epochs 3 & 4 use movement barriers that are permeable in places. Of course, changing the nature of the movement barriers can be expected to change the simulation results. Our experiments were designed to illustrate how a model that carefully incorporates spatial structure can produce results that differ from competing non-spatial or pseudo-spatial models. For this purpose, our study's movement barrier design works exceedingly well.

Regarding item 4. As we have discussed at length throughout this document (in response to similar questions), the goal of this study was not to simulate a realistic landscape or species.

For these reasons, we have not changed the manuscript in response to this reviewer statement.

Reviewer Statement 10

Line 223-237

1. The use of per-capita homozygosity as a measure of genetic diversity may not accurately reflect the complexity of real population genetics, as it does not consider factors such as the number and distribution of alleles or the relationships between individuals.
2. The decision to track only individual genotypes and not pedigree information may limit the types of analyses that can be performed, as pedigree information can provide important insights into the relationships between individuals and the patterns of inheritance in a population.
3. The use of generation time as the average age of reproducing females may not accurately reflect the complexity of real population demography, as it does not consider factors such as mortality rates or the age distribution of the population.
4. The definition of migration as the movement of individuals between patches that subsequently reproduce there may exclude important types of movement, such as exploratory or dispersal movements that do not result in reproduction.

Authors' Response:

As we have discussed throughout this document, the goal of this study was not to simulate a realistic landscape or species. These four items are all purely speculative, they focus on levels of biological detail much finer than those our study simulates, and they do not contain specific questions or actionable statements. These assertions do, however, strongly reinforce our

concern that the reviewer has failed to both grasp the goals of our study, or appreciate its results and conclusions.

For these reasons, we have not changed the manuscript in response to this reviewer statement.

Reviewer Statement 11

Line 239-256

1. The use of visual assessment of correlograms and dispersal kernels may not provide a reliable or objective measure of the degree of isolation by distance in the population.
2. The decision to randomly select a single replicate simulation and a subset of individuals from each patch location may not accurately reflect the overall patterns in the population and could potentially introduce bias.
3. The use of a simple metric of pairwise genetic distance based on the number of differing alleles may not accurately reflect the complexity of real population genetics and could potentially overlook important factors such as the frequencies and distributions of alleles in the population.
4. The use of triangular matrices to visualize pairwise genetic distances may not be the most effective way of representing this data, as it does not clearly show the relationships between all pairs of individuals.

<u>Authors' Response</u>:

The reviewer's four statements are all speculative, and none could possibly yield a simple definitive answer. These concerns would need to be uniquely reevaluated in every study.

In our simple heuristic study system, the visual assessment of correlograms and dispersal kernels is actually quite appropriate, and it produces accurate assessments of the system dynamics. In more complex systems, this would not necessarily be true.

Similarly, our decision to use results from a single replicate simulation to compute inter-individual genetic distances is reasonable give our study design. In a real-world system where the contemporary distribution of genotypes reflects a long history of landscape change and disturbance, other analytic methods are likely to be more appropriate.

The same is true for our computation of pairwise genetic distances. These points all underscore our previous assertion -- that the heuristic design of our landscape and species is an <u>asset</u>, not a liability as the reviewer seems to repeatedly imply.

Finally, we disagree completely with the reviewer's statement about our use of triangular matrices to visualize pairwise genetic distances. This method, applied in the construction of Figure 4, captures an enormous amount of information and displays it in format that allows readers to quickly and easily grasp the take-home messages and insights. It is an elegant design.

For these reasons, we have not changed the manuscript in response to this reviewer statement.

Reviewer Statement 12

Line 257-276

It appears that the authors used a Bayesian clustering method called STRUCTURE to analyze population genetic structure in this study. They collected genotype data stratified by putative populations (patches), and imported this data into STRUCTURE for analysis. The authors encountered the issue of unequal sample sizes in their patches, which can interfere with the assessment of the number of subpopulations and assignment probabilities in STRUCTURE. To address this issue, they randomly selected a fixed number of individuals (n=25) from each patch for analysis. In cases where there were fewer than 25 individuals in a patch, they included some genotypes multiple times until each patch had a sample of 25, for a total of 150 individuals. The authors tested a range of possible values for the number of unique genetic clusters (K) from 1 to 20, and used two methods to identify the best-supported K values: plotting the replicate average Ln P(D|K) and visually determining the minimum K of the curve's asymptote, and using Evanno's ΔK method. If there was a close tie between the supported K values from these two methods, the authors considered them all for individual assignment analyses.

It is worth noting that using a fixed number of individuals (n=25) from each patch for analysis, regardless of the actual number of individuals present in each patch, may introduce biases into the analysis. Additionally, while the authors mention that they used the default settings of the admixture model and correlated allele frequencies in their STRUCTURE analyses, it would be helpful to provide more detail about these settings and how they were chosen. It would also be useful to explain why the authors expected the number of unique genetic clusters (K) to vary from 1 to 6 based on landscape structure

***Authors' Response*:**

The reviewer uses the first paragraph to restate what we wrote in this section of the manuscript. The reviewer's comments about program STRUCTURE, in the second paragraph, make no sense to us. Our use of STRUCTURE is very standard, and the results we derived from our STRUCTURE assessments make sense in light of the study landscape and species. STRUCTURE-based analyses account for a very small part of our methods and results, and so we feel the current level of detail we provide regarding these methods is exactly right. All told, the reviewer appears to be simply going through every section of our Methods section, and pointing out every place where any of our techniques might conceivable prove inadequate in a more complex system. We don't believe this constitutes a meaningful critique of our work.

For these reasons, we have not changed the manuscript in response to this reviewer statement.

Reviewer Statement 13

Line 277-299

It appears that the authors calculated Per-capita Homozygosity as the percent of homozygous genotypes across all alleles and individuals within a given landscape patch. They also examined the effect of patch isolation on allele frequencies at the end of the Continuous and Isolated Patches epochs. To do this, they calculated three metrics of genetic degradation for each locus

in each subpopulation using the "divBasic" function from the "diveRsity" R package. These metrics were:

1. Allelic richness: the number of unique alleles per locus. All ten loci began with 5 alleles each.
2. Allelic evenness: a measure of the relative frequencies of different alleles at a locus. It is calculated as $\sum pi$ln(pi)/ ln(5), where pi is the frequency of the ith allele within each locus, and ln(5) is the maximum evenness in each locus, given that all were initiated with 5 alleles. The starting population had different initial allele frequencies corresponding to allelic evenness values of 1.0 = "Equal", 0.96 = "Unequal", and 0.72 = "Rare" (Table 2).
3. Heterozygosity deficit: a measure of the deviation of the observed number of heterozygous genotypes from the expected number. It is calculated as $Hobs - Hexp$, where $Hobs$ is the number of observed heterozygous genotypes and expected heterozygosity is calculated as: $Hexp$ = 1−∑ (qi)2 n 1 , where n represents the number of alleles, and qi is the frequency of the ith allele at a locus. Note that $Hexp$ is calculated based on the number of extant alleles at a locus, and will fluctuate dramatically as alleles are lost from a population. Therefore, unlike Allelic richness or Allelic evenness, this metric has a shifting baseline.

For each of these metrics, the authors calculated the mean and standard deviation across all replicates within the same patch size (small, medium, or large) and of the same initial allele frequency (equal, unequal, or rare) for each of the two time points of interest (at the end of the Continuous and Isolated Patches epochs).

It would be helpful to provide more information about the specific populations and the number of replicates used in this analysis, as well as a clear explanation of the reasoning behind the choice of patch sizes and initial allele frequencies.

<u>Authors' Response</u>:

As usual, these reviewer comments, except for the last short paragraph above, are mostly copied directly from our manuscript. They don't contain any questions or new observations. Regarding the final paragraph: the term "population", in the sense the reviewer uses the term, is an emergent property of our simulation model. We describe these populations extensively throughout the Results section of our manuscript. Is the reviewer suggesting that we should have placed our results within our Methods section? The manuscript clearly states that we performed 10 replicates per treatment for each HexSim simulation (lines 225-226), and that we performed 20 replicates of each STRUCTURE analysis (lines 270-272). Regarding the reviewer's comment asking for a clear explanation of the reasoning behind the choice of patch sizes and initial allele frequencies: we have already explained our reasoning in detail. The exact numbers of hexagons per patch, or alleles per locus are arbitrary and irrelevant. What is important is that the reader understands our model system, and has a sense for why it might behave in one way or another. This closing comment strikes us as remarkably disingenuous.

For these reasons, we have not changed the manuscript in response to this reviewer statement.

Reviewer Statement 14

Line no 300-308

Authors tracked population size for each patch across all time steps to measure the response to adaptation. They also calculated the change in frequency of adaptive alleles within each patch during the Semi-Connected with Local Selection epoch. This analysis focused on specific loci (L6-L10) that contained a mix of adaptive and neutral alleles. L6 contained two adaptive alleles and 3 neutral, while L7-L10 each contained one adaptive allele and 4 neutral. Some of the adaptive alleles were advantageous in half of the patches, while others were advantageous in the other half. The authors tracked the frequency of these alleles in order to examine the effect of local adaptation on allele frequency in combination with asymmetric migration from adjacent patches, where the opposite allele was advantageous.

***Authors' Response*:**

Here, the reviewer is restating what we wrote in this portion of the manuscript. There does not appear to be a question, comment, or actionable statement for us to respond to.

Reviewer Statement 15

Line no. 309-314

Authors ran 80 simulations on a Dell PowerEdge R820 server, and each individual model run took approximately four hours of processing time to complete. The authors suggest that the simulations could likely be replicated more quickly on a modern desktop or laptop computer, and that the use of the server simply made it possible to run all simulations simultaneously. The HexSim application and the simulation models described in the study are available at www.hexsim.net.

It is worth noting that the computational effort required for a study can vary significantly depending on the complexity of the models and the number of simulations run. Providing more information about the specific models and parameters used in the simulations, as well as software environment in which they were run, would be helpful for readers to better understand the computational demands of the study.

***Authors' Response*:**

The first paragraph above is copied directly from our manuscript. We don't understand the point of reviewer's second paragraph, except to note that it is consistent with the overall tone and rigor of the review. Of course, computational effort will vary based on model complexity and the number of replicate simulations. We have already provided extensive detail on our model design and parameters. Does the reviewer want to know if our model runs on Apple, Windows, or Linux? If there was any remaining doubt regarding the intent and veracity of this review, this second paragraph should remove that doubt.

As a result of this comment, we have decided to remove the entire subsection of the manuscript titled "Computational Effort and Model Availability" (formerly lines 309-314). We felt this section didn't add any substantial value to the manuscript. However, we did copy the following

text from that section before deleting it, and placed it to the end of the second paragraph of the Introduction: "Both the HexSim application and the simulation models described here are available at www.hexsim.net".

Reviewer Statement 16

Line 315-329

It appears that the age-class structure in the study resulted in an average age of reproducing females of 8.7 years, and each landscape epoch of 1000 simulation time steps (Fig 1) spanned approximately 115 generations. During the Semi-Connected epoch, the authors observed that migration rate between adjacent patches varied with dispersal distance, barrier gap permeability, and patch size (Fig 2). They found that the majority of individuals remained in their natal patch, and that migration rates were higher when individuals were assigned the long-distance dispersal behavior and the barrier gaps were highly permeable. The authors also observed that unequal perimeter-area ratios resulted in asymmetric migration, with smaller patches generating proportionately greater numbers of emigrants and larger patches receiving proportionately higher numbers of immigrants.

Authors' Response:

Here, the reviewer is restating what we wrote in this portion of the manuscript. There does not appear to be a question, comment, or actionable statement for us to respond to.

Reviewer Statement 17

Line 330-345

the results of a study that analyzed the effect of landscape and dispersal behaviors on gene flow in a population. The results indicate that short dispersal distances were associated with a higher degree of genetic relatedness (i.e., inbreeding) compared to long dispersal distances, as shown by the observed dispersal kernels. Additionally, the effectiveness of gene flow at mixing subpopulations was found to be influenced by landscape history and dispersal behavior, as well as by the permeability of barrier gaps and the strength of selection in certain landscape epochs.

Authors' Response:

Here, the reviewer is restating what we wrote in this portion of the manuscript. There does not appear to be a question, comment, or actionable statement for us to respond to.

Reviewer Statement 18

Line 353-390

Authors discussing the results of some simulations or experiments involving population genetics. The purpose of the simulations or experiments seems to be to examine how different factors, such as dispersal distance and barrier gap permeability, influence the genetic structure of a population. The text mentions three different epochs: Continuous, Isolated Patches, and Semi-

Connected. It appears that in the Continuous epoch, the population is treated as a single entity, while in the Isolated Patches epoch, the population is divided into distinct patches that are isolated from each other by absolute barriers. In the Semi-Connected epoch, the barriers between the patches are partially permeable, allowing for some level of gene flow between the patches. This mentions that the genetic structure of the population changes depending on the level of gene flow between the patches and the distance that individuals are able to disperse. It also mentions that two different methods, Evanno's method (E) and the visual interpretation of likelihood probabilities (L(K)), were used to infer the number of genetic subpopulations present in the population. It appears that the results of these methods varied depending on the dispersal distance and epoch, with Evanno's method tending to produce inflated estimates of the number of genetic subpopulations in the Isolated Patches epoch.

***Authors' Response*:**

Here, the reviewer is restating what we wrote in this portion of the manuscript. There does not appear to be a question, comment, or actionable statement for us to respond to.

Reviewer Statement 19

Line 391-412

some simulations or experiments that were conducted to examine the effect of different factors on the genetic structure of a population. The factors being studied include patch size, barrier gap permeability, and dispersal distance. The text mentions three different epochs: Continuous, Isolated Patches, and Semi-Connected. It appears that in the Continuous epoch, the population is treated as a single entity, while in the Isolated Patches epoch, the population is divided into distinct patches that are isolated from each other by barriers. In the Semi-Connected epoch, the barriers between the patches are partially permeable, allowing for some level of gene flow between the patches.

This mentions that per-capita homozygosity, which is a measure of the proportion of an individual's genome that is made up of identical alleles, varies depending on the epoch and patch size. It appears that homozygosity increases during the Isolated Patches epoch, particularly in small patches, but decreases during the Semi-Connected epoch. the loss of allelic richness and evenness, which are measures of the diversity of alleles within a population, is more pronounced in small patches and during short-distance dispersal.

***Authors' Response*:**

Here, the reviewer is restating what we wrote in this portion of the manuscript. There does not appear to be a question, comment, or actionable statement for us to respond to.

Reviewer Statement 20

Line 413-439

simulations or experiments that were conducted to examine the effect of different factors on the evolution of a population. The factors being studied include patch size, barrier gap

permeability, dispersal distance, and selection pressure. The text mentions that selection pressure was initiated at a certain time step, and that the population size and carrying capacity emerge mechanistically from the model. It appears that the population size is more stable in large patches and more prone to extinction in small patches, particularly when dispersal distances are short and movement barriers are present.

The frequency of adaptive alleles at certain loci increases globally across all subpopulations as selection acts locally and migration moves the adaptive alleles across the landscape. However, the effect of local selection at one of the loci is counteracted by asymmetric migration between patches of different habitat types. It appears that the extent to which selection is swamped by migration varies depending on patch size, strength of selection, barrier gap permeability, and dispersal distance.

***Authors' Response*:**

Here, the reviewer is restating what we wrote in this portion of the manuscript. There does not appear to be a question, comment, or actionable statement for us to respond to.

Reviewer Statement 21

Line 448-462

the challenges of using resistance surfaces, which are used to model gene flow in landscape genetics, to infer the influence of landscape elements on gene flow. The text mentions that resistance surfaces are limited in their ability to capture complex movement behavior and dynamic landscape histories, which can make it difficult to identify causal processes and detect past gene flow.

The simulations can be used to examine the cumulative effects of gene flow, genetic drift, and selection on genetic distance between individuals over several hypothetical landscape histories. It appears that long-distance dispersal behavior can swamp the influence of landscape history on genetic distance when landscape connectivity is high, making it difficult to identify past landscape discontinuities when dispersal distances are long relative to the spatial extent of the study area.

Authors' Response:

Here, the reviewer is restating what we wrote in this portion of the manuscript. There does not appear to be a question, comment, or actionable statement for us to respond to.

Reviewer Statement 22

Line 463-476

the challenges of inferring the number of populations from analysis of genetic clustering patterns, and the limitations of popular statistical methods for choosing between possible numbers of genetic clusters. The text mentions that small amounts of interbreeding between

close relatives, known as inbreeding depression (IBD), can inflate such inferences and lead to overestimation.

the authors have developed a methodology that allows migration rates to emerge from species-landscape interactions, and that the model demonstrates how alteration of barrier gap permeability can produce departures from the theoretical guideline of "one migrant per generation" for re-establishing genetic panmixia. authors mentions that with short-distance dispersal behavior, it can be difficult to disentangle past genetic history from contemporary genetic structure.

Authors' Response:

Here, the reviewer is restating what we wrote in this portion of the manuscript. There does not appear to be a question, comment, or actionable statement for us to respond to.

Reviewer Statement 23

Line 477-497

the challenges of forecasting genetic degradation in small populations facing extinction, and the usefulness of eco-evo simulators in addressing these challenges. The rare alleles are lost from small patches, even when the landscape is fully connected, and that the severity of this loss is more extreme when individuals disperse short distances. the loss of allelic evenness exhibits different patterns depending on patch size, with single allele fixation eventually approaching zero in small patches, but varying depending on initial conditions and not dispersal distance in large patches. In medium patches, the loss of allelic evenness increases with limiting dispersal distance, but the steady-state depends on initial conditions.

This suggests that these findings demonstrate how eco-evo simulators can add realism to the forecasts of genetic degradation used in conservation, and how future studies could improve our understanding of inbreeding and outbreeding depression, explore the relative importance of genetic versus demographic components of viability, and more.

Authors' Response:

Here, the reviewer is restating what we wrote in this portion of the manuscript. There does not appear to be a question, comment, or actionable statement for us to respond to.

Reviewer Statement 24

Line 495-511

discussing the capabilities of the HexSim modeling platform to capture the interplay between local selection and asymmetric migration in a spatially discontinuous landscape. The adaptive alleles increase in frequency due to local selection, but that this effect is not observed in small and medium-sized patches when dispersal distance is short and barrier gap permeability is low, likely due to the influence of stochastic drift. when selection is weak, asymmetric migration between large and proximate smaller patches produces highly variable changes in the frequency

of the locally adaptive allele in the smaller patches. Even in large patches, where selection might be expected to act efficiently, the anticipated changes in adaptive alleles were only observed when dispersal ability was limited and barrier gap permeability was low. authors suggests that this interplay between local selection and asymmetric migration demonstrates what might be expected as species' ranges shift due to climate change

***Authors' Response*:**

Here, the reviewer is restating what we wrote in this portion of the manuscript. There does not appear to be a question, comment, or actionable statement for us to respond to.

Reviewer Statement 25

Line 512-557

The results of the study suggest that advanced software tools like HexSim can be used to explore complex biological mechanisms while retaining species-landscape and intraspecific interactions. The study also highlights the limitations of existing theories in the fields of landscape genetics, population genetics, conservation biology, and evolutionary ecology due to simplifying assumptions. The authors suggest that future studies could improve models by challenging these assumptions and that the HexSim modeling platform could be used to investigate mode of inheritance, linkage between alleles, initial geographic distributions of allele frequencies, and multiple mutation regimes in relation to landscape spatial patterns and movement. The study also suggests that the methodology could be extended to capture intermediate relationships between species and their environments, and to simulate complex mechanisms of intraspecific interactions. Overall, the study suggests that eco-evo theories of the future will better explain patterns and processes when landscapes are spatially and temporally dynamic, species' life histories and genetics are complex and interacting, and when the details matter.

***Authors' Response*:**

Here, the reviewer is restating what we wrote in this portion of the manuscript. There does not appear to be a question, comment, or actionable statement for us to respond to.
