# Supplementary material for: Adding pattern and process to eco-evo theory and applications
Source: PLoS One. 2023 Mar 9;18(3):e0282535. doi: 10.1371/journal.pone.0282535 (PMC9997922; doi:10.1371/journal.pone.0282535)
Supplement: Attachment — Submitted filename: Response to Reviewers.pdf [file pone.0282535.s006.pdf]

Response to Reviewers
PONE-D-22-20501_R3

We developed this revision (#3) of our manuscript in response to a single new review by "Reviewer-3". We greatly appreciate Reviewer-3's time and insights, and have made changes to the manuscript that both embrace and expand upon these comments. We feel that this contribution made by Reviewer-3 has helped us improve the manuscript, and that these updates will be appreciated by readers.

Specifically:

- Reviewer-3 suggested that we add two additional citations to the subsection of the *Introduction* titled *Conservation Biology*. We have added those citations to this section, along with some new text to accompany them. See R3 citations 26 and 27.

- Reviewer-3 suggested that we add four additional citations to the subsection of the *Introduction* titled *Evolutionary Ecology*. We have added those citations to this section, along with some new text to accompany them. See R3 citations 32, 33, 34, and 35.

- To more thoroughly capture the spirit of Reviewer-3's comments and insights, we have also added some additional text and one citation (see R3 citation 21) to the end of the subsection of the *Introduction* titled *Population Genetics*.

- To further build upon the theme of Reviewer-3's comments, we also rewrote a small portion of the first paragraph of the *Introduction*. And here, we included two additional new citations (see R3 citations 12 and 13).

Thank you again for reading and commenting on our manuscript. We appreciate it very much.

Sincerely,

NH Schumaker and co-authors.
