# Supplementary material for: Adding pattern and process to eco-evo theory and applications
Source: PLoS One. 2023 Mar 9;18(3):e0282535. doi: 10.1371/journal.pone.0282535 (PMC9997922; doi:10.1371/journal.pone.0282535)
Supplement: Attachment — Submitted filename: comments.docx [file pone.0282535.s001.docx]

1. The research paper entitled “Adding Pattern and Process to Eco-Evo Theory and Applications. Short Title: Eco-Evo Theory and Applications” by Schumaker et al. presented a novel simulation  modeling approach for investigating eco-evolutionary dynamics, centered on the driving role of  landscape pattern. The manuscript is hard to catch what is the exact subject of the study described.
2. The introduction is also vague and somewhat confusing, and is not organized in a scientific fashion. What is the background of the research, why does it need to be investigated?
3. Introduction and conclusion are not at par with the hypothesis.
4. The methods are not clear and are very difficult to understand. It could be a little simple and illustrative.
5. Discussion can be improved.There is no crosstalk in the discussion. You can discuss with some of the cross-references then it will make more sense of the study.
6. The overall text is not written clearly. There is too much of wordiness in the lines. It should be removed to make it simple and crisp.
7. The HexSim approach that you used, has a global significance in conservation management for in particular or for any ecosystem in general?
