# Supplementary material for: Adding pattern and process to eco-evo theory and applications
Source: PLoS One. 2023 Mar 9;18(3):e0282535. doi: 10.1371/journal.pone.0282535 (PMC9997922; doi:10.1371/journal.pone.0282535)
Supplement: Attachment — Submitted filename: C PONE-D-22-20501_R1.docx [file pone.0282535.s003.docx]

Line 72-80

you provided that the field of landscape genetics is relatively new and still evolving, and as a result, the available landscape genetics modeling platforms may have limited spatial, demographic, or behavioral sophistication, or may not be able to incorporate mechanisms underlying species-landscape interactions or simultaneously simulate multiple interacting eco-evo drivers of gene-flow. This could potentially limit the ability of these models to accurately simulate complex movements and resulting gene-flow, and may restrict their ability to fully capture the influence of landscape pattern on gene-flow. Another is that the standard approach of comparing inter-individual genetic distance to metrics of landscape structure, such as cost-distance across a resistance surface, is it sufficient for fully understanding the influence of landscape pattern on gene-flow, and may not adequately account for other factors that may also be important in shaping gene-flow.

Line 82-92

the classic population genetic models often assume that complex biological forces such as selection and mutation rates are spatially constant and may not adequately account for spatial heterogeneity and dynamics that may influence population size, density, dispersal flux, or local adaptation. Additionally, these models often reduce spatial complexity to discrete populations and characterize migration processes using a single univariate input parameter, which may not adequately capture the complexity of movement and mating behavior that may govern gene-flow. These simplifications may facilitate the generation of theory, but may also limit the ability of these models to accurately represent systems in which space is heterogeneous and dynamic or gene-flow is governed by complex movement and mating behavior. such systems may be limited and may not adequately account for the influence of landscape structure or non-random mate-choice on gene-flow.

Line 94-103

you provided that existing tools and models for forecasting population viability, such as RAMAS GIS or Vortex, often simplify the linkages between ecological and evolutionary processes and is it capable of simulating complex interacting demographic and genetic traits. This may limit their ability to accurately forecast how landscape changes will impact a population's size, distribution, mate-finding, or natural selection, and may reduce the utility of their forecasts for conservation purposes. Additionally, the relative importance of demography versus genetics in influencing population viability has been actively debated in the conservation literature, and the lack of models or tools that are capable of simultaneously accounting for both of these factors may further hinder the ability to accurately forecast population viability in the face of landscape change.

Line 104-130

The inclusion of detailed spatial patterns and biologically realistic processes in eco-evo models may substantially enhance their realism and defensibility, but may also complicate the models, especially those that are already mechanistically-rich and integrate demography, movement, and gene flow. This complexity may make it more difficult for readers to evaluate the assertions made in these models and to understand the mechanics involved in incorporating spatial structure into genetic models. Additionally, the trade-off between realism, in the form of landscape structure and movement behavior, and mechanistic clarity in the pursuit of enhanced realism may limit the ability of these models to fully capture the complexity of eco-evo processes in natural systems.

Line 134-146

The study is based on a simple heuristic simulation constructed within the HexSim modeling environment, which is a user-friendly, spatially-explicit, individual-based, demo-genetic modeling environment. This may limit the complexity and realism of the model and the range of processes it can capture, potentially leading to a lack of accuracy or generalizability in the results. Additionally, the use of a single species, minimally-complex landscape structure, and a limited number of treatment combinations may not adequately represent the complexity of natural systems and the many factors that can influence eco-evo dynamics. Is this study applicable to other systems ?

Line 148-158

the landscape used in the study is artificial and simplified, consisting of only six habitat patches of different sizes that are isolated from a surrounding non-habitat matrix. Is it accurately represent real landscapes, which are typically more complex and diverse and is it include a variety of habitat types and structures ?. Additionally, the use of movement barriers to isolate the patches from each other at certain times and allow limited dispersal at other times accurately represent or not the natural barriers and corridors that influence gene flow in real systems? . Additionally, the use of a uniform potential for crossing all patch interfaces accurately represent or not represent the heterogeneous nature of real landscapes and the different costs and benefits of movement between different patches.

Is the findings of this study applicable to other systems or fully capture the complexity of eco-evo processes in nature?

Line 166-190

It is not clear from this description what the specific goals of the study or how the use of a heuristic simulation with a simple, artificial landscape and a limited set of life history events and species-landscape interactions is intended to contribute to the understanding of eco-evo dynamics. Additionally, the use of uniformly distributed reproductive rates and resource acquisition from a roughly circular neighborhood may accurately reflect or not reflect the complexities of natural systems. It would be helpful to have more information about the intended realism of the model and how the chosen design decisions support the goals of the study.

Overall, it would be useful to consider ways of improving the realism and complexity of this simulation, such as incorporating mechanisms for mutation and more nuanced models of adaptation.

Line 191-205

There are several issues with this method for simulating population genetics and evolution:

1. Using a fixed set of initial allele frequencies and not allowing for mutation means that the gene pool of the population is fixed and not subject to change over time. This means that the population will not be able to adapt to changing environments or selective pressures.
2. The use of pure neutrality for five of the loci may not be realistic, as many loci in real populations are subject to some degree of selective pressure, even if they do not directly impact survival or reproduction.
3. The use of a fixed set of locally adapted alleles may not accurately reflect the complex and multifaceted nature of adaptation in real populations, which can involve a wide range of genetic and environmental factors.
4. The decision to initiate selection at a specific time step (3000) may not be justified and could potentially lead to inflated estimates of the strength of selection.

Line 206-222Top of Form

Top of Form

1. Using a binary landscape with habitat patches embedded within a non-habitat matrix accurately reflect or not reflect the complex and varied nature of real landscapes, which can have multiple habitat types and multiple scales of patchiness.
2. Is the use of a fixed set of movement barriers and gap permeabilities adequately capture the dynamic and variable nature of movement in real populations, which can be influenced by a wide range of factors such as individual behavior, habitat quality, and environmental conditions.
3. Is the decision to impose absolute movement barriers justified and could potentially lead to estimates of the strength of drift and isolation by distance.
4. Is the use of habitat types that confer increased fitness to specific genotypes accurately reflect the complex and multifaceted nature of adaptation in real populations, which can involve a wide range of genetic and environmental factors.

Line 223-237

1. The use of per-capita homozygosity as a measure of genetic diversity may not accurately reflect the complexity of real population genetics, as it does not consider factors such as the number and distribution of alleles or the relationships between individuals.
2. The decision to track only individual genotypes and not pedigree information may limit the types of analyses that can be performed, as pedigree information can provide important insights into the relationships between individuals and the patterns of inheritance in a population.
3. The use of generation time as the average age of reproducing females may not accurately reflect the complexity of real population demography, as it does not consider factors such as mortality rates or the age distribution of the population.
4. The definition of migration as the movement of individuals between patches that subsequently reproduce there may exclude important types of movement, such as exploratory or dispersal movements that do not result in reproduction.

Line 239-256

1. The use of visual assessment of correlograms and dispersal kernels may not provide a reliable or objective measure of the degree of isolation by distance in the population.
2. The decision to randomly select a single replicate simulation and a subset of individuals from each patch location may not accurately reflect the overall patterns in the population and could potentially introduce bias.
3. The use of a simple metric of pairwise genetic distance based on the number of differing alleles may not accurately reflect the complexity of real population genetics and could potentially overlook important factors such as the frequencies and distributions of alleles in the population.
4. The use of triangular matrices to visualize pairwise genetic distances may not be the most effective way of representing this data, as it does not clearly show the relationships between all pairs of individuals.

Line 257-276

It appears that the authors used a Bayesian clustering method called STRUCTURE to analyze population genetic structure in this study. They collected genotype data stratified by putative populations (patches), and imported this data into STRUCTURE for analysis. The authors encountered the issue of unequal sample sizes in their patches, which can interfere with the assessment of the number of subpopulations and assignment probabilities in STRUCTURE. To address this issue, they randomly selected a fixed number of individuals (n=25) from each patch for analysis. In cases where there were fewer than 25 individuals in a patch, they included some genotypes multiple times until each patch had a sample of 25, for a total of 150 individuals. The authors tested a range of possible values for the number of unique genetic clusters (K) from 1 to 20, and used two methods to identify the best-supported K values: plotting the replicate average Ln P(D|K) and visually determining the minimum K of the curve's asymptote, and using Evanno's ∆K method. If there was a close tie between the supported K values from these two methods, the authors considered them all for individual assignment analyses.

It is worth noting that using a fixed number of individuals (n=25) from each patch for analysis, regardless of the actual number of individuals present in each patch, may introduce biases into the analysis. Additionally, while the authors mention that they used the default settings of the admixture model and correlated allele frequencies in their STRUCTURE analyses, it would be helpful to provide more detail about these settings and how they were chosen. It would also be useful to explain why the authors expected the number of unique genetic clusters (K) to vary from 1 to 6 based on landscape structure

Line 277-299

It appears that the authors calculated Per-capita Homozygosity as the percent of homozygous genotypes across all alleles and individuals within a given landscape patch. They also examined the effect of patch isolation on allele frequencies at the end of the Continuous and Isolated Patches epochs. To do this, they calculated three metrics of genetic degradation for each locus in each subpopulation using the "divBasic" function from the "diveRsity" R package. These metrics were:

1. Allelic richness: the number of unique alleles per locus. All ten loci began with 5 alleles each.
2. Allelic evenness: a measure of the relative frequencies of different alleles at a locus. It is calculated as ∑𝑝𝑖ln(𝑝𝑖)/ ln(5), where 𝑝𝑖 is the frequency of the 𝑖𝑡ℎ allele within each locus, and 𝑙𝑛(5) is the maximum evenness in each locus, given that all were initiated with 5 alleles. The starting population had different initial allele frequencies corresponding to allelic evenness values of 1.0 = "Equal", 0.96 = "Unequal", and 0.72 = "Rare" (Table 2).
3. Heterozygosity deficit: a measure of the deviation of the observed number of heterozygous genotypes from the expected number. It is calculated as 𝐻𝑜𝑏𝑠 −𝐻𝑒𝑥𝑝, where 𝐻𝑜𝑏𝑠 is the number of observed heterozygous genotypes and expected heterozygosity is calculated as: 𝐻𝑒𝑥𝑝 = 1−∑ (𝑞𝑖)2 𝑛 1 , where 𝑛 represents the number of alleles, and 𝑞𝑖 is the frequency of the 𝑖𝑡ℎ allele at a locus. Note that 𝐻𝑒𝑥𝑝 is calculated based on the number of extant alleles at a locus, and will fluctuate dramatically as alleles are lost from a population. Therefore, unlike Allelic richness or Allelic evenness, this metric has a shifting baseline.

For each of these metrics, the authors calculated the mean and standard deviation across all replicates within the same patch size (small, medium, or large) and of the same initial allele frequency (equal, unequal, or rare) for each of the two time points of interest (at the end of the Continuous and Isolated Patches epochs).

It would be helpful to provide more information about the specific populations and the number of replicates used in this analysis, as well as a clear explanation of the reasoning behind the choice of patch sizes and initial allele frequencies.

Line no 300-308

Authors tracked population size for each patch across all time steps to measure the response to adaptation. They also calculated the change in frequency of adaptive alleles within each patch during the Semi-Connected with Local Selection epoch. This analysis focused on specific loci (L6-L10) that contained a mix of adaptive and neutral alleles. L6 contained two adaptive alleles and 3 neutral, while L7-L10 each contained one adaptive allele and 4 neutral. Some of the adaptive alleles were advantageous in half of the patches, while others were advantageous in the other half. The authors tracked the frequency of these alleles in order to examine the effect of local adaptation on allele frequency in combination with asymmetric migration from adjacent patches, where the opposite allele was advantageous.

Line no. 309-314

Authors ran 80 simulations on a Dell PowerEdge R820 server, and each individual model run took approximately four hours of processing time to complete. The authors suggest that the simulations could likely be replicated more quickly on a modern desktop or laptop computer, and that the use of the server simply made it possible to run all simulations simultaneously. The HexSim application and the simulation models described in the study are available at [www.hexsim.net](http://www.hexsim.net/).

It is worth noting that the computational effort required for a study can vary significantly depending on the complexity of the models and the number of simulations run. Providing more information about the specific models and parameters used in the simulations, as well as software environment in which they were run, would be helpful for readers to better understand the computational demands of the study.

Line 315-329

It appears that the age-class structure in the study resulted in an average age of reproducing females of 8.7 years, and each landscape epoch of 1000 simulation time steps (Fig 1) spanned approximately 115 generations. During the Semi-Connected epoch, the authors observed that migration rate between adjacent patches varied with dispersal distance, barrier gap permeability, and patch size (Fig 2). They found that the majority of individuals remained in their natal patch, and that migration rates were higher when individuals were assigned the long-distance dispersal behavior and the barrier gaps were highly permeable. The authors also observed that unequal perimeter-area ratios resulted in asymmetric migration, with smaller patches generating proportionately greater numbers of emigrants and larger patches receiving proportionately higher numbers of immigrants.

Line 330-345

the results of a study that analyzed the effect of landscape and dispersal behaviors on gene flow in a population. The results indicate that short dispersal distances were associated with a higher degree of genetic relatedness (i.e., inbreeding) compared to long dispersal distances, as shown by the observed dispersal kernels. Additionally, the effectiveness of gene flow at mixing subpopulations was found to be influenced by landscape history and dispersal behavior, as well as by the permeability of barrier gaps and the strength of selection in certain landscape epochs.

Line 353-390

Authors discussing the results of some simulations or experiments involving population genetics. The purpose of the simulations or experiments seems to be to examine how different factors, such as dispersal distance and barrier gap permeability, influence the genetic structure of a population. The text mentions three different epochs: Continuous, Isolated Patches, and Semi-Connected. It appears that in the Continuous epoch, the population is treated as a single entity, while in the Isolated Patches epoch, the population is divided into distinct patches that are isolated from each other by absolute barriers. In the Semi-Connected epoch, the barriers between the patches are partially permeable, allowing for some level of gene flow between the patches. This mentions that the genetic structure of the population changes depending on the level of gene flow between the patches and the distance that individuals are able to disperse. It also mentions that two different methods, Evanno's method (E) and the visual interpretation of likelihood probabilities (L(K)), were used to infer the number of genetic subpopulations present in the population. It appears that the results of these methods varied depending on the dispersal distance and epoch, with Evanno's method tending to produce inflated estimates of the number of genetic subpopulations in the Isolated Patches epoch.

Line 391-412

some simulations or experiments that were conducted to examine the effect of different factors on the genetic structure of a population. The factors being studied include patch size, barrier gap permeability, and dispersal distance. The text mentions three different epochs: Continuous, Isolated Patches, and Semi-Connected. It appears that in the Continuous epoch, the population is treated as a single entity, while in the Isolated Patches epoch, the population is divided into distinct patches that are isolated from each other by barriers. In the Semi-Connected epoch, the barriers between the patches are partially permeable, allowing for some level of gene flow between the patches.

This mentions that per-capita homozygosity, which is a measure of the proportion of an individual's genome that is made up of identical alleles, varies depending on the epoch and patch size. It appears that homozygosity increases during the Isolated Patches epoch, particularly in small patches, but decreases during the Semi-Connected epoch. the loss of allelic richness and evenness, which are measures of the diversity of alleles within a population, is more pronounced in small patches and during short-distance dispersal.

Line 413-439

simulations or experiments that were conducted to examine the effect of different factors on the evolution of a population. The factors being studied include patch size, barrier gap permeability, dispersal distance, and selection pressure. The text mentions that selection pressure was initiated at a certain time step, and that the population size and carrying capacity emerge mechanistically from the model. It appears that the population size is more stable in large patches and more prone to extinction in small patches, particularly when dispersal distances are short and movement barriers are present.

The frequency of adaptive alleles at certain loci increases globally across all subpopulations as selection acts locally and migration moves the adaptive alleles across the landscape. However, the effect of local selection at one of the loci is counteracted by asymmetric migration between patches of different habitat types. It appears that the extent to which selection is swamped by migration varies depending on patch size, strength of selection, barrier gap permeability, and dispersal distance.

Line 448-462

the challenges of using resistance surfaces, which are used to model gene flow in landscape genetics, to infer the influence of landscape elements on gene flow. The text mentions that resistance surfaces are limited in their ability to capture complex movement behavior and dynamic landscape histories, which can make it difficult to identify causal processes and detect past gene flow.

The simulations can be used to examine the cumulative effects of gene flow, genetic drift, and selection on genetic distance between individuals over several hypothetical landscape histories. It appears that long-distance dispersal behavior can swamp the influence of landscape history on genetic distance when landscape connectivity is high, making it difficult to identify past landscape discontinuities when dispersal distances are long relative to the spatial extent of the study area.

Line 463-476

the challenges of inferring the number of populations from analysis of genetic clustering patterns, and the limitations of popular statistical methods for choosing between possible numbers of genetic clusters. The text mentions that small amounts of interbreeding between close relatives, known as inbreeding depression (IBD), can inflate such inferences and lead to overestimation.

the authors have developed a methodology that allows migration rates to emerge from species-landscape interactions, and that the model demonstrates how alteration of barrier gap permeability can produce departures from the theoretical guideline of "one migrant per generation" for re-establishing genetic panmixia. authors mentions that with short-distance dispersal behavior, it can be difficult to disentangle past genetic history from contemporary genetic structure.

Line 477-497

the challenges of forecasting genetic degradation in small populations facing extinction, and the usefulness of eco-evo simulators in addressing these challenges. The rare alleles are lost from small patches, even when the landscape is fully connected, and that the severity of this loss is more extreme when individuals disperse short distances. the loss of allelic evenness exhibits different patterns depending on patch size, with single allele fixation eventually approaching zero in small patches, but varying depending on initial conditions and not dispersal distance in large patches. In medium patches, the loss of allelic evenness increases with limiting dispersal distance, but the steady-state depends on initial conditions.

This suggests that these findings demonstrate how eco-evo simulators can add realism to the forecasts of genetic degradation used in conservation, and how future studies could improve our understanding of inbreeding and outbreeding depression, explore the relative importance of genetic versus demographic components of viability, and more.

Line 495-511

discussing the capabilities of the HexSim modeling platform to capture the interplay between local selection and asymmetric migration in a spatially discontinuous landscape. The adaptive alleles increase in frequency due to local selection, but that this effect is not observed in small and medium-sized patches when dispersal distance is short and barrier gap permeability is low, likely due to the influence of stochastic drift. when selection is weak, asymmetric migration between large and proximate smaller patches produces highly variable changes in the frequency of the locally adaptive allele in the smaller patches. Even in large patches, where selection might be expected to act efficiently, the anticipated changes in adaptive alleles were only observed when dispersal ability was limited and barrier gap permeability was low. authors suggests that this interplay between local selection and asymmetric migration demonstrates what might be expected as species' ranges shift due to climate change

Line 512-557

The results of the study suggest that advanced software tools like HexSim can be used to explore complex biological mechanisms while retaining species-landscape and intraspecific interactions. The study also highlights the limitations of existing theories in the fields of landscape genetics, population genetics, conservation biology, and evolutionary ecology due to simplifying assumptions. The authors suggest that future studies could improve models by challenging these assumptions and that the HexSim modeling platform could be used to investigate mode of inheritance, linkage between alleles, initial geographic distributions of allele frequencies, and multiple mutation regimes in relation to landscape spatial patterns and movement. The study also suggests that the methodology could be extended to capture intermediate relationships between species and their environments, and to simulate complex mechanisms of intraspecific interactions. Overall, the study suggests that eco-evo theories of the future will better explain patterns and processes when landscapes are spatially and temporally dynamic, species' life histories and genetics are complex and interacting, and when the details matter.

Top of Form
