# Supplementary material for: Adding pattern and process to eco-evo theory and applications
Source: PLoS One. 2023 Mar 9;18(3):e0282535. doi: 10.1371/journal.pone.0282535 (PMC9997922; doi:10.1371/journal.pone.0282535)
Supplement: Attachment — Submitted filename: Response to Reviewers.docx [file pone.0282535.s004.docx]

**Response to Reviewers**

**PONE-D-22-20501_R1**

Reviewer-1 developed a 9-page document as part of their assessment of our revised manuscript. This document contains 25 sections, which we refer to below as *statements*. Of the 25 statements, 4 concern our Introduction, 11 concern our Methods section, and 10 concern our Results section.

Every reviewer statement is accompanied by a range of line numbers, and begins with an overview of what we said in that portion of the manuscript. These passages are largely copied directly from our text, but the reviewer has sprinkled in some of their own words here and there.

No comments, questions, assertions, etc. have been added to the 14 statements that are matched to portions of our Introduction or Results sections. All of the reviewer’s feedback is contained within the 11 statements that pertain to our Methods section. It appears the reviewer approached their task by (A) developing summaries, principally using our words, of most subsections within our Introduction, Methods, and Conclusions sections; and then (B) adding a comment to each summary that pertained to our Methods section. As a result, we have only provided nontrivial responses to the 11 reviewer statements that pertain to our Methods.

All 11 of these statements are essentially the same. They point out where our model lacks nuance that might be found in real landscapes and actual species.

The reviewer misses the point of our manuscript entirely. Yes, our model is simple by design, and by comparison to a complex natural system. We have intentionally designed it this way in order to maximize its generality. The simplicity and generality of our model are assets, and features that allow us to develop results and conclusions with relevance to all four focal disciplines discussed throughout our manuscript (landscape genetics, population genetics, conservation biology, evolutionary ecology). As is done in scientific research generally, we are beginning with simple theories and models; we will and add complexity in time, and then explore the veracity of those more nuanced models. That being said, our simple simulator already captures more demographic nuance, plus species-landscape and species-species interactions, than the majority of existing genetic models.

I was an associate editor Landscape Ecology for eight years, and have spent decades reviewing papers for journals. From that perspective, I found this reviewer’s comments did not reflect an interest in understanding our improving our study; rather, they seemed purely intended to disparage our work. The reviewer’s comments belie a lack of understanding and appreciation of even the most basic elements of our study. They appear disingenuous in that they don’t attempt to critique our work, but instead read as a rushed and inexperienced attempt to discredit it. In fact, it seems the reviewer likely intended to pen 14 additional concerns (4 for the Introduction and 10 for the Results), but just couldn’t think of anything to say.

Below, we address each of the reviewers 25 statement separately, in detail. We first copy the reviewer statements exactly as they were provided to us. We then add our response to the end of each statement. There is repetition in the reviewer statements, and thus also in our responses.

**Reviewer Statement 1**

Line 72-80

you provided that the field of landscape genetics is relatively new and still evolving, and as a result, the available landscape genetics modeling platforms may have limited spatial, demographic, or behavioral sophistication, or may not be able to incorporate mechanisms underlying species-landscape interactions or simultaneously simulate multiple interacting eco-evo drivers of gene-flow. This could potentially limit the ability of these models to accurately simulate complex movements and resulting gene-flow, and may restrict their ability to fully capture the influence of landscape pattern on gene-flow. Another is that the standard approach of comparing inter-individual genetic distance to metrics of landscape structure, such as cost-distance across a resistance surface, is it sufficient for fully understanding the influence of landscape pattern on gene-flow, and may not adequately account for other factors that may also be important in shaping gene-flow.

***Authors’ Response:***

Here, the reviewer is restating what we wrote in this portion of the manuscript. There does not appear to be a question, comment, or actionable statement for us to respond to.

**Reviewer Statement 2**

Line 82-92

the classic population genetic models often assume that complex biological forces such as selection and mutation rates are spatially constant and may not adequately account for spatial heterogeneity and dynamics that may influence population size, density, dispersal flux, or local adaptation. Additionally, these models often reduce spatial complexity to discrete populations and characterize migration processes using a single univariate input parameter, which may not adequately capture the complexity of movement and mating behavior that may govern gene-flow. These simplifications may facilitate the generation of theory, but may also limit the ability of these models to accurately represent systems in which space is heterogeneous and dynamic or gene-flow is governed by complex movement and mating behavior. such systems may be limited and may not adequately account for the influence of landscape structure or non-random mate-choice on gene-flow.

***Authors’ Response:***

Here, the reviewer is restating what we wrote in this portion of the manuscript. There does not appear to be a question, comment, or actionable statement for us to respond to.

**Reviewer Statement 3**

Line 94-103

you provided that existing tools and models for forecasting population viability, such as RAMAS GIS or Vortex, often simplify the linkages between ecological and evolutionary processes and is it capable of simulating complex interacting demographic and genetic traits. This may limit their ability to accurately forecast how landscape changes will impact a population's size, distribution, mate-finding, or natural selection, and may reduce the utility of their forecasts for conservation purposes. Additionally, the relative importance of demography versus genetics in influencing population viability has been actively debated in the conservation literature, and the lack of models or tools that are capable of simultaneously accounting for both of these factors may further hinder the ability to accurately forecast population viability in the face of landscape change.

***Authors’ Response:***

Here, the reviewer is restating what we wrote in this portion of the manuscript. There does not appear to be a question, comment, or actionable statement for us to respond to.

**Reviewer Statement 4**

Line 104-130

The inclusion of detailed spatial patterns and biologically realistic processes in eco-evo models may substantially enhance their realism and defensibility, but may also complicate the models, especially those that are already mechanistically-rich and integrate demography, movement, and gene flow. This complexity may make it more difficult for readers to evaluate the assertions made in these models and to understand the mechanics involved in incorporating spatial structure into genetic models. Additionally, the trade-off between realism, in the form of landscape structure and movement behavior, and mechanistic clarity in the pursuit of enhanced realism may limit the ability of these models to fully capture the complexity of eco-evo processes in natural systems.

***Authors’ Response:***

Here, the reviewer is restating what we wrote in this portion of the manuscript. There does not appear to be a question, comment, or actionable statement for us to respond to.

**Reviewer Statement 5**

Line 134-146

The study is based on a simple heuristic simulation constructed within the HexSim modeling environment, which is a user-friendly, spatially-explicit, individual-based, demo-genetic modeling environment. This may limit the complexity and realism of the model and the range of processes it can capture, potentially leading to a lack of accuracy or generalizability in the results. Additionally, the use of a single species, minimally-complex landscape structure, and a limited number of treatment combinations may not adequately represent the complexity of natural systems and the many factors that can influence eco-evo dynamics. Is this study applicable to other systems ?

***Authors’ Response:***

The first half of the reviewer’s statement is a summary of content we provide in the manuscript. The second half contains a query regarding whether our use of a single species and a simple abstract landscape prevents our study from capturing the complexity found in natural systems.

Yes, our model is simple by design, and by comparison to a complex natural system. We have intentionally designed it this way in order to maximize its generality. The simplicity and generality of our model are assets, and the features that allows us to develop results and conclusions with relevance to all four focal disciplines discussed throughout our manuscript (landscape genetics, population genetics, conservation biology, evolutionary ecology). As is done in scientific research generally, we begin here with simple theories and models; we will and add complexity in time, and then explore the veracity of those more nuanced models.

As the reviewer correctly points out, we have clearly stated in our manuscript (in this and other locations) that our goal was to construct and explore a simple and heuristic demo-genetic simulation model. We designed our study to help readers fully grasp and adopt our novel methods, without becoming distracted by myriad landscape- or species-specific details. This is very standard and highly appropriate when developing methods papers such as ours. That being said, our simple simulator already captures more demographic nuance, plus species-landscape and species-species interactions, than the majority of existing genetic models.

We designed our manuscript to inspire readers from multiple disciplines, and encourage them to adopt and apply our methods. These future applications (including pending studies of our own) will employ actual landscapes and make use of empirically-derived demographic and genetic information. In the current study, we provide a roadmap for these future studies -- a roadmap that is easy to follow because we have limited the complexity of our heuristic model system. Readers who are interested in merging our methods with detailed species and landscape information need only turn to the HexSim website for inspiration and relevant examples. At that website, we provide a list of almost 70 peer-reviewed publications in which HexSim has already been applied in a wide range of settings and systems.

In summary, the reviewer’s observation reflects a key design feature of our study – one that we feel is appropriate, and has been thoughtfully articulated throughout the manuscript. We do not to suggest that our simple heuristic system is realistic. In contrast, we designed and used it as a vehicle for exploring our novel methods, and illustrating how a better accounting for the role of space in the four focal disciplines might help generate future scientific advancements.

For these reasons, we have not changed the manuscript in response to this reviewer statement.

**Reviewer Statement 6**

Line 148-158

the landscape used in the study is artificial and simplified, consisting of only six habitat patches of different sizes that are isolated from a surrounding non-habitat matrix. Is it accurately represent real landscapes, which are typically more complex and diverse and is it include a variety of habitat types and structures ?. Additionally, the use of movement barriers to isolate the patches from each other at certain times and allow limited dispersal at other times accurately represent or not the natural barriers and corridors that influence gene flow in real systems? . Additionally, the use of a uniform potential for crossing all patch interfaces accurately represent or not represent the heterogeneous nature of real landscapes and the different costs and benefits of movement between different patches.

Is the findings of this study applicable to other systems or fully capture the complexity of eco-evo processes in nature?

***Authors’ Response:***

These reviewer questions are quite similar to those from *Reviewer Statement 5*, above. For background, please see our *Authors Response* to *Reviewer Statement 5*.

We designed our study so that the primary results could be anticipated, even as these outcomes change depending on the study *epoch* (see manuscript). Specifically, the use of six landscape patches arrayed as they are, including barriers we could carefully control, allowed us to search for cases where our spatial IBM’s emergent properties departed from the expectations of existing theories and methods.

Of course, we are not suggesting that our landscape or barriers mimic those found in the real world. The reviewer here misses the point of our manuscript entirely. The purpose of our study is to compare new models that carefully account for space to old ones that use simplifying assumptions to avoid having to do so. Our study landscape is ideally suited to this purpose. In essence, the reviewer is suggesting we might have instead written a completely different manuscript. Such manuscripts will be written, but they will rely on this work as a foundation.

For these reasons, we have not changed the manuscript in response to this reviewer statement.

**Reviewer Statement 7**

Line 166-190

It is not clear from this description what the specific goals of the study or how the use of a heuristic simulation with a simple, artificial landscape and a limited set of life history events and species-landscape interactions is intended to contribute to the understanding of eco-evo dynamics. Additionally, the use of uniformly distributed reproductive rates and resource acquisition from a roughly circular neighborhood may accurately reflect or not reflect the complexities of natural systems. It would be helpful to have more information about the intended realism of the model and how the chosen design decisions support the goals of the study.

Overall, it would be useful to consider ways of improving the realism and complexity of this simulation, such as incorporating mechanisms for mutation and more nuanced models of adaptation.

***Authors’ Response:***

We initially articulate the goals of the study in the last paragraph of the Introduction. The lines (166-190) cited by the reviewer are in fact part of our Methods section. In this last paragraph of the Introduction, we state:

Below, we describe a relatively simple theoretical model designed to illustrate how the explicit inclusion of detailed spatial patterns and biologically realistic processes might facilitate the development of new eco-evo theory and applications. We demonstrate model relevance by highlighting results that (a) illustrate a priori expectations of a core eco-evo dynamic fundamental to a discipline, and (b) illustrate how additional biological and spatial realism might contribute to new theory and improve our confidence in modeling applications (Table 1). Our primary goals include both evaluating and articulating the benefits derived from adding spatial structure to genetic models, and illustrating the mechanics involved in doing this well. While the incorporation of space can substantially enhance realism and defensibility, it also complicates models, especially those which are already mechanistically-rich (e.g., that integrate demography, movement, and gene flow). In pursuit of the former goal, we trade away elements of realism, in the form of landscape structure and movement behavior, for mechanistic clarity. Readers evaluating our assertions will benefit from the simplicity of our simulations, as the number of drivers and responses they must track is limited. In regards to the latter goal, our work constitutes an important extension to the existing descriptions and applications of HexSim [14]. Readers interested in adopting this software will benefit from the case studies discussed here, as they provide detail on HexSim genetics that is unavailable in previous publications.

We suggest that this statement describing our study goals is exceedingly clear, and that we also have used this paragraph to articulate our landscape and species life history design criteria. As it is very traditional to place such a paragraph at the end of a paper’s Introduction section, we’re a bit perplexed regarding how the reviewer missed this content.

The reviewer goes on to suggest that:

- It would be helpful to have more information about the intended realism of the model and how the chosen design decisions support the goals of the study.
- Overall, it would be useful to consider ways of improving the realism and complexity of this simulation, such as incorporating mechanisms for mutation and more nuanced models of adaptation.

In addressing the first of these two points, we suggest that this information is in fact contained in the paragraph from the Introduction that we’ve copied above. Further, we revisit those issues in multiple places throughout the manuscript.

In addressing the second of these two points, we suggest that (again) the reviewer is missing the point of the manuscript entirely. Please see our responses (above) to *Reviewer Statement 5* and *Reviewer Statement 6* for background and further detail.

For these reasons, we have not changed the manuscript in response to this reviewer statement.

**Reviewer Statement 8**

Line 191-205

There are several issues with this method for simulating population genetics and evolution:

1. Using a fixed set of initial allele frequencies and not allowing for mutation means that the gene pool of the population is fixed and not subject to change over time. This means that the population will not be able to adapt to changing environments or selective pressures.
2. The use of pure neutrality for five of the loci may not be realistic, as many loci in real populations are subject to some degree of selective pressure, even if they do not directly impact survival or reproduction.
3. The use of a fixed set of locally adapted alleles may not accurately reflect the complex and multifaceted nature of adaptation in real populations, which can involve a wide range of genetic and environmental factors.
4. The decision to initiate selection at a specific time step (3000) may not be justified and could potentially lead to inflated estimates of the strength of selection.

***Authors’ Response:***

Regarding item 1. The HexSim model can simulate mutation events quite easily and effectively. However, simulating the influence of mutation lies entirely outside the scope of the current study. Our goal here is not to study the evolution of an actual species in a real landscape.

Regarding items 2 & 3. As we explain carefully throughout earlier portions of this *Response to Reviewers* document, the goal of this study was not to simulate a realistic landscape or species.

Regarding item 4. This is a bit of an odd comment, as the reviewer is descending quite far into minutiae and conjecture. We fear the reviewer has not clearly understood the study goals.

For these reasons, we have not changed the manuscript in response to this reviewer statement.

**Reviewer Statement 9**

Line 206-222Top of FormTop of Form

1. Using a binary landscape with habitat patches embedded within a non-habitat matrix accurately reflect or not reflect the complex and varied nature of real landscapes, which can have multiple habitat types and multiple scales of patchiness.
2. Is the use of a fixed set of movement barriers and gap permeabilities adequately capture the dynamic and variable nature of movement in real populations, which can be influenced by a wide range of factors such as individual behavior, habitat quality, and environmental conditions.
3. Is the decision to impose absolute movement barriers justified and could potentially lead to estimates of the strength of drift and isolation by distance.
4. Is the use of habitat types that confer increased fitness to specific genotypes accurately reflect the complex and multifaceted nature of adaptation in real populations, which can involve a wide range of genetic and environmental factors.

***Authors’ Response:***

Regarding items 1 & 2. The reviewer previously articulated these concerns. Please see our responses (above) to *Reviewer Statement 5* and *Reviewer Statement 6*.

Regarding item 3. There are no barriers to movement in Epoch 1. We use absolute movement barriers in Epoch 2, so as to completely separate the six landscape patches. Epochs 3 & 4 use movement barriers that are permeable in places. Of course, changing the nature of the movement barriers can be expected to change the simulation results. Our experiments were designed to illustrate how a model that carefully incorporates spatial structure can produce results that differ from competing non-spatial or pseudo-spatial models. For this purpose, our study’s movement barrier design works exceedingly well.

Regarding item 4. As we have discussed at length throughout this document (in response to similar questions), the goal of this study was not to simulate a realistic landscape or species.

For these reasons, we have not changed the manuscript in response to this reviewer statement.

**Reviewer Statement 10**

Line 223-237

1. The use of per-capita homozygosity as a measure of genetic diversity may not accurately reflect the complexity of real population genetics, as it does not consider factors such as the number and distribution of alleles or the relationships between individuals.
2. The decision to track only individual genotypes and not pedigree information may limit the types of analyses that can be performed, as pedigree information can provide important insights into the relationships between individuals and the patterns of inheritance in a population.
3. The use of generation time as the average age of reproducing females may not accurately reflect the complexity of real population demography, as it does not consider factors such as mortality rates or the age distribution of the population.
4. The definition of migration as the movement of individuals between patches that subsequently reproduce there may exclude important types of movement, such as exploratory or dispersal movements that do not result in reproduction.

***Authors’ Response:***

As we have discussed throughout this document, the goal of this study was not to simulate a realistic landscape or species. These four items are all purely speculative, they focus on levels of biological detail much finer than those our study simulates, and they do not contain specific questions or actionable statements. These assertions do, however, strongly reinforce our concern that the reviewer has failed to both grasp the goals of our study, or appreciate its results and conclusions.

For these reasons, we have not changed the manuscript in response to this reviewer statement.

**Reviewer Statement 11**

Line 239-256

1. The use of visual assessment of correlograms and dispersal kernels may not provide a reliable or objective measure of the degree of isolation by distance in the population.
2. The decision to randomly select a single replicate simulation and a subset of individuals from each patch location may not accurately reflect the overall patterns in the population and could potentially introduce bias.
3. The use of a simple metric of pairwise genetic distance based on the number of differing alleles may not accurately reflect the complexity of real population genetics and could potentially overlook important factors such as the frequencies and distributions of alleles in the population.
4. The use of triangular matrices to visualize pairwise genetic distances may not be the most effective way of representing this data, as it does not clearly show the relationships between all pairs of individuals.

***Authors’ Response:***

The reviewer’s four statements are all speculative, and none could possibly yield a simple definitive answer. These concerns would need to be uniquely reevaluated in every study.

In our simple heuristic study system, the visual assessment of correlograms and dispersal kernels is actually quite appropriate, and it produces accurate assessments of the system dynamics. In more complex systems, this would not necessarily be true.

Similarly, our decision to use results from a single replicate simulation to compute inter-individual genetic distances is reasonable give our study design. In a real-world system where the contemporary distribution of genotypes reflects a long history of landscape change and disturbance, other analytic methods are likely to be more appropriate.

The same is true for our computation of pairwise genetic distances. These points all underscore our previous assertion -- that the heuristic design of our landscape and species is an asset, not a liability as the reviewer seems to repeatedly imply.

Finally, we disagree completely with the reviewer’s statement about our use of triangular matrices to visualize pairwise genetic distances. This method, applied in the construction of Figure 4, captures an enormous amount of information and displays it in format that allows readers to quickly and easily grasp the take-home messages and insights. It is an elegant design.

For these reasons, we have not changed the manuscript in response to this reviewer statement.

**Reviewer Statement 12**

Line 257-276

It appears that the authors used a Bayesian clustering method called STRUCTURE to analyze population genetic structure in this study. They collected genotype data stratified by putative populations (patches), and imported this data into STRUCTURE for analysis. The authors encountered the issue of unequal sample sizes in their patches, which can interfere with the assessment of the number of subpopulations and assignment probabilities in STRUCTURE. To address this issue, they randomly selected a fixed number of individuals (n=25) from each patch for analysis. In cases where there were fewer than 25 individuals in a patch, they included some genotypes multiple times until each patch had a sample of 25, for a total of 150 individuals. The authors tested a range of possible values for the number of unique genetic clusters (K) from 1 to 20, and used two methods to identify the best-supported K values: plotting the replicate average Ln P(D|K) and visually determining the minimum K of the curve's asymptote, and using Evanno's ∆K method. If there was a close tie between the supported K values from these two methods, the authors considered them all for individual assignment analyses.

It is worth noting that using a fixed number of individuals (n=25) from each patch for analysis, regardless of the actual number of individuals present in each patch, may introduce biases into the analysis. Additionally, while the authors mention that they used the default settings of the admixture model and correlated allele frequencies in their STRUCTURE analyses, it would be helpful to provide more detail about these settings and how they were chosen. It would also be useful to explain why the authors expected the number of unique genetic clusters (K) to vary from 1 to 6 based on landscape structure

***Authors’ Response:***

The reviewer uses the first paragraph to restate what we wrote in this section of the manuscript. The reviewer’s comments about program STRUCTURE, in the second paragraph, make no sense to us. Our use of STRUCTURE is very standard, and the results we derived from our STRUCTURE assessments make sense in light of the study landscape and species. STRUCTURE-based analyses account for a very small part of our methods and results, and so we feel the current level of detail we provide regarding these methods is exactly right. All told, the reviewer appears to be simply going through every section of our Methods section, and pointing out every place where any of our techniques might conceivable prove inadequate in a more complex system. We don’t believe this constitutes a meaningful critique of our work.

For these reasons, we have not changed the manuscript in response to this reviewer statement.

**Reviewer Statement 13**

Line 277-299

It appears that the authors calculated Per-capita Homozygosity as the percent of homozygous genotypes across all alleles and individuals within a given landscape patch. They also examined the effect of patch isolation on allele frequencies at the end of the Continuous and Isolated Patches epochs. To do this, they calculated three metrics of genetic degradation for each locus in each subpopulation using the "divBasic" function from the "diveRsity" R package. These metrics were:

1. Allelic richness: the number of unique alleles per locus. All ten loci began with 5 alleles each.
2. Allelic evenness: a measure of the relative frequencies of different alleles at a locus. It is calculated as ∑𝑝𝑖ln(𝑝𝑖)/ ln(5), where 𝑝𝑖 is the frequency of the 𝑖𝑡ℎ allele within each locus, and 𝑙𝑛(5) is the maximum evenness in each locus, given that all were initiated with 5 alleles. The starting population had different initial allele frequencies corresponding to allelic evenness values of 1.0 = "Equal", 0.96 = "Unequal", and 0.72 = "Rare" (Table 2).
3. Heterozygosity deficit: a measure of the deviation of the observed number of heterozygous genotypes from the expected number. It is calculated as 𝐻𝑜𝑏𝑠 −𝐻𝑒𝑥𝑝, where 𝐻𝑜𝑏𝑠 is the number of observed heterozygous genotypes and expected heterozygosity is calculated as: 𝐻𝑒𝑥𝑝 = 1−∑ (𝑞𝑖)2 𝑛 1 , where 𝑛 represents the number of alleles, and 𝑞𝑖 is the frequency of the 𝑖𝑡ℎ allele at a locus. Note that 𝐻𝑒𝑥𝑝 is calculated based on the number of extant alleles at a locus, and will fluctuate dramatically as alleles are lost from a population. Therefore, unlike Allelic richness or Allelic evenness, this metric has a shifting baseline.

For each of these metrics, the authors calculated the mean and standard deviation across all replicates within the same patch size (small, medium, or large) and of the same initial allele frequency (equal, unequal, or rare) for each of the two time points of interest (at the end of the Continuous and Isolated Patches epochs).

It would be helpful to provide more information about the specific populations and the number of replicates used in this analysis, as well as a clear explanation of the reasoning behind the choice of patch sizes and initial allele frequencies.

***Authors’ Response:***

As usual, these reviewer comments, except for the last short paragraph above, are mostly copied directly from our manuscript. They don’t contain any questions or new observations. Regarding the final paragraph: the term “population”, in the sense the reviewer uses the term, is an emergent property of our simulation model. We describe these populations extensively throughout the Results section of our manuscript. Is the reviewer suggesting that we should have placed our results within our Methods section? The manuscript clearly states that we performed 10 replicates per treatment for each HexSim simulation (lines 225-226), and that we performed 20 replicates of each STRUCTURE analysis (lines 270-272). Regarding the reviewer’s comment asking for a clear explanation of the reasoning behind the choice of patch sizes and initial allele frequencies: we have already explained our reasoning in detail. The exact numbers of hexagons per patch, or alleles per locus are arbitrary and irrelevant. What is important is that the reader understands our model system, and has a sense for why it might behave in one way or another. This closing comment strikes us as remarkably disingenuous.

For these reasons, we have not changed the manuscript in response to this reviewer statement.

**Reviewer Statement 14**

Line no 300-308

Authors tracked population size for each patch across all time steps to measure the response to adaptation. They also calculated the change in frequency of adaptive alleles within each patch during the Semi-Connected with Local Selection epoch. This analysis focused on specific loci (L6-L10) that contained a mix of adaptive and neutral alleles. L6 contained two adaptive alleles and 3 neutral, while L7-L10 each contained one adaptive allele and 4 neutral. Some of the adaptive alleles were advantageous in half of the patches, while others were advantageous in the other half. The authors tracked the frequency of these alleles in order to examine the effect of local adaptation on allele frequency in combination with asymmetric migration from adjacent patches, where the opposite allele was advantageous.

***Authors’ Response:***

Here, the reviewer is restating what we wrote in this portion of the manuscript. There does not appear to be a question, comment, or actionable statement for us to respond to.

**Reviewer Statement 15**

Line no. 309-314

Authors ran 80 simulations on a Dell PowerEdge R820 server, and each individual model run took approximately four hours of processing time to complete. The authors suggest that the simulations could likely be replicated more quickly on a modern desktop or laptop computer, and that the use of the server simply made it possible to run all simulations simultaneously. The HexSim application and the simulation models described in the study are available at [www.hexsim.net](http://www.hexsim.net/).

It is worth noting that the computational effort required for a study can vary significantly depending on the complexity of the models and the number of simulations run. Providing more information about the specific models and parameters used in the simulations, as well as software environment in which they were run, would be helpful for readers to better understand the computational demands of the study.

***Authors’ Response:***

The first paragraph above is copied directly from our manuscript. We don’t understand the point of reviewer’s second paragraph, except to note that it is consistent with the overall tone and rigor of the review. Of course, computational effort will vary based on model complexity and the number of replicate simulations. We have already provided extensive detail on our model design and parameters. Does the reviewer want to know if our model runs on Apple, Windows, or Linux? If there was any remaining doubt regarding the intent and veracity of this review, this second paragraph should remove that doubt.

As a result of this comment, we have decided to remove the entire subsection of the manuscript titled “Computational Effort and Model Availability” (formerly lines 309-314). We felt this section didn’t add any substantial value to the manuscript. However, we did copy the following text from that section before deleting it, and placed it to the end of the second paragraph of the Introduction: "Both the HexSim application and the simulation models described here are available at www.hexsim.net".

**Reviewer Statement 16**

Line 315-329

It appears that the age-class structure in the study resulted in an average age of reproducing females of 8.7 years, and each landscape epoch of 1000 simulation time steps (Fig 1) spanned approximately 115 generations. During the Semi-Connected epoch, the authors observed that migration rate between adjacent patches varied with dispersal distance, barrier gap permeability, and patch size (Fig 2). They found that the majority of individuals remained in their natal patch, and that migration rates were higher when individuals were assigned the long-distance dispersal behavior and the barrier gaps were highly permeable. The authors also observed that unequal perimeter-area ratios resulted in asymmetric migration, with smaller patches generating proportionately greater numbers of emigrants and larger patches receiving proportionately higher numbers of immigrants.

***Authors’ Response:***

Here, the reviewer is restating what we wrote in this portion of the manuscript. There does not appear to be a question, comment, or actionable statement for us to respond to.

**Reviewer Statement 17**

Line 330-345

the results of a study that analyzed the effect of landscape and dispersal behaviors on gene flow in a population. The results indicate that short dispersal distances were associated with a higher degree of genetic relatedness (i.e., inbreeding) compared to long dispersal distances, as shown by the observed dispersal kernels. Additionally, the effectiveness of gene flow at mixing subpopulations was found to be influenced by landscape history and dispersal behavior, as well as by the permeability of barrier gaps and the strength of selection in certain landscape epochs.

***Authors’ Response:***

Here, the reviewer is restating what we wrote in this portion of the manuscript. There does not appear to be a question, comment, or actionable statement for us to respond to.

**Reviewer Statement 18**

Line 353-390

Authors discussing the results of some simulations or experiments involving population genetics. The purpose of the simulations or experiments seems to be to examine how different factors, such as dispersal distance and barrier gap permeability, influence the genetic structure of a population. The text mentions three different epochs: Continuous, Isolated Patches, and Semi-Connected. It appears that in the Continuous epoch, the population is treated as a single entity, while in the Isolated Patches epoch, the population is divided into distinct patches that are isolated from each other by absolute barriers. In the Semi-Connected epoch, the barriers between the patches are partially permeable, allowing for some level of gene flow between the patches. This mentions that the genetic structure of the population changes depending on the level of gene flow between the patches and the distance that individuals are able to disperse. It also mentions that two different methods, Evanno's method (E) and the visual interpretation of likelihood probabilities (L(K)), were used to infer the number of genetic subpopulations present in the population. It appears that the results of these methods varied depending on the dispersal distance and epoch, with Evanno's method tending to produce inflated estimates of the number of genetic subpopulations in the Isolated Patches epoch.

***Authors’ Response:***

Here, the reviewer is restating what we wrote in this portion of the manuscript. There does not appear to be a question, comment, or actionable statement for us to respond to.

**Reviewer Statement 19**

Line 391-412

some simulations or experiments that were conducted to examine the effect of different factors on the genetic structure of a population. The factors being studied include patch size, barrier gap permeability, and dispersal distance. The text mentions three different epochs: Continuous, Isolated Patches, and Semi-Connected. It appears that in the Continuous epoch, the population is treated as a single entity, while in the Isolated Patches epoch, the population is divided into distinct patches that are isolated from each other by barriers. In the Semi-Connected epoch, the barriers between the patches are partially permeable, allowing for some level of gene flow between the patches.

This mentions that per-capita homozygosity, which is a measure of the proportion of an individual's genome that is made up of identical alleles, varies depending on the epoch and patch size. It appears that homozygosity increases during the Isolated Patches epoch, particularly in small patches, but decreases during the Semi-Connected epoch. the loss of allelic richness and evenness, which are measures of the diversity of alleles within a population, is more pronounced in small patches and during short-distance dispersal.

***Authors’ Response:***

Here, the reviewer is restating what we wrote in this portion of the manuscript. There does not appear to be a question, comment, or actionable statement for us to respond to.

**Reviewer Statement 20**

Line 413-439

simulations or experiments that were conducted to examine the effect of different factors on the evolution of a population. The factors being studied include patch size, barrier gap permeability, dispersal distance, and selection pressure. The text mentions that selection pressure was initiated at a certain time step, and that the population size and carrying capacity emerge mechanistically from the model. It appears that the population size is more stable in large patches and more prone to extinction in small patches, particularly when dispersal distances are short and movement barriers are present.

The frequency of adaptive alleles at certain loci increases globally across all subpopulations as selection acts locally and migration moves the adaptive alleles across the landscape. However, the effect of local selection at one of the loci is counteracted by asymmetric migration between patches of different habitat types. It appears that the extent to which selection is swamped by migration varies depending on patch size, strength of selection, barrier gap permeability, and dispersal distance.

***Authors’ Response:***

Here, the reviewer is restating what we wrote in this portion of the manuscript. There does not appear to be a question, comment, or actionable statement for us to respond to.

**Reviewer Statement 21**

Line 448-462

the challenges of using resistance surfaces, which are used to model gene flow in landscape genetics, to infer the influence of landscape elements on gene flow. The text mentions that resistance surfaces are limited in their ability to capture complex movement behavior and dynamic landscape histories, which can make it difficult to identify causal processes and detect past gene flow.

The simulations can be used to examine the cumulative effects of gene flow, genetic drift, and selection on genetic distance between individuals over several hypothetical landscape histories. It appears that long-distance dispersal behavior can swamp the influence of landscape history on genetic distance when landscape connectivity is high, making it difficult to identify past landscape discontinuities when dispersal distances are long relative to the spatial extent of the study area.

***Authors’ Response:***

Here, the reviewer is restating what we wrote in this portion of the manuscript. There does not appear to be a question, comment, or actionable statement for us to respond to.

**Reviewer Statement 22**

Line 463-476

the challenges of inferring the number of populations from analysis of genetic clustering patterns, and the limitations of popular statistical methods for choosing between possible numbers of genetic clusters. The text mentions that small amounts of interbreeding between close relatives, known as inbreeding depression (IBD), can inflate such inferences and lead to overestimation.

the authors have developed a methodology that allows migration rates to emerge from species-landscape interactions, and that the model demonstrates how alteration of barrier gap permeability can produce departures from the theoretical guideline of "one migrant per generation" for re-establishing genetic panmixia. authors mentions that with short-distance dispersal behavior, it can be difficult to disentangle past genetic history from contemporary genetic structure.

***Authors’ Response:***

Here, the reviewer is restating what we wrote in this portion of the manuscript. There does not appear to be a question, comment, or actionable statement for us to respond to.

**Reviewer Statement 23**

Line 477-497

the challenges of forecasting genetic degradation in small populations facing extinction, and the usefulness of eco-evo simulators in addressing these challenges. The rare alleles are lost from small patches, even when the landscape is fully connected, and that the severity of this loss is more extreme when individuals disperse short distances. the loss of allelic evenness exhibits different patterns depending on patch size, with single allele fixation eventually approaching zero in small patches, but varying depending on initial conditions and not dispersal distance in large patches. In medium patches, the loss of allelic evenness increases with limiting dispersal distance, but the steady-state depends on initial conditions.

This suggests that these findings demonstrate how eco-evo simulators can add realism to the forecasts of genetic degradation used in conservation, and how future studies could improve our understanding of inbreeding and outbreeding depression, explore the relative importance of genetic versus demographic components of viability, and more.

***Authors’ Response:***

Here, the reviewer is restating what we wrote in this portion of the manuscript. There does not appear to be a question, comment, or actionable statement for us to respond to.

**Reviewer Statement 24**

Line 495-511

discussing the capabilities of the HexSim modeling platform to capture the interplay between local selection and asymmetric migration in a spatially discontinuous landscape. The adaptive alleles increase in frequency due to local selection, but that this effect is not observed in small and medium-sized patches when dispersal distance is short and barrier gap permeability is low, likely due to the influence of stochastic drift. when selection is weak, asymmetric migration between large and proximate smaller patches produces highly variable changes in the frequency of the locally adaptive allele in the smaller patches. Even in large patches, where selection might be expected to act efficiently, the anticipated changes in adaptive alleles were only observed when dispersal ability was limited and barrier gap permeability was low. authors suggests that this interplay between local selection and asymmetric migration demonstrates what might be expected as species' ranges shift due to climate change

***Authors’ Response:***

Here, the reviewer is restating what we wrote in this portion of the manuscript. There does not appear to be a question, comment, or actionable statement for us to respond to.

**Reviewer Statement 25**

Line 512-557

The results of the study suggest that advanced software tools like HexSim can be used to explore complex biological mechanisms while retaining species-landscape and intraspecific interactions. The study also highlights the limitations of existing theories in the fields of landscape genetics, population genetics, conservation biology, and evolutionary ecology due to simplifying assumptions. The authors suggest that future studies could improve models by challenging these assumptions and that the HexSim modeling platform could be used to investigate mode of inheritance, linkage between alleles, initial geographic distributions of allele frequencies, and multiple mutation regimes in relation to landscape spatial patterns and movement. The study also suggests that the methodology could be extended to capture intermediate relationships between species and their environments, and to simulate complex mechanisms of intraspecific interactions. Overall, the study suggests that eco-evo theories of the future will better explain patterns and processes when landscapes are spatially and temporally dynamic, species' life histories and genetics are complex and interacting, and when the details matter. Top of Form

***Authors’ Response:***

Here, the reviewer is restating what we wrote in this portion of the manuscript. There does not appear to be a question, comment, or actionable statement for us to respond to.
